# An Expressed Sequence Tag (EST)-enriched genetic map of turbot (*Scophthalmus maximus*): a useful framework for comparative genomics across model and farmed teleosts

**DOI:** 10.1186/1471-2156-13-54

**Published:** 2012-07-02

**Authors:** Carmen Bouza, Miguel Hermida, Belén G Pardo, Manuel Vera, Carlos Fernández, Roberto de la Herrán, Rafael Navajas-Pérez, José Antonio Álvarez-Dios, Antonio Gómez-Tato, Paulino Martínez

**Affiliations:** 1 Departamento de Genética, Facultade de Veterinaria, Universidade de Santiago de Compostela (USC), Campus de Lugo, 27002, Lugo, Spain; 2 Departamento de Genética, Facultad de Ciencias, Universidad de Granada, 18071, Granada, Spain; 3 Departamento de Matemática Aplicada, (USC), Facultade de Matemáticas, Universidade de Santiago de Compostela, 15782, Santiago de Compostela, Spain; 4 Departamento de Geometría y Topología, Facultad de Matemáticas, Universidade de Santiago de Compostela, 15782, Santiago de Compostela, Spain

**Keywords:** Turbot, Scopththalmus maximus, Pleuronectiformes, Genetic map, Recombination frequency, Comparative mapping

## Abstract

**Background:**

The turbot (*Scophthalmus maximus*) is a relevant species in European aquaculture. The small turbot genome provides a source for genomics strategies to use in order to understand the genetic basis of productive traits, particularly those related to sex, growth and pathogen resistance. Genetic maps represent essential genomic screening tools allowing to localize quantitative trait loci (QTL) and to identify candidate genes through comparative mapping. This information is the backbone to develop marker-assisted selection (MAS) programs in aquaculture. Expressed sequenced tag (EST) resources have largely increased in turbot, thus supplying numerous type I markers suitable for extending the previous linkage map, which was mostly based on anonymous loci. The aim of this study was to construct a higher-resolution turbot genetic map using EST-linked markers, which will turn out to be useful for comparative mapping studies.

**Results:**

A consensus gene-enriched genetic map of the turbot was constructed using 463 SNP and microsatellite markers in nine reference families. This map contains 438 markers, 180 EST-linked, clustered at 24 linkage groups. Linkage and comparative genomics evidences suggested additional linkage group fusions toward the consolidation of turbot map according to karyotype information. The linkage map showed a total length of 1402.7 cM with low average intermarker distance (3.7 cM; ~2 Mb). A global 1.6:1 female-to-male recombination frequency (RF) ratio was observed, although largely variable among linkage groups and chromosome regions. Comparative sequence analysis revealed large macrosyntenic patterns against model teleost genomes, significant hits decreasing from stickleback (54%) to zebrafish (20%). Comparative mapping supported particular chromosome rearrangements within Acanthopterygii and aided to assign unallocated markers to specific turbot linkage groups.

**Conclusions:**

The new gene-enriched high-resolution turbot map represents a useful genomic tool for QTL identification, positional cloning strategies, and future genome assembling. This map showed large synteny conservation against model teleost genomes. Comparative genomics and data mining from landmarks will provide straightforward access to candidate genes, which will be the basis for genetic breeding programs and evolutionary studies in this species.

## Background

The turbot (*Scophthalmus maximus*) is a flatfish of great commercial value, which represents one of the most promising marine species of European aquaculture. Production reached 9,142 t in 2009 [1], and it is predicted to double up in size in 2014. Turbot has also become very popular in the Chinese market, and production in this country has been reported around 50,000 t in 2006 [2]. Genetic breeding programs are being carried out by several turbot companies supported by microsatellite parentage tools [3]. Increasing growth rate, controlling sex ratio (females largely outgrow males) and enhancing disease resistance currently constitute the main goals of genetic breeding programs in this species.

The small turbot genome (C value: 0.86 pg; http://www.genomesize.com/fish.htm) is organized in 2n = 44 chromosomes with no sex-associated chromosome heteromorphism [4,5]. An important investment effort has been devoted in the recent years to increase genomic resources in this species to provide new molecular tools to support genetic breeding programs. An Expressed Sequence Tag (EST) database constructed using cDNA libraries from immune tissues [6,7] has been recently enriched using new generation sequencing (NGS) technologies and currently contains 35,000 contigs and 65,000 singletons. This database was used to design the first turbot oligo-microarray [8], which enabled to identify differentially expressed (DE) genes for pathogen resistance [9,10]. Co-localization of DE genes through comparative mapping with disease-resistance QTL constitutes a primary goal to identify candidate genes for resistance to pathogens [11]. EST databases are essential not only for functional annotation, but also for the identification of gene-associated markers (type I [6]). New microsatellites and single nucleotide polymorphisms (SNP) originating from the EST database have recently been developed in turbot [7,12,13]. These markers were used to identify candidate genes subjected to divergent selection [14], and to begin constructing an EST-linked genetic map in this species [12]. Finally, a 5X BAC genomic library containing ~46.000 clones of ~125 kb on average has been constructed and it is being exploited for physical mapping of specific genomic regions (B. Pardo, unpublished data).

Genetic maps are essential tools to locate genomic regions associated with productive characters, which can eventually be applied in marker-assisted selection programs or used to identify genes related to specific traits through fine mapping and/or positional cloning strategies [15-17]. Additionally, they provide the support to study genome organization and evolution through comparative mapping, and provide useful landmarks for genome assembly [18-24]. A first generation turbot consensus map (242 anonymous microsatellites; 26 linkage groups (LG)) was reported by Bouza *et al.*[25]. It has been used to identify QTL for sex determination [26], growth rate [27] and resistance to pathogens [28,29]. Recently, a new microsatellite genetic map has been reported by Ruan *et al.*[2] using 158 anonymous markers.

Genomic resources have greatly increased in aquaculture species especially after the arrival of NGS, and several genome projects are underway in several fish species (http://www.genomesonline.org/cgi-bin/GOLD/index.cgi). However, most comparative genomic studies still rely on model species. Genome sequences with high coverage are available in zebrafish (*Danio rerio*), fugu (*Fugu rubripes*), Tetraodon (*Tetraodon nigroviridis*), medaka (*Oryzias latipes*) and stickleback (*Gasterosteus aculeatus*) (http://www.ensembl.org). Since gene-associated markers are much more conserved than anonymous ones, they constitute the preference target to go further on comparative mapping and evolutionary genomics [24,30,31]. Comparative mapping also represents the best strategy to capture candidate genes at genomic regions associated with productive characters in aquaculture species [32-35].

The aim of this study was to enrich the turbot genetic map using EST-linked markers to create a more powerful tool for comparative genomic and evolutionary studies in turbot. This second-generation genetic map will be useful for identifying candidate genes associated to productive traits and for marker-assisted selection in genetic breeding programs for turbot industry.

## Results and discussion

### Genetic markers and segregation analysis

The existence of a three-generation pedigree facilitated the consistent detection of null alleles. Among the 463 informative mapping markers ( Additional file 1: Table S1), 20 loci (4.3%), 18 microsatellites (4.6%) and two SNP (2.7%) showed null alleles in any of the eight diploid families (DF and QF1-7), in accordance with previous data [3,7]. Deviations from Mendelian expectations were detected at 27.5% loci (P < 0.05) mostly due to SNP (24.7% over 91 tests, P < 0.05) than to microsatellites (10.8% over 916 tests, P < 0.05) as previously reported in turbot [7,25]. As suggested [36], the existence of paralogous genes due to the teleost gene duplication probably interferes with SNP genotyping, hence the higher proportion of Mendelian deviations observed. However, this fact did not determine a lower mapping success at deviated loci, showing a very similar proportion of framework markers in the turbot map as the non-deviated ones (74.8% *vs* 72.4%).

### The turbot consensus map

**Figure 1:**
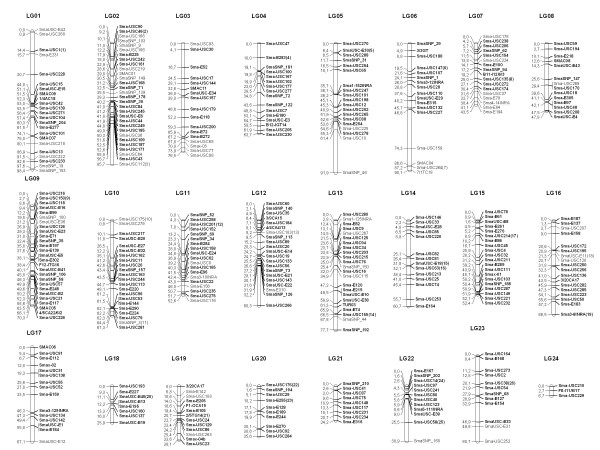
**Consensus turbot map.** Framework markers in bold characters; accessory markers indicated by parentheses beside the closest marker and listed at the end of Additional file 5: Figure S2; LOD < 3 markers in normal type.

The use of several mapping families has the advantage of increasing the number of informative meiosis, especially useful for low polymorphic markers, and also enables the comparison between genetic maps of different families or sexes. New genetic markers (mostly EST-linked), in addition to those previously reported [12,25], were used to construct nine family maps to be integrated in a new consensus map. A large set of common informative markers were used to anchor the different family maps in order to integrate them into a single consensus map ( Additional file 2: Table S2). This map consisted of 24 linkage groups named LG1 to LG24 (Figure 1). Markers in homologous linkage groups were compared among family maps ( Additional file 3: Figure S1), full collinearity being observed at 13 linkage groups and very minor discrepancies at the 11 remaining ones, always involving closely linked markers (mostly < 3 cM).

The resulting consensus map (Figure 1) contained 438 out of 463 informative markers (94.4%), 180 EST-linked (41.1%) and 258 anonymous (58.9%) (Table 1). Among them, 336 were framework (72.4%), 63 mapped at LOD < 3 (13.6%), 39 accessory (8.4%) and 26 remained unlinked (5.6%). The 24 linkage groups of the consensus map represent a reduction from the previous 26 ones [25] in the way towards the expected 22 linkage groups according to turbot karyotype (n = 22; [4,5]). Thus, groups LG4 and LG25 and groups LG10 and LG26, respectively, merged into single groups named LG4 and LG10 in the new consensus map (Figure 1). These two fusions had been suggested only based on paternal segregation data by Bouza *et al.*[25]. Additionally, some markers shared by different linkage groups suggested two additional fusions between LG8 and LG18 and between LG16 and LG19. If these fusions were confirmed, it would represent the final convergence to the expected 22 linkage groups.

**Table 1:** Genetic markers and map and genome length in the turbot maps

	**Consensus**	**Paternal**	**Maternal**
Total markers	463	–	–
Mapped markers	438	221	241
EST-linked	180	55	60
Anonymous	258	165	181
Framework markers	336	215	225
EST-linked	115	55	49
Anomynous	211	159	176
LOD < 3 markers	62	6	16
Accesory	39	–	–
Unlinked	26	–	–
Total length	1402.7	854.2	1369,1
Max. distance^*b*^	30.5	24,5	30,4
Mean distance^*c*^	3.7	4,4	6,3
Framework length	1193.4	781.7	1274
Max. distance	28.1	23	30,4
Mean distance	3.8	4.1	6.3
Gen. length^*a*^ (total)	1530,8	1063.4	1596.1
Gen. length^*a*^ (framew)	1375.8	979.2	1576.4

LG24 length was added to obtain total and estimated lengths of paternal and maternal maps for comparison with consensus map. ^*a*^Genome length was estimated according to Hubert and Hedgecock [37]. ^*b*^Max. dist.: Maximum intermarker distance (cM) in each map. ^*c*^Average intermarker distance.

The total map length (1402.7 cM) was very similar to that previously reported [25], but intermarker distance substantially decreased from 6.5 to 3.7 cM, thus the map being among the most dense maps within non-model teleosts [23,31,38-40]. Only four terminal regions involving non-framework markers at LG2, LG5, LG6 and LG12 showed distances higher than 20 cM, a threshold considered relevant for QTL identification [41]. The framework map covered 1193.4 cM, thus approaching the total length estimate. Considering the estimated genome size of the turbot between 600–800 Mb [5,42], the present map would have on average a marker every ~2 Mb, thus representing a very useful tool for QTL identification and positional cloning strategies. Besides, this map will be valuable for physical mapping starting from the available BAC library and for genome assembling in future turbot genome projects.

### Recombination frequency (RF) between sexes and families

RF is a species-specific parameter, but also variable within species according to sex, family, chromosome, and genomic region [43]. These differences constitute an important factor to be considered when constructing genetic maps and when maps are applied for QTL identification and marker assisted selection (MAS) programs. RF differences between sexes have been described in most fish species when constructing genetic maps [20,30,31,39,44-48], including Pleuronectiformes [40]. Recombination differences between families have also been reported, especially in humans and in domestic species [49,50], but few studies have been focused on this variation in fish and other aquaculture species [37,45,51,52]. In turbot, we observed a 1.6:1 female: male (F:M) RF ratio from a limited sample of common female/male marker pairs in the previous turbot map [25], but no significant RF differences between the two female maps constructed. Ruan *et al.*[2] also reported a higher F:M ratio (1.3:1) in this species.

**Figure 2:**
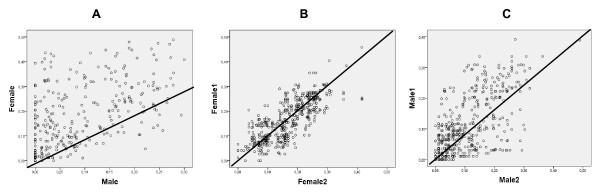
**Recombination frequency correlations between turbot maps.** (**A**) between sexes; **B**) among families within female; **C**) among families within male. Numbers 1 and 2 in the legend of axis in females (**B**) and males (**C**) represent whatever mother or father, respectively, of the reference mapping families.

In the present study, the availability of a large number of homogeneously distributed common markers in nine mapping families ( Additional file 2: Table S2) offered the opportunity for a detailed study on RF among families within sex, and between sexes. A global F:M ratio of 1.6:1 was observed (Figure 2A), thus corroborating our previous estimate [25]. The F:M ratio was largely variable among linkage groups ( Additional file 4: Table S3; Additional file 5: Figure S2), ranging between 0.93 at LG15, the only linkage group with higher male RF, and 23.22 at LG21, where a suggestive sex-determining QTL was previously reported [26]. These results support our previous observation related to the differential crossing-over patterns among turbot chromosomes when estimating gene-centromere distances [53]. RF differences among families within females (Figure 2B) were much lower than within males (Figure 2C). Accordingly, RF comparisons between females showed no significant differences, while they were significant between males at some cases. Inter-family RF differences have also been documented in other aquaculture species [37,52].

### Comparative mapping

**Figure 3:**
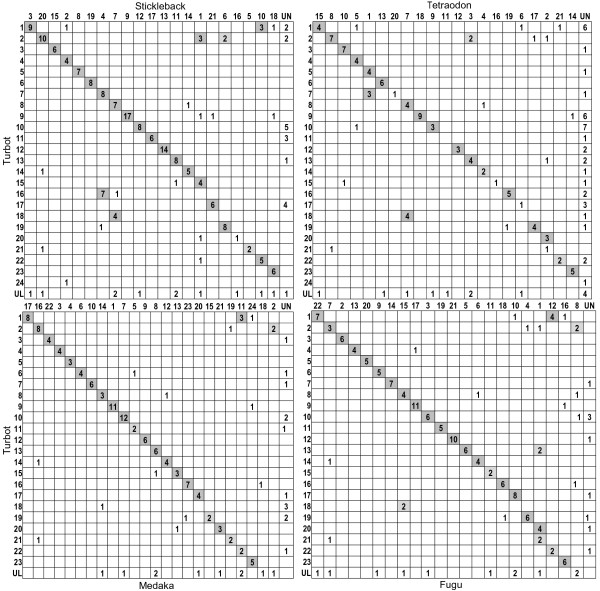
**Macrosynteny analysis between the turbot linkage map and model Acanthopterygii genomes.** In gray background syntenies with two or more significant hits. UL: unlinked markers in the turbot map; UN: unrandom genomic regions of the model fish species.

Similarity of turbot sequences against stickleback (Gac), *Tetraodon* (Tni), medaka (Ola), fugu (Tru) and zebrafish (Dre) genomes revealed large macrosyntenic patterns (Figure 3; Additional file 6: Figure S3 and Additional file 7: Figure S4). Total significant hits decreased from stickleback (~ 50%) to *Tetraodon*, fugu, medaka (~ 40%) and zebrafish (~20%) genomes (Table 2), mostly in agreement with the closer phylogenetic relationship of turbot to stickleback, *Tetraodon*, medaka and fugu (Acanthopterygii) than to zebrafish (Ostariophysi) [54]. The stickleback (Gasterosteiformes) genome was the most informative one in our study, despite that medaka (Beloniformes) has been found to be more closely related to turbot (Pleuronectiformes) using mitogenome data [55]. This may reflect phylogenetic discordances between mitochondrial and nuclear DNA markers, suggesting that both marker types should be combined to provide more consistent relationships among Acanthopterygii [56].

**Table 2:** Similarity of turbot EST-linked and anonymous sequences against model teleost genomes

**Model species**	**Gac**	**Tni**	**Ola**	**Tru**	**Dre**
Total hits E < 10^−5^ (%)	246 (54.4)	179 (39.4)	186 (41.0)	194 (42.7)	91 (20.5)
Unique hits^*a*^ (%)	210 (48.0)	151 (33.3)	146 (32.9)	162 (35.7)	69 (15.5)
Multiple hits^*b*^ (%)	36 (7.9)	28 (6.3)	38 (8.4)	32 (7.2)	22 (5.0)
EST-linked sequences (E)					
Unique hits^*a*^ (%)	121 (60.8)	98 (49.2)	88 (44.2)	93 (46.7)	50 (25.1)
Unique_L–L^*c*^ (%)	98 (49.2)	60 (30.2)	70 (35.2)	66 (33.2)	44 (22.1)
Multiple hits^*b*^ (%)	25 (12.6)	21 (10.6)	32 (16.1)	25 (12.6)	19 (9.6)
Anonymous sequences (A)					
Unique hits^*a*^ (%)	89 (36.3)	53 (26.6)	57 (22.4)	69 (27.1)	19 (7.8)
Unique_L–L^*c*^ (%)	78 (31.8)	40 (16.3)	53 (21.6)	66 (26.9)	17 (6.9)
Multiple hits^b^ (%)	11 (4.3)	7 (2.9)	7 (2.7)	7 (2.9)	3 (1.2)
Unique hits summary					
Mean size alignment bp	119.0	106.4	110.3	109.0	107.1
Mean size A-E^*d*^ bp	100.0–133.0	91.8–115.0	94.4–119.4	94.7–119.8	84.0–114.0
Maximum size A-E^*d*^ bp	257–504	252–401	215–422	222–438	181–295
Mean E-value	1.4E-07	4.2E-07	3.6E-07	4.0E-07	6.1E-07
Minimun E-value	1.0E-140	1.0E-116	1.0E-120	1.0E-119	2.0E-62
Identity % Mean	89.8	89.9	89.0	89.5	88.2
Identity % Range	79.4–100	80.4–100	79.9–96.7	79.4–100	80.3–96.2
Retained at E ≤ 10^−10^ (%)	182 (87%)	122 (81%)	110 (75%)	129 (80%)	50 (72%)

BLASTn matches of 454 turbot microsatellite and SNP flanking sequences (199 EST-linked to 255 anonymous ones) against the stickleback (Gac: *Gasterosteous aculeatus*), spotted green puperfish (Tni: *Tetraodon nigroviridis*), medaka (Ola: *Oryzias latypes*), fugu (Tru: *Takifugu rubripes*) and zebrafish (Dre: *Danio rerio*) genomes at E < 10^−5^ threshold, most of which retained at E ≤ 10^−10^. ^*a*^Unique or ^*b*^Multiple hits: turbot sequences yielding a single significant match or ≥2 significant matches, respectively, against model species genomes. ^*c*^Unique_L–L: linked markers at the turbot genetic map having unique significant matches with chromosome assignment on the model species genomes. ^*d*^A-E: size alignment figures in base pairs (bp) for Anonymous (A) to EST-linked (E) markers.

Gene-derived markers have demonstrated better performance than anonymous ones for comparative mapping [24,30]. Accordingly, more EST-linked than anonymous turbot markers matched against model genomes (Table 2). Most unique hits were included in the turbot map, thus being relevant to identify syntenic regions. Matches showed high average identity (~90%), the length similarity and identity increasing from zebrafish to stickleback and being higher for EST-linked than for anonymous markers (Table 2), as reported in teleosts [38,47].

### Macrosynteny between the turbot map and model teleost genomes

Mapping of 180 gene-derived markers to the turbot map has substantially improved previous comparative analysis based on anonymous loci [25], allowing the assessment of large syntenies between the turbot and the model fish genomes (Figure 3; Additional file 6: Figure S3 and Additional file 7: Figure S4). As expected, conserved syntenies (multiple significant hits regardless of their order) were higher against Acanthopterygii (20 to 25 conserved syntenies with four or more hits) than against zebrafish (only 14 small syntenies; Additional file 7: Figure S4) genomes. A remarkable one to one correspondence between the turbot linkage groups and the Acanthopterygii chromosomes was observed (Figure 3), in agreement with previous comparative mapping among model teleosts [21,57]. Synteny conservation was particularly extensive between the turbot and stickleback genomes (Table 2; Figure 3; Additional file 8: Table S4), aiding to establish a predicted location for most unlinked turbot markers from unique stickleback chromosomes. However, gene order appeared less conserved for most macrosyntenies ( Additional file 8: Table S4 and Additional file 9: Table S5) reflecting linkage mapping limitations and/or chromosome rearrangements over evolutionary time [22,30]. Collinearity appeared to be particularly conserved at microsyntenic scale ( Additional file 9: Table S5), as reported for other teleosts [30].

Comparative mapping provided additional support to the new LG4 and LG10, as well as to the fusion between LG8 and LG18, since they were syntenic to single chromosomes in all model Acanthopterygii (Figure 3; Additional file 6: Figure S3). By contrast, the independent syntenic relationship observed for LG16 and LG19 against model genomes (Figure 3) do not support the weak linkage signal observed between them. Further work will be required to establish the final merging on 22 linkage groups, both focusing on these linkage groups and on the smallest ones, particularly LG24. To achieve this goal, i) we are including new markers from Ruan *et al.*[2] in the turbot map; ii) we are performing two-color *in situ* hybridization with BAC probes associated to putative merging groups; and iii) we expect a draft of the turbot genome to be completed in the near future.

Comparative mapping also suggested fusion events in the stickleback (LG7 and LG16 merge into Gac4) and *Tetraodon* (LG5 and LG7 merge into Tni1) lineages as the most parsimonious hypothesis considering the ancestral n = 24 teleost karyotype [58] (Figure 3; Additional file 6: Figure S3). In accordance with the low rate of interchromosomal rearrangements in teleosts [57], only one turbot translocation between LG1 and LG22 was suggested from comparison with model species

Overall incidence of multiple matches against the five model teleost genomes was low, although higher from EST-linked (~10–16%) than from anonymous (<4%) markers (Table 2). This is likely related to the higher retention of duplication events on coding sequences along vertebrate evolution [59], and particularly, to the fish-specific (3R) whole genome duplication validated by comparative studies [19,21,24]. Close to 40% of the duplicated hits detected across model genomes in this study ( Additional file 10: Table S6) were congruent with the sets of orthologous and paralogous chromosomes identified between the *Tetraodon* and medaka genomes, which have been essential to reconstruct the vertebrate protokaryotype [57,60]. This information could capacitate to predict positions for unallocated duplicated genes on the turbot map.

### Anchoring the turbot map onto model and farm teleost genomes

Our study confirmed the findings of previous comparative mapping for farmed teleosts. Conserved synteny against closely related model genomes has been shown, either within Acanthopterygii (*Tetraodon*, medaka, fugu or stickleback), such as in the halibut, tilapia, Japanese flounder, European seabream or seabass [32,38,40,61], or within Ostariophysi (zebrafish), such as in the catfish or grass carp [23,30]. Anchoring of several farm fish maps against model teleost genomes is highly relevant to boost in aquaculture technologies, providing straightforward access to the gene content within specific syntenic regions from model species. For instance, linking the advances in the genomic analysis of commercially important pleuronectiform and perciform species will be possible using the stickleback as common anchoring genome given its informativeness for comparative mapping in turbot, tilapia, European seabream and seabass [32]. Also, the conservation of microsyntenies in the turbot map will be valuable to search for candidate genes of productive traits around QTL by data mining on the model fish genomes [33,34]. For this task, although the stickleback genome has demonstrated to be the most informative one, other model species within Acanthopterygii will also provide essential information, particularly medaka, a closely related model species to Pleuronectiformes [55].

## Conclusions

A gene-enriched turbot consensus map has been constructed with a marker density in the range of those described in farm fish species with large genomic resources. The availability of multiple reference families enabled us to obtain detailed data on RF between and within sexes. The higher evolutionary conservation of EST-linked markers allowed the detection of large macrosyntenic patterns with model fish species. This map provides essential information to identify genomic variation and candidate genes associated to productive traits for further application in MAS programs. The turbot map also provides useful landmarks for future turbot genome assembling and for evolutionary studies within pleuronectiforms and teleosts.

## Methods

### Mapping families

The haploid (HF) and diploid (DF) families from our previous studies [12,25], and seven additional families used for QTL identification (QF1-7) [26-28] were used to construct the new turbot map. DF was the main reference because of its higher marker density. HF was maintained in our analysis because a large set of anonymous markers had only been mapped in this family [25], but no new markers were added to this family. QF families were used when markers were non-informative in DF family. The seven QF families had been used for QTL screening on sex determination, growth and resistance to pathogens and thus, they were anchored by a common set of markers [26]. QF families were obtained from the genetic breeding programs of the companies Stolt Sea Farm SA and Insuiña SA, where a three-generation pedigree was available for all of them. Grandparents, parents and around 100 offspring (between 91 and 113) were analyzed in each QF family.

### Microsatellite and SNP markers

The following 463 markers (388 microsatellites and 75 SNP) were informative in the nine mapping families (HF, DF and QF1-7) ( Additional file 1: Table S1): i) 261 mostly anonymous microsatellites obtained from partial genomic libraries (Sma-USC codes) or RAPD markers (TUR codes) including: 248 from the previous map [25], 7 from Pardo *et al.*[62], 3 RAPD-derived from Liu *et al.*[63], and 3 novel markers characterized in the present work; and ii) 202 EST-linked markers, including 127 microsatellites: 43 from Bouza *et al.*[12], 75 from Navajas-Pérez *et al.*[13] (SmaUSC-E and Sma-E codes, respectively), and 9 from Chen *et al.* ([64]; SMAC codes); and 75 SNPs from Vera *et al.* ([7]; SmaSNP codes). For simplicity, hereinafter we shall refer to those microsatellites derived from enriched-genomic libraries or RAPD as anonymous microsatellites (despite some of them being annotated), and to the other group as EST-derived markers. Microsatellite and SNP genotyping was carried out on an ABI 3730 DNA Sequencer. Primers and PCR conditions for three new microsatellites were described for the first time in this work (Sma-USC286, Sma-USC287 and Sma-USC288; Additional file 1: Table S1). Chi-square tests were applied to check for deviations from Mendelian expectations (1:1, 1:2:1 and 1:1:1:1) at each locus and within each family analyzed.

### Map construction

#### Linkage analysis in mapping populations

A consensus genetic map was constructed using the nine reference family maps. Also, female and male genetic maps were constructed averaging *via* female and *via* male segregation, respectively, with the same diploid reference families. HF family was only used to build the female map. The software joinmap 3.0 [65] was used for map construction starting from all haploid and diploid mapping populations (HF, DF and QF1-7). The genotypes of the haploid gynogenetic progeny were coded as joinmap type HAP population, with linkage phase unknown. The segregation data from each parent of all diploid families were also coded in HAP configuration with known linkage phase to construct female and male maps. Diploid family data (DF and Q1-7) were coded as joinmap type CP population and analyzed within a known-phase model. Clustering and order of markers, as well as integrated linkage analysis to construct consensus, female and male maps were carried out using joinmap 3.0 with a LOD threshold > 3.0 for framework mapping, as previously reported [25]. The graphic maps were generated using mapchart 2.2 [66].

#### Comparison of recombination frequency (RF) between sexes and families

Only RF between framework markers (LOD > 3.0) was considered for comparisons. Common marker pairs were identified at each linkage group in the different mapping families to compare RF between families within each sex (i.e. segregating in the male or in the female) and between sexes. Comparisons between families within sex were performed both for all family pairs and globally using information of all families. Comparison between sexes was performed by averaging RF of common marker pairs across families within each sex. The mean and standard error of RF differences (between-families within males, between-families within females and between sexes) were obtained. Comparison was performed for the whole genetic map, but also for each linkage group. For these analyses, a minimum of 10 common marker pairs between the evaluated families was considered. The significance of RF differences for each pair of families was estimated using t-tests. Normality of RF distributions was checked using Kolmogorov-Smirnov tests. Non-parametric Mann–Whitney rank-order test was applied to evaluate the significance of RF differences between sexes.

### Comparative mapping

Given the high percentage of anonymous sequences used for linkage mapping in the turbot, NCBI-BLASTn was used to compare turbot containing-marker sequences against updated versions of model fish genomes downloaded from ftp://ftp.ensembl.org: *Tetraodon nigroviridis v.*8.61, *Takifugu rubripes* v.5, *Danio rerio* Zv9.6*, Oryzias latipes* v.1.61 and *Gasterosteus aculeatus v.*1.61. BLAST searching was performed by using a minimum alignment length of 40 bp with a score > 80 as recommended for EST mapping across species and two E-value thresholds (E ≤ 10^−10^ and E < 10^−5^) [26,32,67]. Turbot marker sequences were also used as queries for analysis against the stickleback cDNA database, as the most informative model genome in this study (see Results). We wrote a BioPerl BLAST parser to extract the desired hits including sequence similarity figures and genome location information for each model species.

## Competing interests

Some of the authors have received research funds in the past five years from private turbot companies related to genetic breeding programs. However, funding of the present manuscript has completely come from public institutions.

## Authors’ contributions

BGP was in charge of libraries construction to develop EST linked markers. MV was responsible of SNP development and genotyping in the reference families, and RH and RN did the same task with EST linked microsatellites. JAAD and AGT constructed EST databases and developed bioinformatic tools for marker development and comparative mapping. CF contributed to the revision and management of sequence data for marker development. MH was responsible of genetic map construction using joinmap. CB performed the comparative genomics analysis and together with PM wrote the manuscript. PM performed RF analyses and supervised and coordinated all the work. All authors read and approved the final manuscript.

## Supplementary Material

Additional file 1 **Table S1.** Characteristics of the genetic markers included in the turbot map. Click here for file

Additional file 2 **Table S2.** Informative markers used to construct the turbot consensus map. Click here for file

Additional file 3 **Figure S1.** Correspondence between the nine family maps used to construct the turbot consensus map. Click here for file

Additional file 4 **Table S3.** Number of markers and map length for each linkage group (LG) of turbot. Click here for file

Additional file 5 **Figure S2.** Consensus, female and male genetic maps of turbot. Click here for file

Additional file 6 **Figure S3.** Comparative mapping between the turbot map and the five model teleost genomes. Click here for file

Additional file 7 **Figure S4.** Oxford grid showing syntenies between the turbot linkage map and the zebrafish genome. Click here for file

Additional file 8 **Table S4.** Putative syntenic markers (210) between the turbot genetic map and the stickleback genome. Click here for file

Additional file 9 **Table S5.** Putative syntenic markers (60) between the turbot genetic map and four model Acanthopterygii genomes. Click here for file

Additional file 10 **Table S6.** Conservation of multiple similarity hits between the turbot and four model Acanthopterygii genomes. Click here for file

## References

[B1] FEAP Production and Price reports of member associations of the FEAP 2010 Belgium: Federation of European Aquaculture Producers, Liège

[B2] Ruan X Wang W Kong J Yu F Huang X Genetic linkage mapping of turbot (Scophthalmus maximus L.) using microsatellite markers and its application in QTL analysis Aquaculture 2010 308 89 100

[B3] Castro J Bouza C Presa P Pino-Querido A Riaza A Ferreiro I Sánchez L Martínez P Potential sources of error in parentage assessment of turbot (Scophthalmus maximus) using microsatellite loci Aquaculture 2004 242 119 135

[B4] Bouza C Sánchez L Martínez P Karyotypic characterization of turbot (Scophthalmus maximus) with conventional, fluorochrome, and restriction endonuclease banding techniques Mar Biol 1994 120 609 613

[B5] Cuñado N Terrones J Sánchez L Martínez P Santos JL Synaptonemal complex analysis in spermatocytes and oocytes of turbot, Scophthalmus maximus (Pisces, Scophthalmidae) Genome 2001 44 1143 1147 11768219 10.1139/g01-104

[B6] Pardo BG Fernández C Millán A Bouza C Vázquez-López A Vera M Alvarez-Dios JA Calaza M Gómez-Tato A Vázquez M Cabaleiro S Magariños B Lemos ML Leiro JM Martínez P Expressed sequence tags (ESTs) from immune tissues of turbot (Scophthalmus maximus) challenged with pathogens BMC Vet Res 2008 4 37 18817567 10.1186/1746-6148-4-37 PMC2569028

[B7] Vera M Alvarez-Dios JA Millán A Pardo BG Bouza C Hermida M Fernández C de la Herrán R Molina-Luzón MJ Martínez P Validation of single nucleotide polymorphism (SNP) markers from an immune Expressed Sequence Tag (EST) turbot, Scophthalmus maximus, database Aquaculture 2011 313 31 41

[B8] Millán A Gómez-Tato A Fernández C Pardo BG Álvarez-Dios JA Calaza M Bouza C Vázquez M Cabaleiro S Martínez P Design and performance of a turbot (Scophthalmus maximus) oligo-microarray based on ESTs from immune tissues Mar Biotechnol 2010 12 452 465 19844759 10.1007/s10126-009-9231-0

[B9] Millán A Gómez-Tato A Pardo BG Fernández C Bouza C Vera M Alvarez-Dios JA Cabaleiro S Lamas J Lemos ML Martínez P Gene expression profiles of the spleen, liver, and head kidney in turbot (Scophthalmus maximus) along the infection process with Aeromonas salmonicida using an immune-enriched oligo-microarray Mar Biotechnol 2011 10.1007/s10126-011-9374-7 21503602

[B10] Pardo BG Millán A Gómez-Tato A Fernández C Bouza C Alvarez-Dios A Cabaleiro S Lamas J Leiro JM Martínez P Gene expression profiles of spleen, liver and head kidney in turbot (Scophthalmus maximus) along the infection process with Philasterides dicentrarchi using an immune-enriched oligo-microarray Mar Biotechnol 2012 10.1007/s10126-012-9440-9 22367415

[B11] Cookson W Liang L Abecasis G Moffatt M Lathrop L Mapping complex disease traits with global gene expression Nat Rev Genet 2009 10 184 194 19223927 10.1038/nrg2537 PMC4550035

[B12] Bouza C Hermida M Millán A Vilas R Vera M Fernández C Pardo BG Martínez P Characterization of EST-derived microsatellites for gene mapping and evolutionary genomics in turbot Anim Genet 2008 39 666 670 18786152 10.1111/j.1365-2052.2008.01784.x

[B13] Navajas-Pérez R Robles F Molina-Luzón MJ de la Herrán R Álvarez-Dios JA Pardo BG Vera M Bouza C Martínez P Exploitation of an immune-related gene-enriched turbot (Scophthalmus maximus L.) expressed sequence tag (EST) database for microsatellite screening and validation Mol Ecol Res 2012 10.1111/j.1755-0998.2012.03126.x 22385869

[B14] Vilas R Bouza C Vera M Millán A Martínez P Variation in anonymous and EST-microsatellites suggests adaptive population divergence in turbot Mar Ecol Prog Ser 2010 420 231 239

[B15] Danzmann RG Gharbi K Liu ZJ Linkage mapping in aquaculture species Aquaculture Genome Technologies 2007 Oxford: Blackwell Publishing 139 167

[B16] Canario A Bargelloni L Volckaert F Houston RD Massault C Guiguen Y Genomics toolbox for farmed fish Rev Fish Sci 2008 16 1 13

[B17] Moen T Baranski M Sonesson AK Kjøglum S Confirmation and fine-mapping of a major QTL for resistance to infectious pancreatic necrosis in Atlantic salmon (Salmo salar): population-level associations between markers and trait BMC Genomics 2009 10 368 19664221 10.1186/1471-2164-10-368 PMC2728743

[B18] Shimoda N Knapik EW Ziniti J Sim C Yamada E Kaplan S Jackson D de Sauvage F Jacob H Fishman MC Zebrafish genetic map with 2000 microsatellite markers Genomics 1999 58 219 232 10373319 10.1006/geno.1999.5824

[B19] Jaillon O Aury JM Brunet F Petit JL Stange-Thomann N Mauceli E Bouneau L Fischer C Ozouf-Costaz C Bernot A Nicaud S Jaffe D Fisher S Lutfalla G Dossat C Segurens B Dasilva C Salanoubat M Levy M Boudet N Castellano S Anthouard V Jubin C Castelli V Katinka M Vacherie B Biémont C Skalli Z Cattolico L Poulain J De Berardinis V Cruaud C Duprat S Brottier P Coutanceau JP Gouzy J Parra G Lardier G Chapple C McKernan KJ McEwan P Bosak S Kellis M Volff JN Guigó R Zody MC Mesirov J Lindblad-Toh K Birren B Nusbaum C Kahn D Robinson-Rechavi M Laudet V Schachter V Quétier F Saurin W Scarpelli C Wincker P Lander ES Weissenbach J Roest Crollius H Genome duplication in the teleost fish Tetraodon nigroviridis reveals the early vertebrate proto-karyotype Nature 2004 431 946 957 15496914 10.1038/nature03025

[B20] Kai W Kikuchi K Fujita M Suetake H Fujiwara A Yoshiura Y Ototake M Venkatesh B Miyaki K Suzuki Y A genetic linkage map for the tiger pufferfish, Takifugu rubripes Genetics 2005 171 227 238 15972462 10.1534/genetics.105.042051 PMC1456513

[B21] Kai W Kikuchi K Tohari S Chew AK Tai A Fujiwara A Hosoya S Suetake H Naruse K Brenner S Suzuki Y Venkatesh B Integration of the genetic map and genome assembly of fugu facilitates insights into distinct features of genome evolution in Teleosts and mammals Genome Biol Evol 2011 3 424 442 21551351 10.1093/gbe/evr041 PMC5654407

[B22] Gross JB Protas M Conrad M Scheid PE Vidal O Jeffery WR Borowsky R Tabin CJ Synteny and candidate gene prediction using an anchored linkage map of Astyanax mexicanus Proc Natl Acad Sci U S A 2008 105 20106 20111 19104060 10.1073/pnas.0806238105 PMC2629299

[B23] Xia JH Liu F Zhu ZY Fu JJ Feng JB Yue GH A consensus linkage map of the grass carp (Ctenopharyngodon idella) based on microsatellites and SNPs BMC Genomics 2010 11 135 20181260 10.1186/1471-2164-11-135 PMC2838847

[B24] Naruse K Tanaka M Mita K Shima A Postlethwait J Mitani H A medaka gene map: The trace of ancestral vertebrate proto-chromosomes revealed by comparative gene mapping Genome Res 2009 14 820 828 15078856 10.1101/gr.2004004 PMC479108

[B25] Bouza C Hermida M Pardo BG Fernández C Castro J Fortes G Sánchez L Presa P Pérez M Sanjuán A Comesaña S Álvarez JA Calaza M Cal R Piferrer F Martínez P A microsatellite genetic map in the turbot (Scophthalmus maximus) Genetics 2007 177 2457 2467 18073440 10.1534/genetics.107.075416 PMC2219478

[B26] Martínez P Bouza C Hermida M Fernández J Toro MA Vera M Pardo BG Millán A Fernández C Vilas R Viñas A Sánchez L Felip A Piferrer F Ferreiro I Cabaleiro S Identification of the major sex-determining region of turbot (Scophthalmus maximus) Genetics 2009 183 1443 1452 19786621 10.1534/genetics.109.107979 PMC2787431

[B27] Sánchez-Molano E Cerna A Toro MA Bouza C Hermida M Pardo BG Cabaleiro S Fernández J Martínez P Detection of growth-related QTL in turbot (Scophthalmus maximus) BMC Genomics 2010 12 473 10.1186/1471-2164-12-473 PMC3195100 21958071

[B28] Rodríguez-Ramilo ST Toro MA Bouza C Hermida M Pardo BG Cabaleiro S Martínez P Fernández J QTL detection for Aeromonas salmonicida resistance related traits in turbot (Scophthalmus maximus) BMC Genomics 2011 12 541 22047500 10.1186/1471-2164-12-541 PMC3216323

[B29] Rodríguez-Ramilo ST Fernández J Toro MA Bouza C Hermida M Fernández C Pardo BG Cabaleiro S Martínez P Uncovering QTL for resistance and survival to Philasterides dicentrarchi in turbot (Scophthalmus maximus) Anim Genet 2012 10.1111/j.1365-2052.2012.02385.x 22690723

[B30] Kucuktas H Wang S Li P He C Xu P Sha Z Liu H Jiang Y Baoprasertkul P Somridhivej B Wang Y Abernathy J Guo X Liu L Muir W Liu Z Construction of genetic linkage maps and comparative genome analysis of catfish using gene-associated markers Genetics 2009 181 1649 1660 19171943 10.1534/genetics.108.098855 PMC2666527

[B31] Wang CM Bai ZY He XP Lin G Xia JH Sun F Lo LC Feng F Zhu ZY Yue GH A high resolution linkage map for comparative genome analysis and QTL fine mapping in Asian seabass, Lates calcarifer BMC Genomics 2011 12 174 21457569 10.1186/1471-2164-12-174 PMC3088568

[B32] Sarropoulou E Nousdili D Magoulas A Kotoulas G Linking the genomes of nonmodel Teleosts through comparative genomics Mar Biotechnol 2008 10 227 233 18297360 10.1007/s10126-007-9066-5

[B33] Loukovitis D Sarropoulou E Tsigenopoulos CS Batargias C Magoulas A Apostolidis AP Chatziplis D Kotoulas G Quantitative trait loci involved in sex determination and body growth in the gilthead sea bream (Sparus aurata L.) through targeted genome scan PLoS One 2011 6 e16599 21304996 10.1371/journal.pone.0016599 PMC3031595

[B34] Li J Boroevich KA Koop BF Davidson WS Comparative genomics identifies candidate genes for infectious salmon anemia (ISA) resistance in Atlantic salmon (Salmo salar) Mar Biotechnol 2011 13 232 241 20396924 10.1007/s10126-010-9284-0 PMC3084937

[B35] Greenwood AK Jones FC Chan YF Brady SD Absher DM Grimwood J Schmutz J Myers RM Kingsley DM Peichel CL The genetic basis of divergent pigment patterns in juvenile threespine sticklebacks Heredity 2011 107 155 166 21304547 10.1038/hdy.2011.1 PMC3136628

[B36] Dakin EE Avise JC Microsatellite null alleles in parentage analysis Heredity 2004 93 504 509 15292911 10.1038/sj.hdy.6800545

[B37] Hubert S Hedgecock D Linkage maps of microsatellite DNA markers for the pacific oyster Crassostrea gigas Genetics 2004 168 351 362 15454548 10.1534/genetics.104.027342 PMC1448102

[B38] Chistiakov DA Tsigenopoulos CS Lagnel J Guo YM Hellemans B Haley CS Volckaert FA Kotoulas G A combined AFLP and microsatellite linkage map and pilot comparative genomic analysis of European sea bass Dicentrarchus labrax L Anim Genet 2008 39 623 634 18828863 10.1111/j.1365-2052.2008.01786.x

[B39] Rexroad CE Palti Y Gahr SA Vallejo RL A second generation genetic map of rainbow trout (Oncorhynchus mykiss) BMC Genet 2008 9 74 19019240 10.1186/1471-2156-9-74 PMC2605456

[B40] Castaño-Sánchez C Fuji K Ozaki A Hasegawa O Sakamoto T Morishima K Nakayama I Fujiwara A Masaoka T Okamoto H Hayashida K Tagami M Kawai J Hayashizaki Y Okamoto N A second generation genetic linkage map of Japanese flounder (Paralichthys olivaceus) BMC Genomics 2010 11 554 20937088 10.1186/1471-2164-11-554 PMC3091703

[B41] Dekkers JCM Hospital F The use of molecular genetics in the improvement of agricultural populations Nat Rev Genet 2002 3 22 32 11823788 10.1038/nrg701

[B42] Hardei DC Hubert PDN Genome-size evolution in fishes Can J Fish Aquat Sci 2004 61 1636 1646

[B43] Brown TA Genomes 1999 Oxford: BIOS Scientific Publishers Ltd

[B44] Moen T Hayes B Baranski M Berg PR Kjøglum S Koop BF Davidson WS Omholt SW Lien S A linkage map of the Atlantic salmon (Salmo salar) based on EST-derived SNP marker BMC Genomics 2008 9 223 18482444 10.1186/1471-2164-9-223 PMC2405805

[B45] Woram RA McGowan C Stout JA Gharbi K Ferguson MM Hoyheim B Davidson EA Davidson WS Rexroad C Danzmann RG A genetic linkage map for Arctic char (Salvelinus alpinus): evidence for higher recombination rates and segregation distortion in hybrid versus pure strain mapping parents Genome 2004 47 304 315 15060583 10.1139/g03-127

[B46] Lee BY Lee WJ Streelman JT Carleton KL Howe AE Hulata G Slettan A Stern JE Terai Y Kocher TD A second-generation genetic linkage map of tilapia (Oreochromis spp.) Genetics 2005 170 237 244 15716505 10.1534/genetics.104.035022 PMC1449707

[B47] Franch R Louro B Tsalavouta M Chatziplis D Tsigenopoulos CS Sarropoulou E Antonello J Magoulas A Mylonas CC Babbucci M Patarnello T Power DM Kotoulas G Bargelloni L A genetic linkage map of the hermaphrodite teleost fish Sparus aurata L Genetics 2006 174 851 861 16951080 10.1534/genetics.106.059014 PMC1602104

[B48] Gharbi K Gautier A Danzmann RG Gharbi S Sakamoto T Høyheim B Taggart JB Cairney M Powell R Krieg F Okamoto N Ferguson MM Holm LE Guyomard R A linkage map for brown trout (Salmo trutta): chromosome homeologies and comparative genome organization with other salmonid fish Genetics 2006 172 2405 2419 16452148 10.1534/genetics.105.048330 PMC1456399

[B49] Coop G Wen XQ Ober C Pritchard JK Przeworski M High-resolution mapping of crossovers reveals extensive variation in fine-scale recombination patterns among humans Science 2008 319 1395 1398 18239090 10.1126/science.1151851

[B50] Ma JW Iannucelli N Duan YY Huang WB Guo BL Riquet J Huang L Milan D Recombinational landscape of porcine X chromosome and individual variation in female meiotic recombination associated with haplotypes of Chinese pigs BMC Genomics 2010 11 159 20211033 10.1186/1471-2164-11-159 PMC2850356

[B51] Allendorf FW Seeb KE Knudsen KL Thorgaard GH Leary RF Gene-centromere mapping of 25 loci in rainbow trout J Hered 1986 77 307 321

[B52] Sekino M Hara M Linkage maps for the Pacific abalone (genus Haliotis) based on microsatellite DNA markers Genetics 2007 175 945 958 17151239 10.1534/genetics.106.065839 PMC1800609

[B53] Martínez P Hermida M Pardo BG Fernández C Castro J Cal RM Alvarez-Dios JA Gómez-Tato A Bouza C Centromere-linkage in the turbot (Scophthalmus maximus) through half-tetrad analysis in diploid meiogynogenetics Aquaculture 2008 280 81 88

[B54] Miya M Takeshima H Endo H Ishiguro NB Inoue JG Mukai T Satoh TP Yamaguchi M Kawaguchi A Mabuchi K Shirai SM Nishida M Major patterns of higher teleostean phylogenies: a new perspective based on 100 complete mitochondrial DNA sequences Mol Phyl Evol 2003 26 121 138 10.1016/s1055-7903(02)00332-9 12470944

[B55] Mabuchi K Miya M Azuma Y Nishida M Independent evolution of the specialized pharyngeal jaw apparatus in Cichlid and Labrid fishes BMC Evol Biol 2007 7 10 17263894 10.1186/1471-2148-7-10 PMC1797158

[B56] Li C Lu G Orti G Optimal data partitioning and a test case for ray-finned fishes (Actinopterygii) based on ten nuclear loci Syst Biol 2008 57 519 539 18622808 10.1080/10635150802206883

[B57] Kasahara M Naruse K Sasaki S Nakatani Y Qu W Ahsan B Yamada T Nagayasu Y Doi K Kasai Y Jindo T Kobayashi D Shimada A Toyoda A Kuroki Y Fujiyama A Sasaki T Shimizu A Asakawa S Shimizu N Hashimoto S Yang J Lee Y Matsushima K Sugano S Sakaizumi M Narita T Ohishi K Haga S Ohta F Nomoto H Nogata K Morishita T Endo T Shin-I T Takeda H Morishita S Kohara Y The medaka draft genome and insights into vertebrate genome evolution Nature 2007 447 714 719 17554307 10.1038/nature05846

[B58] Ohno S Enormous diversity in genome sizes of fish as a reflection of nature’s extensive experiments with gene duplication Trans Am Fish Soc 1970 99 120 130

[B59] Hedges SB Kumar S Genomics vertebrate genomes compared Science 2002 297 1283 1285 12193771 10.1126/science.1076231

[B60] Kohn M Högel J Vogel W Minich P Kehrer-Sawatzki H Graves JA Hameister H Reconstruction of a 450-My-old ancestral vertebrate protokaryotype Trends Genet 2006 22 203 210 16517001 10.1016/j.tig.2006.02.008

[B61] Reid DP Smith CA Rommens M Blanchard B Martin-Robichaud D Reith M A genetic linkage map of Atlantic halibut (Hippoglossus hippoglossus L.) Genetics 2007 177 1193 1205 17720928 10.1534/genetics.107.075374 PMC2034623

[B62] Pardo BG Fernández C Hermida M Vázquez A Pérez M Presa P Calaza M Alvarez-Dios JA Comesaña AS Raposo-Guillán J Bouza C Martínez P Development and characterization of 248 novel microsatellite markers in turbot (Scophthalmus maximus) Genome 2007 50 329 332 17502907 10.1139/g06-154

[B63] Liu YG Liu LX Lei ZW Gao AY Li BF Identification of polymorphic microsatellite markers from RAPD product in turbot (Scophthalmus maximus) and a test of cross-species amplification Mol Ecol Notes 2006 6 867 869

[B64] Chen SL Ma HY Jiang Y Liao XL Meng L Isolation and characterization of polymorphic microsatellite loci from an EST library of turbot (Scophthalmus maximus) and cross-species amplification Mol Ecol Notes 2007 7 848 850

[B65] Van Ooijen JW Voorrips RE JoinMap® version 3.0: software for the calculation of genetic linkage maps 2001 Wageningen: Plant Research International

[B66] Voorrips RE MapChart: Software for the graphical presentation of linkage maps and QTLs J Hered 2002 93 77 78 12011185 10.1093/jhered/93.1.77

[B67] Stemshorn KC Nolte AW Tautz D A genetic map of Cottus gobio (Pisces, Teleostei) based on microsatellites can be linked to the physical map of Tetraodon nigroviridis J Evol Biol 2005 18 1619 1624 16313473 10.1111/j.1420-9101.2005.00929.x

