# Supplementary material for: An Expressed Sequence Tag (EST)-enriched genetic map of turbot (Scophthalmus maximus): a useful framework for comparative genomics across model and farmed teleosts
Source: BMC Genet. 2012 Jul 2;13:54. doi: 10.1186/1471-2156-13-54 (PMC3464660; doi:10.1186/1471-2156-13-54)
Supplement: Additional file 1 — Table S1. Characteristics of the genetic markers included in the turbot map. [file 1471-2156-13-54-S1.pdf]

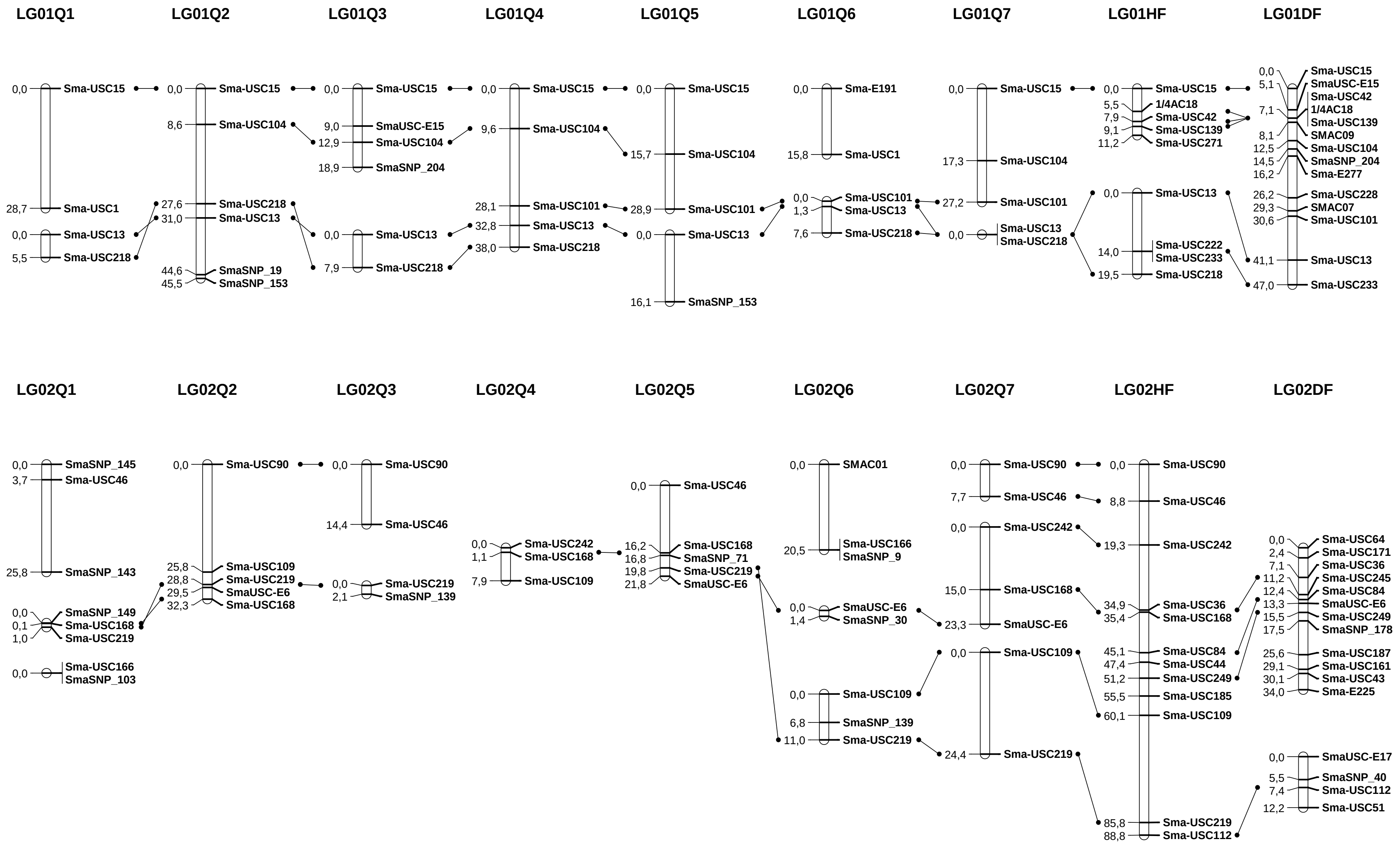

LG01Q1
0,0 Sma-USC15
28,7 Sma-USC1
0,0 Sma-USC13
5,5 Sma-USC218
LG01Q2
0,0 Sma-USC15
8,6 Sma-USC104
27,6 Sma-USC218
31,0 Sma-USC13
44,6 SmaSNP_19
45,5 SmaSNP_153
LG01Q3
0,0 Sma-USC15
9,0 SmaUSC-E15
12,9 Sma-USC104
18,9 SmaSNP_204
0,0 Sma-USC13
7,9 Sma-USC218
LG01Q4
0,0 Sma-USC15
9,6 Sma-USC104
28,1 Sma-USC101
32,8 Sma-USC13
38,0 Sma-USC218
LG01Q5
0,0 Sma-USC15
15,7 Sma-USC104
28,9 Sma-USC101
0,0 Sma-USC13
16,1 SmaSNP_153
LG01Q6
0,0 Sma-E191
15,8 Sma-USC1
0,0 Sma-USC101
1,3 Sma-USC13
7,6 Sma-USC218
LG01Q7
0,0 Sma-USC15
17,3 Sma-USC104
27,2 Sma-USC101
0,0 Sma-USC13
Sma-USC218
LG01HF
0,0 Sma-USC15
5,5 1/4AC18
7,9 Sma-USC42
9,1 Sma-USC139
11,2 Sma-USC271
0,0 Sma-USC13
14,0 Sma-USC222
Sma-USC233
19,5 Sma-USC218
LG01DF
0,0 Sma-USC15
5,1 SmaUSC-E15
Sma-USC42
7,1 1/4AC18
Sma-USC139
8,1 SMAC09
12,5 Sma-USC104
14,5 SmaSNP_204
16,2 Sma-E277
26,2 Sma-USC228
29,3 SMAC07
30,6 Sma-USC101
41,1 Sma-USC13
47,0 Sma-USC233
LG02Q1
0,0 SmaSNP_145
3,7 Sma-USC46
25,8 SmaSNP_143
0,0 SmaSNP_149
0,1 Sma-USC168
1,0 Sma-USC219
0,0 Sma-USC166
SmaSNP_103
LG02Q2
0,0 Sma-USC90
25,8 Sma-USC109
28,8 Sma-USC219
29,5 SmaUSC-E6
32,3 Sma-USC168
LG02Q3
0,0 Sma-USC90
14,4 Sma-USC46
0,0 Sma-USC219
2,1 SmaSNP_139
LG02Q4
0,0 Sma-USC242
1,1 Sma-USC168
7,9 Sma-USC109
LG02Q5
0,0 Sma-USC46
16,2 Sma-USC168
16,8 SmaSNP_71
19,8 Sma-USC219
21,8 SmaUSC-E6
LG02Q6
0,0 SMAC01
20,5 Sma-USC166
SmaSNP_9
0,0 SmaUSC-E6
1,4 SmaSNP_30
0,0 Sma-USC109
6,8 SmaSNP_139
11,0 Sma-USC219
LG02Q7
0,0 Sma-USC90
7,7 Sma-USC46
0,0 Sma-USC242
15,0 Sma-USC168
23,3 SmaUSC-E6
0,0 Sma-USC109
24,4 Sma-USC219
LG02HF
0,0 Sma-USC90
8,8 Sma-USC46
19,3 Sma-USC242
34,9 Sma-USC36
35,4 Sma-USC168
45,1 Sma-USC84
47,4 Sma-USC44
51,2 Sma-USC249
55,5 Sma-USC185
60,1 Sma-USC109
85,8 Sma-USC219
88,8 Sma-USC112
LG02DF
0,0 Sma-USC64
2,4 Sma-USC171
7,1 Sma-USC36
11,2 Sma-USC245
12,4 Sma-USC84
13,3 SmaUSC-E6
15,5 Sma-USC249
17,5 SmaSNP_178
25,6 Sma-USC187
29,1 Sma-USC161
30,1 Sma-USC43
34,0 Sma-E225
0,0 SmaUSC-E17
5,5 SmaSNP_40
7,4 Sma-USC112
12,2 Sma-USC51

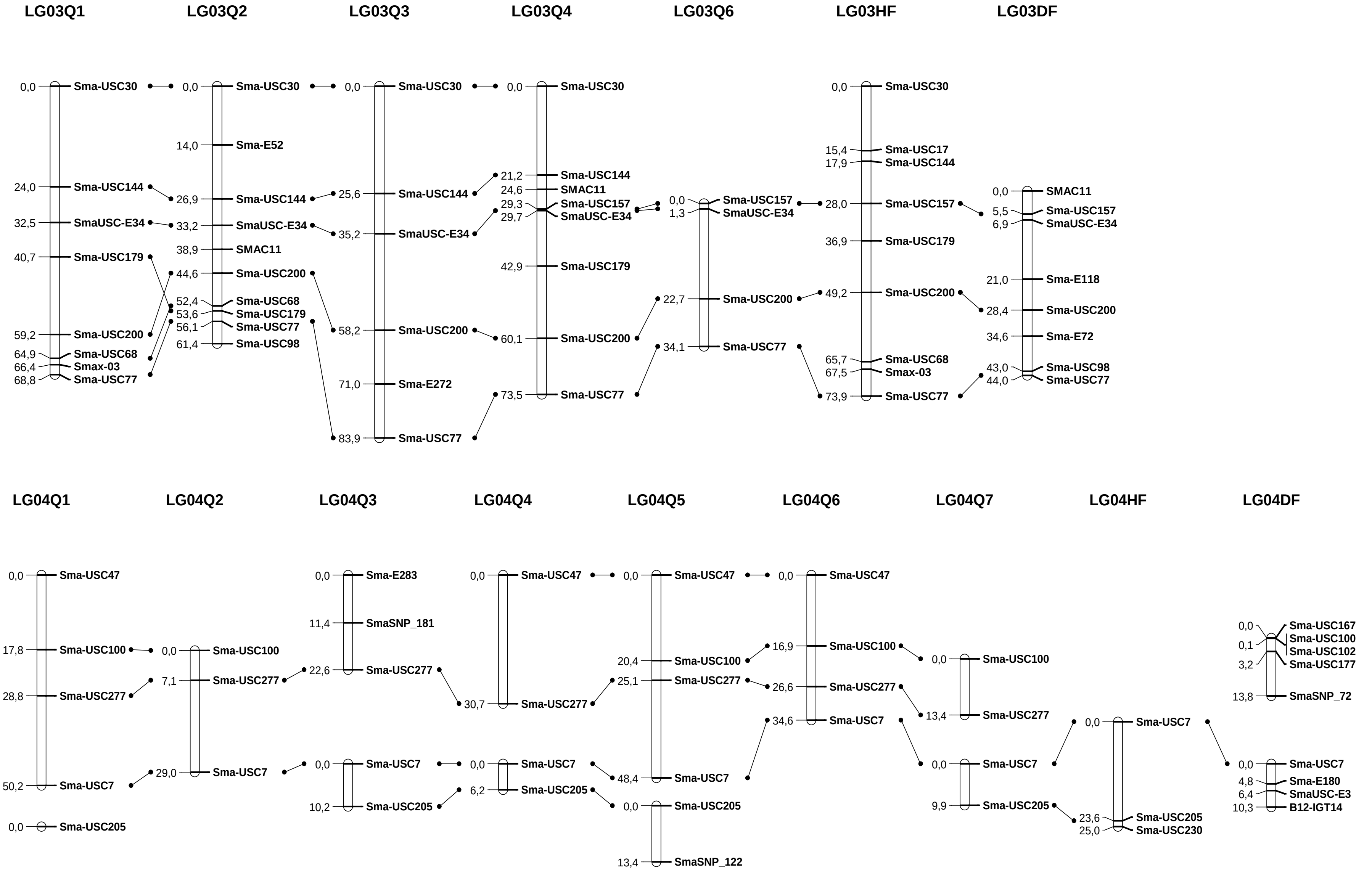
LG03Q1
LG03Q2
LG03Q3
LG03Q4
LG03Q6
LG03HF
LG03DF
0,0 Sma-USC30
24,0 Sma-USC144
32,5 SmaUSC-E34
40,7 Sma-USC179
59,2 Sma-USC200
64,9 Sma-USC68
66,4 Smax-03
68,8 Sma-USC77
0,0 Sma-USC30
14,0 Sma-E52
26,9 Sma-USC144
33,2 SmaUSC-E34
38,9 SMAC11
44,6 Sma-USC200
52,4 Sma-USC68
53,6 Sma-USC179
56,1 Sma-USC77
61,4 Sma-USC98
0,0 Sma-USC30
25,6 Sma-USC144
35,2 SmaUSC-E34
58,2 Sma-USC200
71,0 Sma-E272
83,9 Sma-USC77
0,0 Sma-USC30
21,2 Sma-USC144
24,6 SMAC11
29,3 Sma-USC157
29,7 SmaUSC-E34
42,9 Sma-USC179
60,1 Sma-USC200
73,5 Sma-USC77
0,0 Sma-USC157
1,3 SmaUSC-E34
22,7 Sma-USC200
34,1 Sma-USC77
0,0 Sma-USC30
15,4 Sma-USC17
17,9 Sma-USC144
28,0 Sma-USC157
36,9 Sma-USC179
49,2 Sma-USC200
65,7 Sma-USC68
67,5 Smax-03
73,9 Sma-USC77
0,0 SMAC11
5,5 Sma-USC157
6,9 SmaUSC-E34
21,0 Sma-E118
28,4 Sma-USC200
34,6 Sma-E72
43,0 Sma-USC98
44,0 Sma-USC77
LG04Q1
LG04Q2
LG04Q3
LG04Q4
LG04Q5
LG04Q6
LG04Q7
LG04HF
LG04DF
0,0 Sma-USC47
17,8 Sma-USC100
28,8 Sma-USC277
50,2 Sma-USC7
0,0 Sma-USC205
0,0 Sma-USC100
7,1 Sma-USC277
29,0 Sma-USC7
0,0 Sma-E283
11,4 SmaSNP_181
22,6 Sma-USC277
0,0 Sma-USC7
10,2 Sma-USC205
0,0 Sma-USC47
30,7 Sma-USC277
0,0 Sma-USC7
6,2 Sma-USC205
0,0 Sma-USC47
20,4 Sma-USC100
25,1 Sma-USC277
48,4 Sma-USC7
0,0 Sma-USC205
13,4 SmaSNP_122
0,0 Sma-USC47
16,9 Sma-USC100
26,6 Sma-USC277
34,6 Sma-USC7
0,0 Sma-USC100
13,4 Sma-USC277
0,0 Sma-USC7
9,9 Sma-USC205
0,0 Sma-USC7
23,6 Sma-USC205
25,0 Sma-USC230
0,0 Sma-USC167
0,1 Sma-USC100
Sma-USC102
3,2 Sma-USC177
13,8 SmaSNP_72
0,0 Sma-USC7
4,8 Sma-E180
6,4 SmaUSC-E3
10,3 B12-IGT14

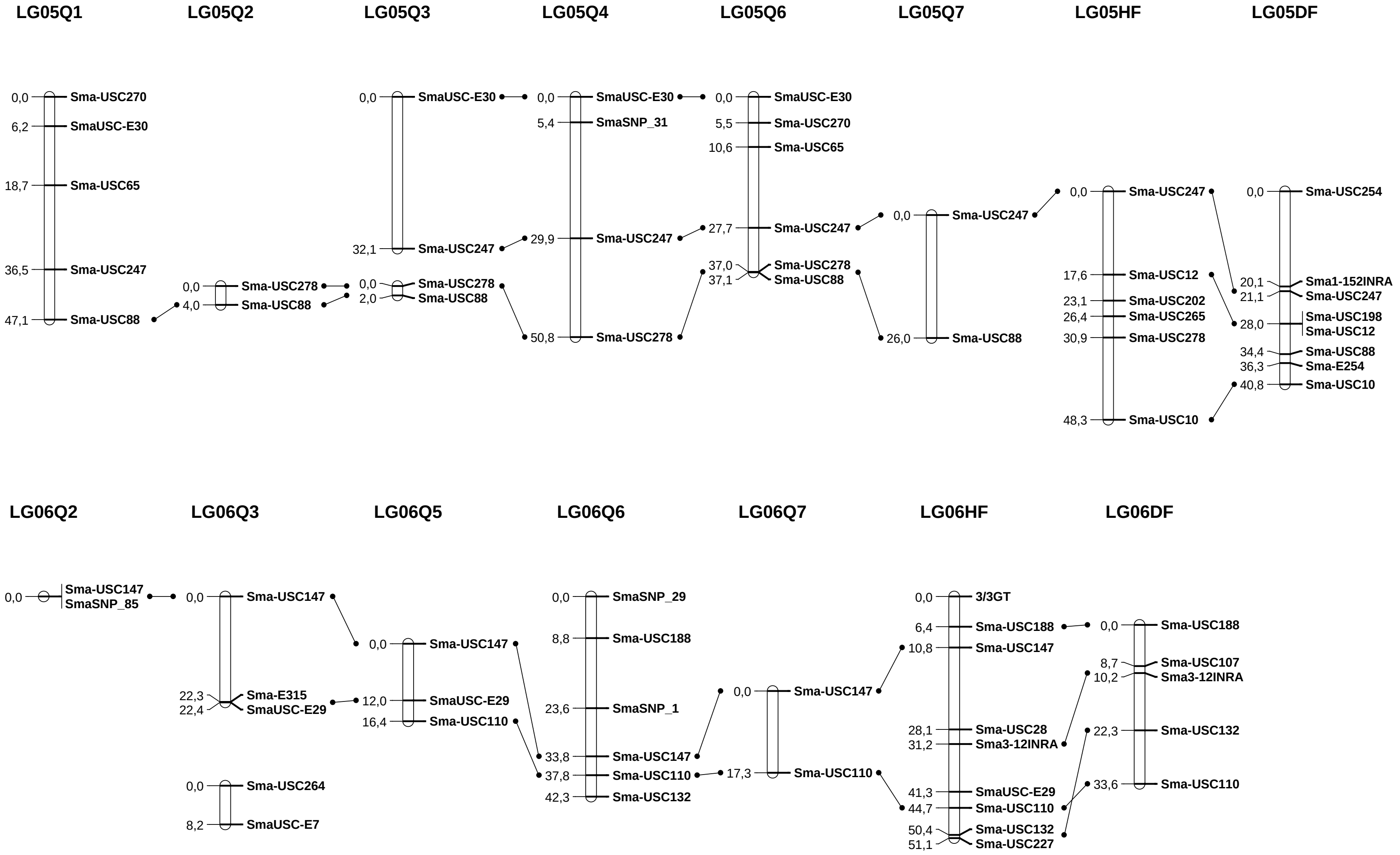
LG05Q1
0,0 Sma-USC270
6,2 SmaUSC-E30
18,7 Sma-USC65
36,5 Sma-USC247
47,1 Sma-USC88
LG05Q2
0,0 Sma-USC278
4,0 Sma-USC88
LG05Q3
0,0 SmaUSC-E30
32,1 Sma-USC247
0,0 Sma-USC278
2,0 Sma-USC88
LG05Q4
0,0 SmaUSC-E30
5,4 SmaSNP_31
29,9 Sma-USC247
50,8 Sma-USC278
LG05Q6
0,0 SmaUSC-E30
5,5 Sma-USC270
10,6 Sma-USC65
27,7 Sma-USC247
37,0 Sma-USC278
37,1 Sma-USC88
LG05Q7
0,0 Sma-USC247
26,0 Sma-USC88
LG05HF
0,0 Sma-USC247
17,6 Sma-USC12
23,1 Sma-USC202
26,4 Sma-USC265
30,9 Sma-USC278
48,3 Sma-USC10
LG05DF
0,0 Sma-USC254
20,1 Sma1-152INRA
21,1 Sma-USC247
28,0 Sma-USC198
Sma-USC12
34,4 Sma-USC88
36,3 Sma-E254
40,8 Sma-USC10
LG06Q2
0,0 Sma-USC147
SmaSNP_85
LG06Q3
0,0 Sma-USC147
22,3 Sma-E315
22,4 SmaUSC-E29
0,0 Sma-USC264
8,2 SmaUSC-E7
LG06Q5
0,0 Sma-USC147
12,0 SmaUSC-E29
16,4 Sma-USC110
LG06Q6
0,0 SmaSNP_29
8,8 Sma-USC188
23,6 SmaSNP_1
33,8 Sma-USC147
37,8 Sma-USC110
42,3 Sma-USC132
LG06Q7
0,0 Sma-USC147
17,3 Sma-USC110
LG06HF
0,0 3/3GT
6,4 Sma-USC188
10,8 Sma-USC147
28,1 Sma-USC28
31,2 Sma3-12INRA
41,3 SmaUSC-E29
44,7 Sma-USC110
50,4 Sma-USC132
51,1 Sma-USC227
LG06DF
0,0 Sma-USC188
8,7 Sma-USC107
10,2 Sma3-12INRA
22,3 Sma-USC132
33,6 Sma-USC110

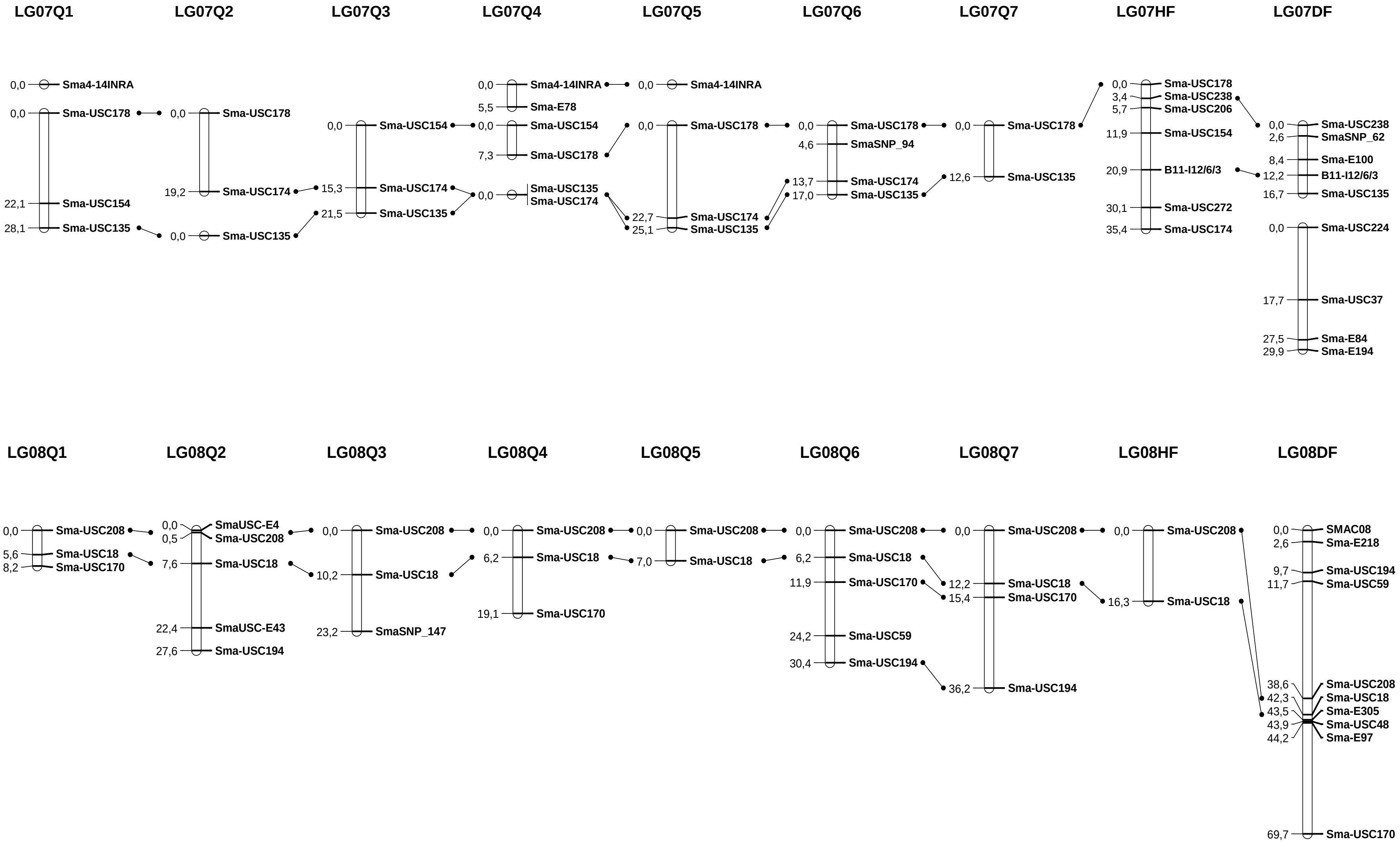
LG07Q1
0,0 Sma4-14INRA
0,0 Sma-USC178
22,1 Sma-USC154
28,1 Sma-USC135
LG07Q2
0,0 Sma-USC178
19,2 Sma-USC174
0,0 Sma-USC135
LG07Q3
0,0 Sma-USC154
15,3 Sma-USC174
21,5 Sma-USC135
LG07Q4
0,0 Sma4-14INRA
5,5 Sma-E78
0,0 Sma-USC154
7,3 Sma-USC178
0,0 Sma-USC135
Sma-USC174
LG07Q5
0,0 Sma4-14INRA
0,0 Sma-USC178
22,7 Sma-USC174
25,1 Sma-USC135
LG07Q6
0,0 Sma-USC178
4,6 SmaSNP_94
13,7 Sma-USC174
17,0 Sma-USC135
LG07Q7
0,0 Sma-USC178
12,6 Sma-USC135
LG07HF
0,0 Sma-USC178
3,4 Sma-USC238
5,7 Sma-USC206
11,9 Sma-USC154
20,9 B11-I12/6/3
30,1 Sma-USC272
35,4 Sma-USC174
LG07DF
0,0 Sma-USC238
2,6 SmaSNP_62
8,4 Sma-E100
12,2 B11-I12/6/3
16,7 Sma-USC135
0,0 Sma-USC224
17,7 Sma-USC37
27,5 Sma-E84
29,9 Sma-E194
LG08Q1
0,0 Sma-USC208
5,6 Sma-USC18
8,2 Sma-USC170
LG08Q2
0,0 SmaUSC-E4
0,5 Sma-USC208
7,6 Sma-USC18
22,4 SmaUSC-E43
27,6 Sma-USC194
LG08Q3
0,0 Sma-USC208
10,2 Sma-USC18
23,2 SmaSNP_147
LG08Q4
0,0 Sma-USC208
6,2 Sma-USC18
19,1 Sma-USC170
LG08Q5
0,0 Sma-USC208
7,0 Sma-USC18
LG08Q6
0,0 Sma-USC208
6,2 Sma-USC18
11,9 Sma-USC170
24,2 Sma-USC59
30,4 Sma-USC194
LG08Q7
0,0 Sma-USC208
12,2 Sma-USC18
15,4 Sma-USC170
36,2 Sma-USC194
LG08HF
0,0 Sma-USC208
16,3 Sma-USC18
LG08DF
0,0 SMAC08
2,6 Sma-E218
9,7 Sma-USC194
11,7 Sma-USC59
38,6 Sma-USC208
42,3 Sma-USC18
43,5 Sma-E305
43,9 Sma-USC48
44,2 Sma-E97
69,7 Sma-USC170

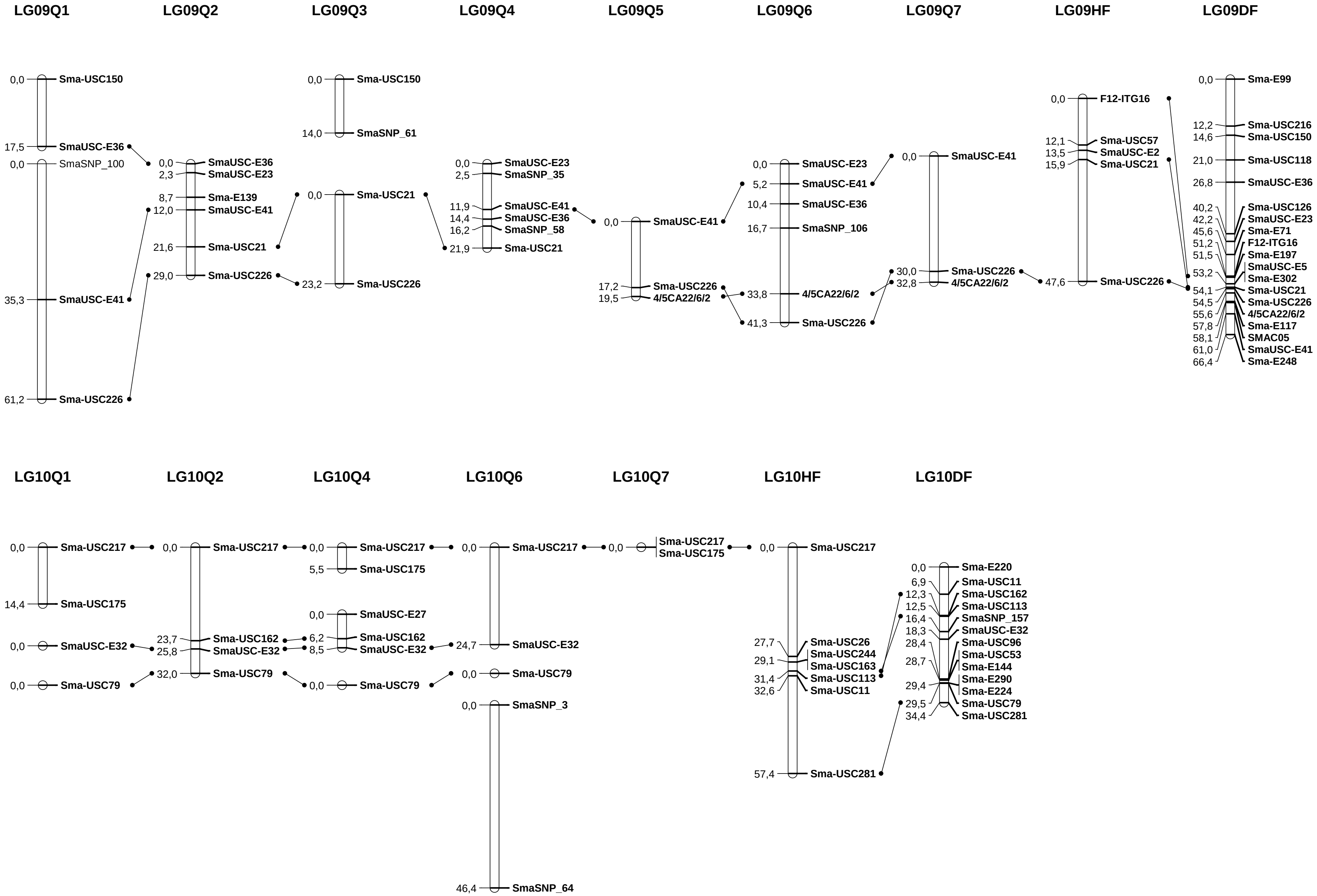

LG09Q1
LG09Q2
LG09Q3
LG09Q4
LG09Q5
LG09Q6
LG09Q7
LG09HF
LG09DF
LG10Q1
LG10Q2
LG10Q4
LG10Q6
LG10Q7
LG10HF
LG10DF

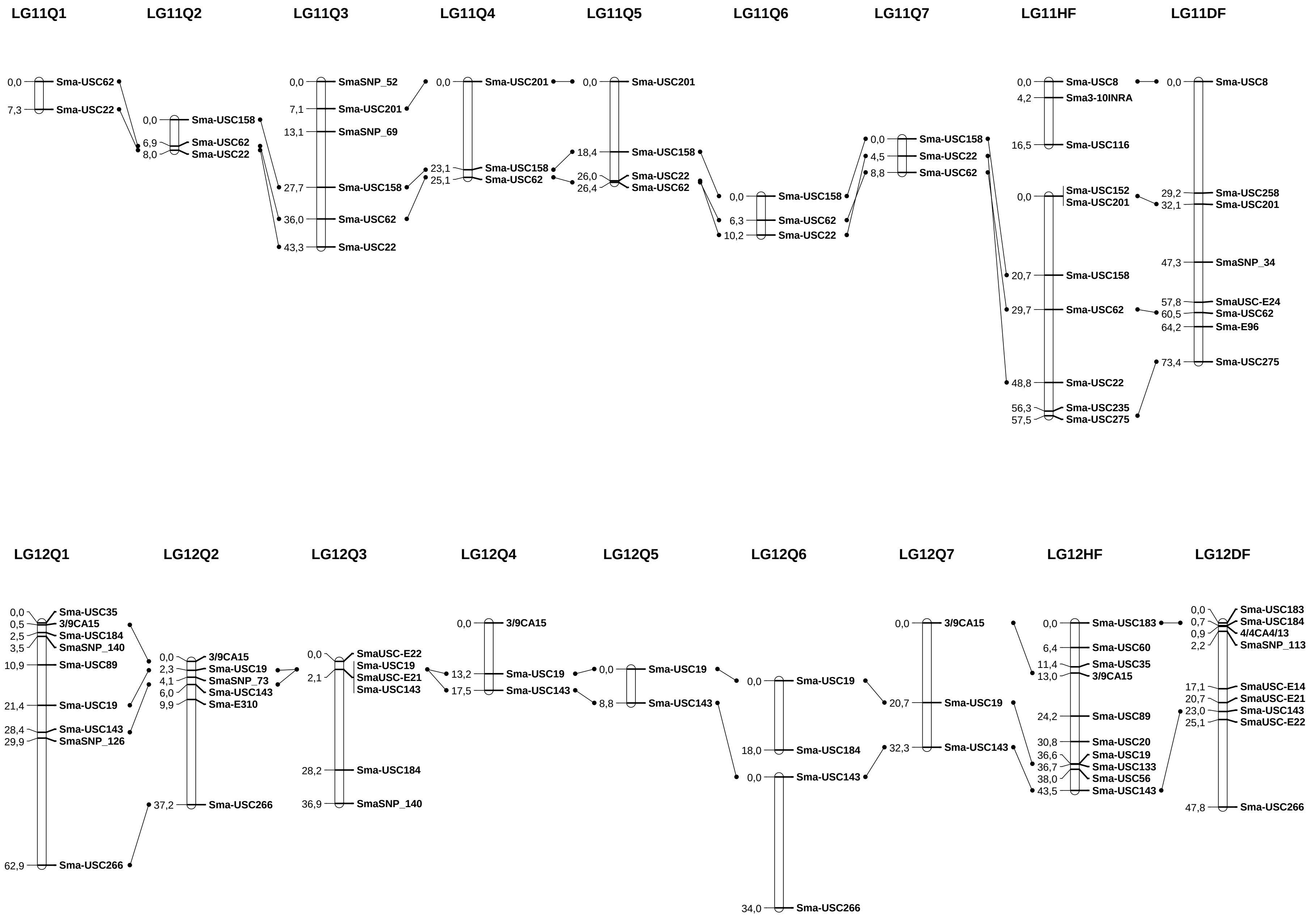
LG11Q1
0,0 Sma-USC62
7,3 Sma-USC22
LG11Q2
0,0 Sma-USC158
6,9 Sma-USC62
8,0 Sma-USC22
LG11Q3
0,0 SmaSNP_52
7,1 Sma-USC201
13,1 SmaSNP_69
27,7 Sma-USC158
36,0 Sma-USC62
43,3 Sma-USC22
LG11Q4
0,0 Sma-USC201
23,1 Sma-USC158
25,1 Sma-USC62
LG11Q5
0,0 Sma-USC201
18,4 Sma-USC158
26,0 Sma-USC22
26,4 Sma-USC62
LG11Q6
0,0 Sma-USC158
6,3 Sma-USC62
10,2 Sma-USC22
LG11Q7
0,0 Sma-USC158
4,5 Sma-USC22
8,8 Sma-USC62
LG11HF
0,0 Sma-USC8
4,2 Sma3-10INRA
16,5 Sma-USC116
0,0 Sma-USC152
Sma-USC201
20,7 Sma-USC158
29,7 Sma-USC62
48,8 Sma-USC22
56,3 Sma-USC235
57,5 Sma-USC275
LG11DF
0,0 Sma-USC8
29,2 Sma-USC258
32,1 Sma-USC201
47,3 SmaSNP_34
57,8 SmaUSC-E24
60,5 Sma-USC62
64,2 Sma-E96
73,4 Sma-USC275
LG12Q1
0,0 Sma-USC35
0,5 3/9CA15
2,5 Sma-USC184
3,5 SmaSNP_140
10,9 Sma-USC89
21,4 Sma-USC19
28,4 Sma-USC143
29,9 SmaSNP_126
62,9 Sma-USC266
LG12Q2
0,0 3/9CA15
2,3 Sma-USC19
4,1 SmaSNP_73
6,0 Sma-USC143
9,9 Sma-E310
37,2 Sma-USC266
LG12Q3
0,0 SmaUSC-E22
2,1 Sma-USC19
SmaUSC-E21
Sma-USC143
28,2 Sma-USC184
36,9 SmaSNP_140
LG12Q4
0,0 3/9CA15
13,2 Sma-USC19
17,5 Sma-USC143
LG12Q5
0,0 Sma-USC19
8,8 Sma-USC143
LG12Q6
0,0 Sma-USC19
18,0 Sma-USC184
0,0 Sma-USC143
34,0 Sma-USC266
LG12Q7
0,0 3/9CA15
20,7 Sma-USC19
32,3 Sma-USC143
LG12HF
0,0 Sma-USC183
6,4 Sma-USC60
11,4 Sma-USC35
13,0 3/9CA15
24,2 Sma-USC89
30,8 Sma-USC20
36,6 Sma-USC19
36,7 Sma-USC133
38,0 Sma-USC56
43,5 Sma-USC143
LG12DF
0,0 Sma-USC183
0,7 Sma-USC184
0,9 4/4CA4/13
2,2 SmaSNP_113
17,1 SmaUSC-E14
20,7 SmaUSC-E21
23,0 Sma-USC143
25,1 SmaUSC-E22
47,8 Sma-USC266

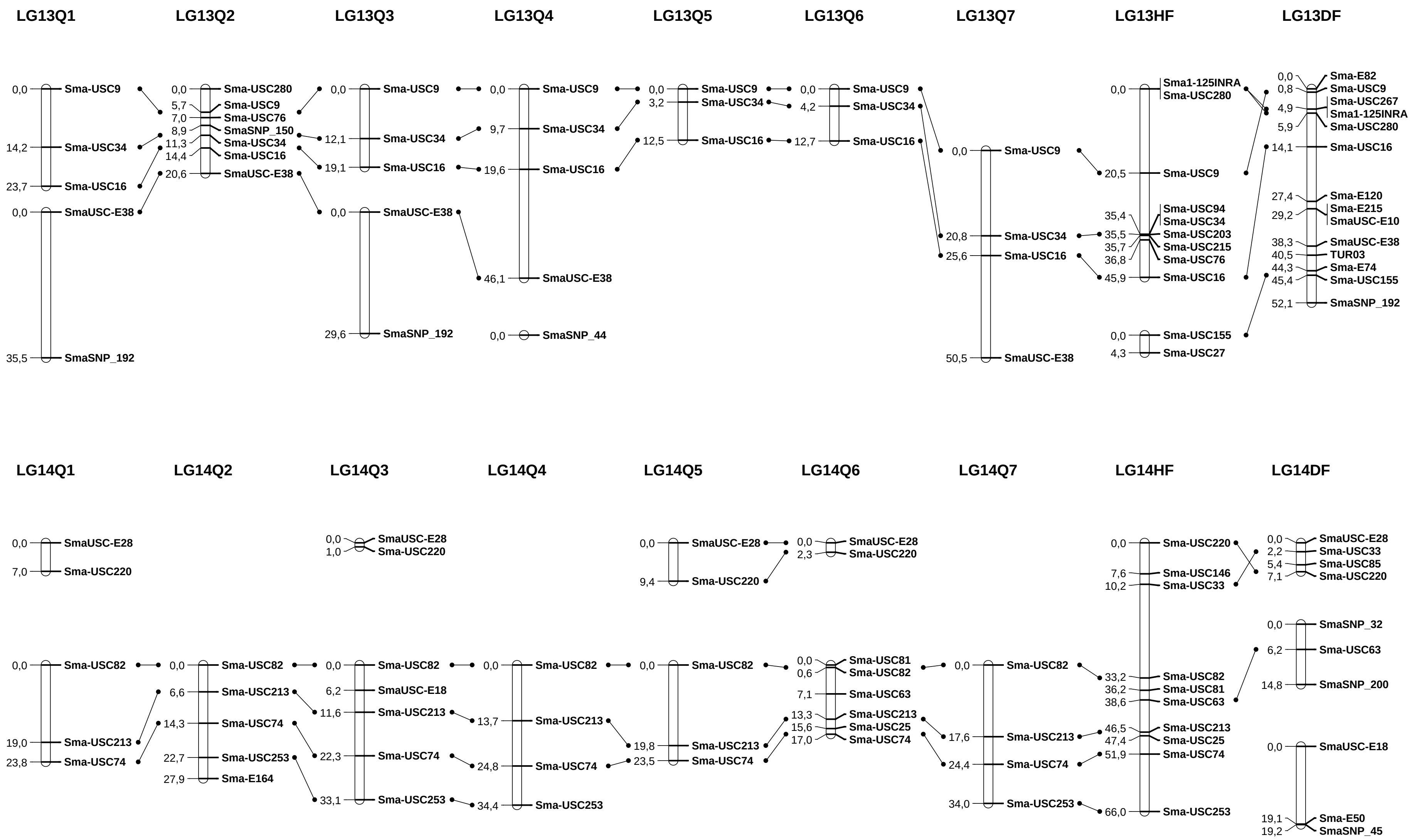
LG13Q1
0,0 Sma-USC9
14,2 Sma-USC34
23,7 Sma-USC16
0,0 SmaUSC-E38
35,5 SmaSNP_192
LG13Q2
0,0 Sma-USC280
5,7 Sma-USC9
7,0 Sma-USC76
8,9 SmaSNP_150
11,3 Sma-USC34
14,4 Sma-USC16
20,6 SmaUSC-E38
LG13Q3
0,0 Sma-USC9
12,1 Sma-USC34
19,1 Sma-USC16
0,0 SmaUSC-E38
29,6 SmaSNP_192
LG13Q4
0,0 Sma-USC9
9,7 Sma-USC34
19,6 Sma-USC16
46,1 SmaUSC-E38
0,0 SmaSNP_44
LG13Q5
0,0 Sma-USC9
3,2 Sma-USC34
12,5 Sma-USC16
LG13Q6
0,0 Sma-USC9
4,2 Sma-USC34
12,7 Sma-USC16
LG13Q7
0,0 Sma-USC9
20,8 Sma-USC34
25,6 Sma-USC16
50,5 SmaUSC-E38
LG13HF
0,0 Sma1-125INRA
Sma-USC280
20,5 Sma-USC9
35,4 Sma-USC94
35,5 Sma-USC34
35,7 Sma-USC203
36,8 Sma-USC215
Sma-USC76
45,9 Sma-USC16
0,0 Sma-USC155
4,3 Sma-USC27
LG13DF
0,0 Sma-E82
0,8 Sma-USC9
4,9 Sma-USC267
Sma1-125INRA
5,9 Sma-USC280
14,1 Sma-USC16
27,4 Sma-E120
29,2 Sma-E215
SmaUSC-E10
38,3 SmaUSC-E38
40,5 TUR03
44,3 Sma-E74
45,4 Sma-USC155
52,1 SmaSNP_192
LG14Q1
0,0 SmaUSC-E28
7,0 Sma-USC220
0,0 Sma-USC82
19,0 Sma-USC213
23,8 Sma-USC74
LG14Q2
0,0 Sma-USC82
6,6 Sma-USC213
14,3 Sma-USC74
22,7 Sma-USC253
27,9 Sma-E164
LG14Q3
0,0 SmaUSC-E28
1,0 Sma-USC220
0,0 Sma-USC82
6,2 SmaUSC-E18
11,6 Sma-USC213
22,3 Sma-USC74
33,1 Sma-USC253
LG14Q4
0,0 Sma-USC82
13,7 Sma-USC213
24,8 Sma-USC74
34,4 Sma-USC253
LG14Q5
0,0 SmaUSC-E28
9,4 Sma-USC220
0,0 Sma-USC82
19,8 Sma-USC213
23,5 Sma-USC74
LG14Q6
0,0 SmaUSC-E28
2,3 Sma-USC220
0,0 Sma-USC81
0,6 Sma-USC82
7,1 Sma-USC63
13,3 Sma-USC213
15,6 Sma-USC25
17,0 Sma-USC74
LG14Q7
0,0 Sma-USC82
17,6 Sma-USC213
24,4 Sma-USC74
34,0 Sma-USC253
LG14HF
0,0 Sma-USC220
7,6 Sma-USC146
10,2 Sma-USC33
33,2 Sma-USC82
36,2 Sma-USC81
38,6 Sma-USC63
46,5 Sma-USC213
47,4 Sma-USC25
51,9 Sma-USC74
66,0 Sma-USC253
LG14DF
0,0 SmaUSC-E28
2,2 Sma-USC33
5,4 Sma-USC85
7,1 Sma-USC220
0,0 SmaSNP_32
6,2 Sma-USC63
14,8 SmaSNP_200
0,0 SmaUSC-E18
19,1 Sma-E50
19,2 SmaSNP_45

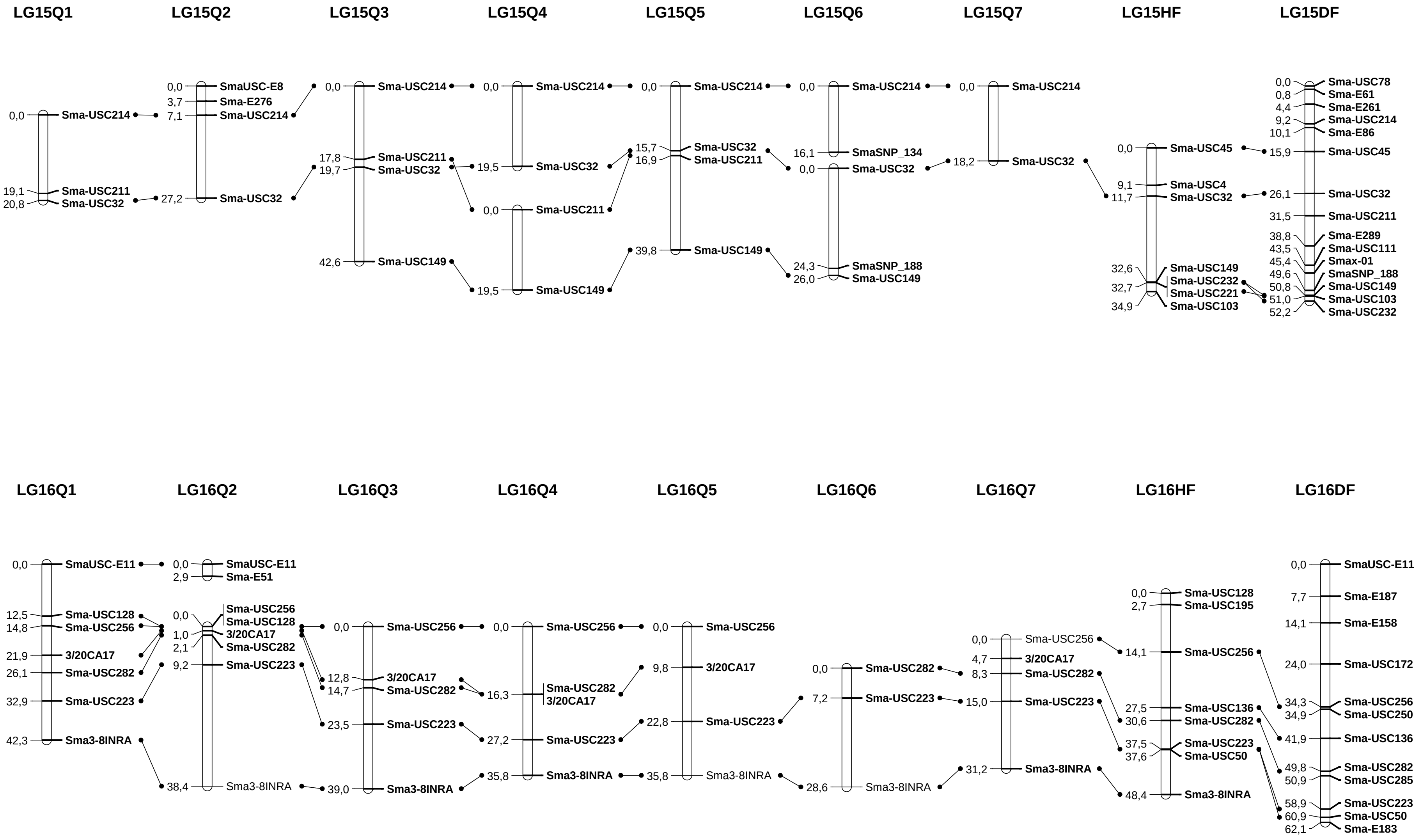

LG15Q1
0,0 Sma-USC214
19,1 Sma-USC211
20,8 Sma-USC32
LG15Q2
0,0 SmaUSC-E8
3,7 Sma-E276
7,1 Sma-USC214
27,2 Sma-USC32
LG15Q3
0,0 Sma-USC214
17,8 Sma-USC211
19,7 Sma-USC32
42,6 Sma-USC149
LG15Q4
0,0 Sma-USC214
19,5 Sma-USC32
0,0 Sma-USC211
19,5 Sma-USC149
LG15Q5
0,0 Sma-USC214
15,7 Sma-USC32
16,9 Sma-USC211
39,8 Sma-USC149
LG15Q6
0,0 Sma-USC214
16,1 SmaSNP_134
0,0 Sma-USC32
24,3 SmaSNP_188
26,0 Sma-USC149
LG15Q7
0,0 Sma-USC214
18,2 Sma-USC32
LG15HF
0,0 Sma-USC45
9,1 Sma-USC4
11,7 Sma-USC32
32,6 Sma-USC149
32,7 Sma-USC232
Sma-USC221
34,9 Sma-USC103
LG15DF
0,0 Sma-USC78
0,8 Sma-E61
4,4 Sma-E261
9,2 Sma-USC214
10,1 Sma-E86
15,9 Sma-USC45
26,1 Sma-USC32
31,5 Sma-USC211
38,8 Sma-E289
43,5 Sma-USC111
45,4 Smax-01
49,6 SmaSNP_188
50,8 Sma-USC149
51,0 Sma-USC103
52,2 Sma-USC232
LG16Q1
0,0 SmaUSC-E11
12,5 Sma-USC128
14,8 Sma-USC256
21,9 3/20CA17
26,1 Sma-USC282
32,9 Sma-USC223
42,3 Sma3-8INRA
LG16Q2
0,0 SmaUSC-E11
2,9 Sma-E51
0,0 Sma-USC256
Sma-USC128
1,0 3/20CA17
2,1 Sma-USC282
9,2 Sma-USC223
38,4 Sma3-8INRA
LG16Q3
0,0 Sma-USC256
12,8 3/20CA17
14,7 Sma-USC282
23,5 Sma-USC223
39,0 Sma3-8INRA
LG16Q4
0,0 Sma-USC256
16,3 Sma-USC282
3/20CA17
27,2 Sma-USC223
35,8 Sma3-8INRA
LG16Q5
0,0 Sma-USC256
9,8 3/20CA17
22,8 Sma-USC223
35,8 Sma3-8INRA
LG16Q6
0,0 Sma-USC282
7,2 Sma-USC223
28,6 Sma3-8INRA
LG16Q7
0,0 Sma-USC256
4,7 3/20CA17
8,3 Sma-USC282
15,0 Sma-USC223
31,2 Sma3-8INRA
LG16HF
0,0 Sma-USC128
2,7 Sma-USC195
14,1 Sma-USC256
27,5 Sma-USC136
30,6 Sma-USC282
37,5 Sma-USC223
37,6 Sma-USC50
48,4 Sma3-8INRA
LG16DF
0,0 SmaUSC-E11
7,7 Sma-E187
14,1 Sma-E158
24,0 Sma-USC172
34,3 Sma-USC256
34,9 Sma-USC250
41,9 Sma-USC136
49,8 Sma-USC282
50,9 Sma-USC285
58,9 Sma-USC223
60,9 Sma-USC50
62,1 Sma-E183

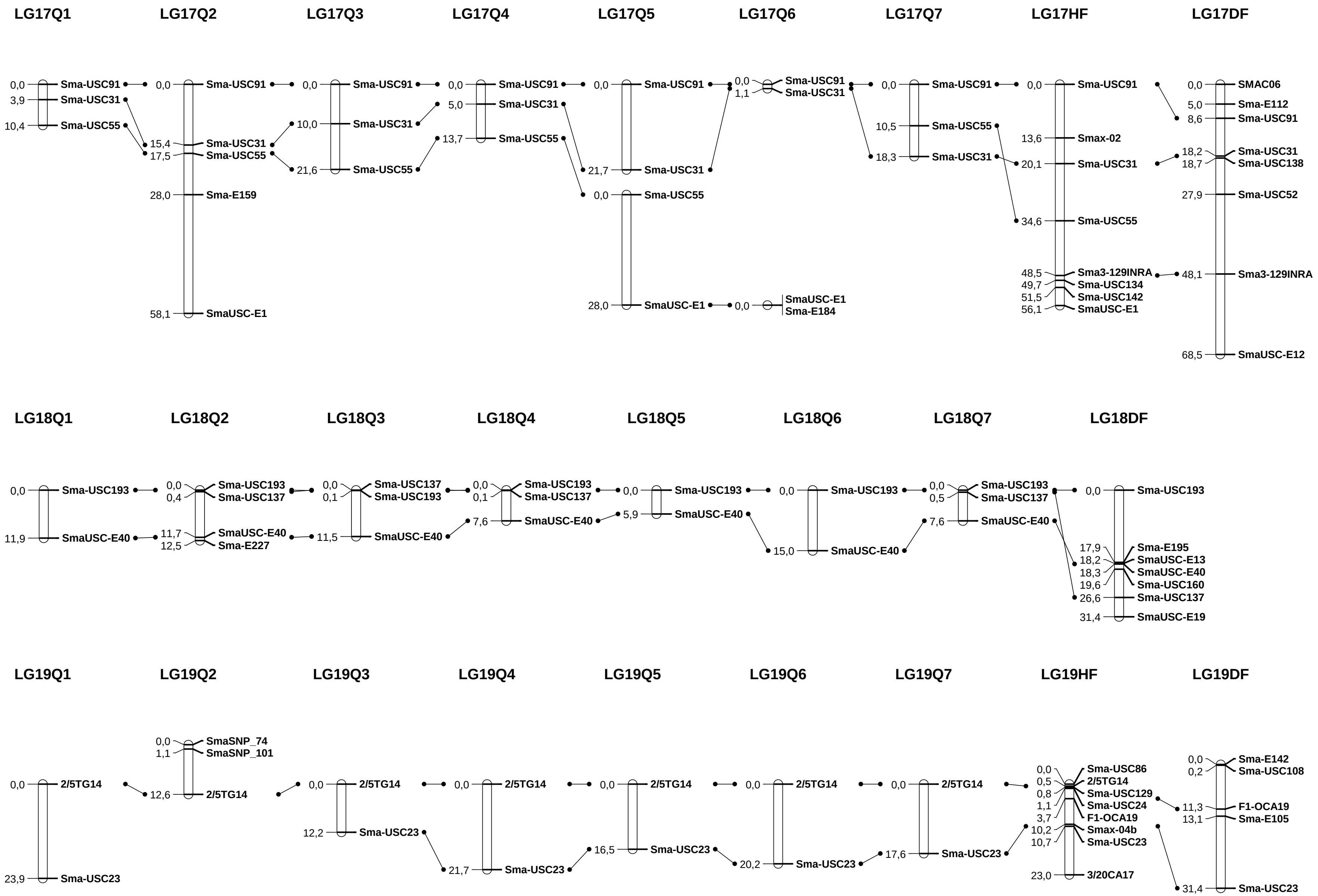
LG17Q1
0,0 Sma-USC91
3,9 Sma-USC31
10,4 Sma-USC55
LG17Q2
0,0 Sma-USC91
15,4 Sma-USC31
17,5 Sma-USC55
28,0 Sma-E159
58,1 SmaUSC-E1
LG17Q3
0,0 Sma-USC91
10,0 Sma-USC31
21,6 Sma-USC55
LG17Q4
0,0 Sma-USC91
5,0 Sma-USC31
13,7 Sma-USC55
LG17Q5
0,0 Sma-USC91
21,7 Sma-USC31
0,0 Sma-USC55
28,0 SmaUSC-E1
LG17Q6
0,0 Sma-USC91
1,1 Sma-USC31
0,0 SmaUSC-E1
Sma-E184
LG17Q7
0,0 Sma-USC91
10,5 Sma-USC55
18,3 Sma-USC31
LG17HF
0,0 Sma-USC91
13,6 Smax-02
20,1 Sma-USC31
34,6 Sma-USC55
48,5 Sma3-129INRA
49,7 Sma-USC134
51,5 Sma-USC142
56,1 SmaUSC-E1
LG17DF
0,0 SMAC06
5,0 Sma-E112
8,6 Sma-USC91
18,2 Sma-USC31
18,7 Sma-USC138
27,9 Sma-USC52
48,1 Sma3-129INRA
68,5 SmaUSC-E12
LG18Q1
0,0 Sma-USC193
11,9 SmaUSC-E40
LG18Q2
0,0 Sma-USC193
0,4 Sma-USC137
11,7 SmaUSC-E40
12,5 Sma-E227
LG18Q3
0,0 Sma-USC137
0,1 Sma-USC193
11,5 SmaUSC-E40
LG18Q4
0,0 Sma-USC193
0,1 Sma-USC137
7,6 SmaUSC-E40
LG18Q5
0,0 Sma-USC193
5,9 SmaUSC-E40
LG18Q6
0,0 Sma-USC193
15,0 SmaUSC-E40
LG18Q7
0,0 Sma-USC193
0,5 Sma-USC137
7,6 SmaUSC-E40
LG18DF
0,0 Sma-USC193
17,9 Sma-E195
18,2 SmaUSC-E13
18,3 SmaUSC-E40
19,6 Sma-USC160
26,6 Sma-USC137
31,4 SmaUSC-E19
LG19Q1
0,0 2/5TG14
23,9 Sma-USC23
LG19Q2
0,0 SmaSNP_74
1,1 SmaSNP_101
12,6 2/5TG14
LG19Q3
0,0 2/5TG14
12,2 Sma-USC23
LG19Q4
0,0 2/5TG14
21,7 Sma-USC23
LG19Q5
0,0 2/5TG14
16,5 Sma-USC23
LG19Q6
0,0 2/5TG14
20,2 Sma-USC23
LG19Q7
0,0 2/5TG14
17,6 Sma-USC23
LG19HF
0,0 Sma-USC86
0,5 2/5TG14
0,8 Sma-USC129
1,1 Sma-USC24
3,7 F1-OCA19
10,2 Smax-04b
10,7 Sma-USC23
23,0 3/20CA17
LG19DF
0,0 Sma-E142
0,2 Sma-USC108
11,3 F1-OCA19
13,1 Sma-E105
31,4 Sma-USC23

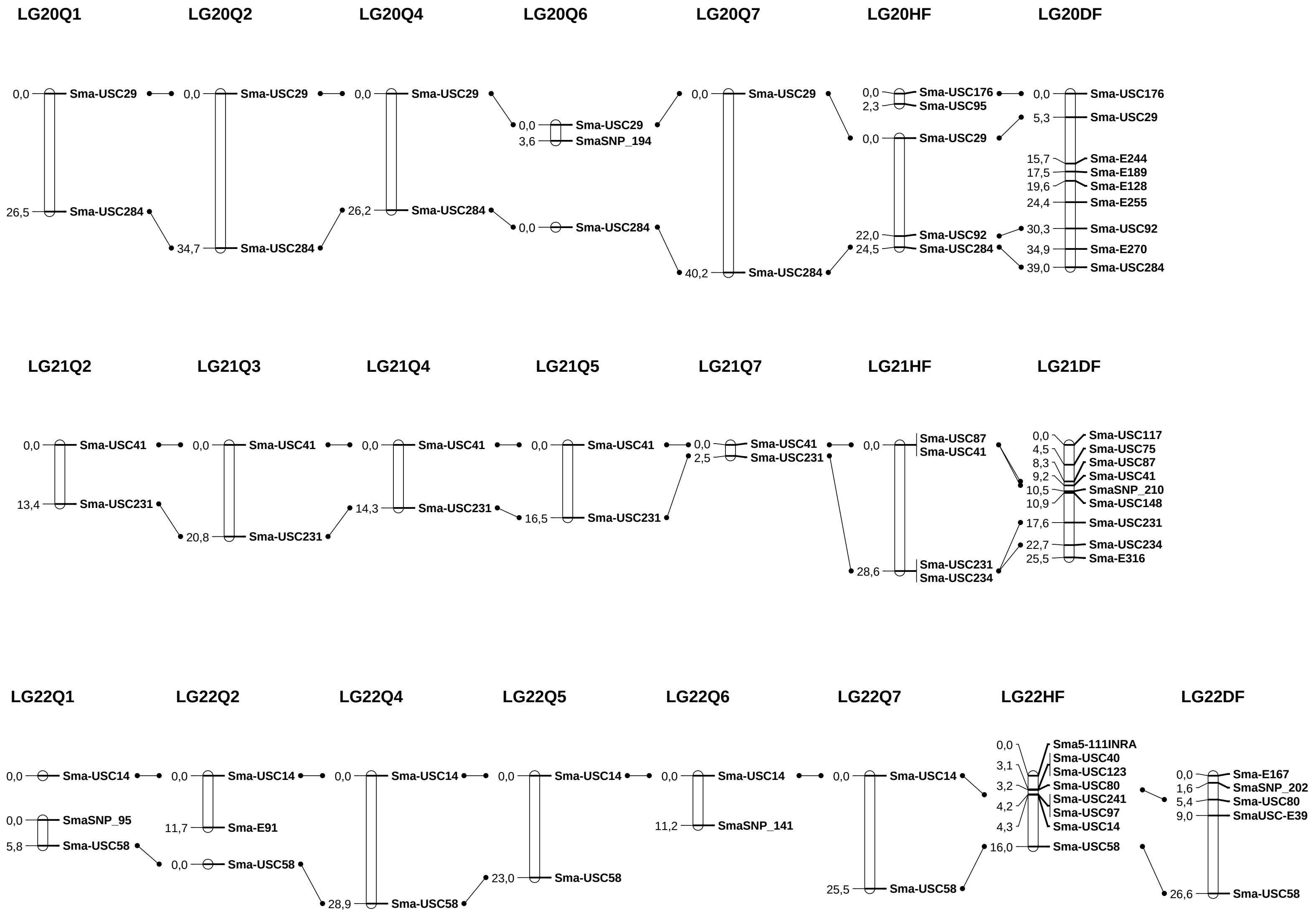

LG20Q1
0,0 Sma-USC29
26,5 Sma-USC284
LG20Q2
0,0 Sma-USC29
34,7 Sma-USC284
LG20Q4
0,0 Sma-USC29
26,2 Sma-USC284
LG20Q6
0,0 Sma-USC29
3,6 SmaSNP_194
0,0 Sma-USC284
LG20Q7
0,0 Sma-USC29
40,2 Sma-USC284
LG20HF
0,0 Sma-USC176
2,3 Sma-USC95
0,0 Sma-USC29
22,0 Sma-USC92
24,5 Sma-USC284
LG20DF
0,0 Sma-USC176
5,3 Sma-USC29
15,7 Sma-E244
17,5 Sma-E189
19,6 Sma-E128
24,4 Sma-E255
30,3 Sma-USC92
34,9 Sma-E270
39,0 Sma-USC284
LG21Q2
0,0 Sma-USC41
13,4 Sma-USC231
LG21Q3
0,0 Sma-USC41
20,8 Sma-USC231
LG21Q4
0,0 Sma-USC41
14,3 Sma-USC231
LG21Q5
0,0 Sma-USC41
16,5 Sma-USC231
LG21Q7
0,0 Sma-USC41
2,5 Sma-USC231
LG21HF
0,0 Sma-USC87
Sma-USC41
28,6 Sma-USC231
Sma-USC234
LG21DF
0,0 Sma-USC117
4,5 Sma-USC75
8,3 Sma-USC87
9,2 Sma-USC41
10,5 SmaSNP_210
10,9 Sma-USC148
17,6 Sma-USC231
22,7 Sma-USC234
25,5 Sma-E316
LG22Q1
0,0 Sma-USC14
0,0 SmaSNP_95
5,8 Sma-USC58
LG22Q2
0,0 Sma-USC14
11,7 Sma-E91
0,0 Sma-USC58
LG22Q4
0,0 Sma-USC14
28,9 Sma-USC58
LG22Q5
0,0 Sma-USC14
23,0 Sma-USC58
LG22Q6
0,0 Sma-USC14
11,2 SmaSNP_141
LG22Q7
0,0 Sma-USC14
25,5 Sma-USC58
LG22HF
0,0 Sma5-111INRA
3,1 Sma-USC40
3,2 Sma-USC123
4,2 Sma-USC80
4,3 Sma-USC241
Sma-USC97
Sma-USC14
16,0 Sma-USC58
LG22DF
0,0 Sma-E167
1,6 SmaSNP_202
5,4 Sma-USC80
9,0 SmaUSC-E39
26,6 Sma-USC58

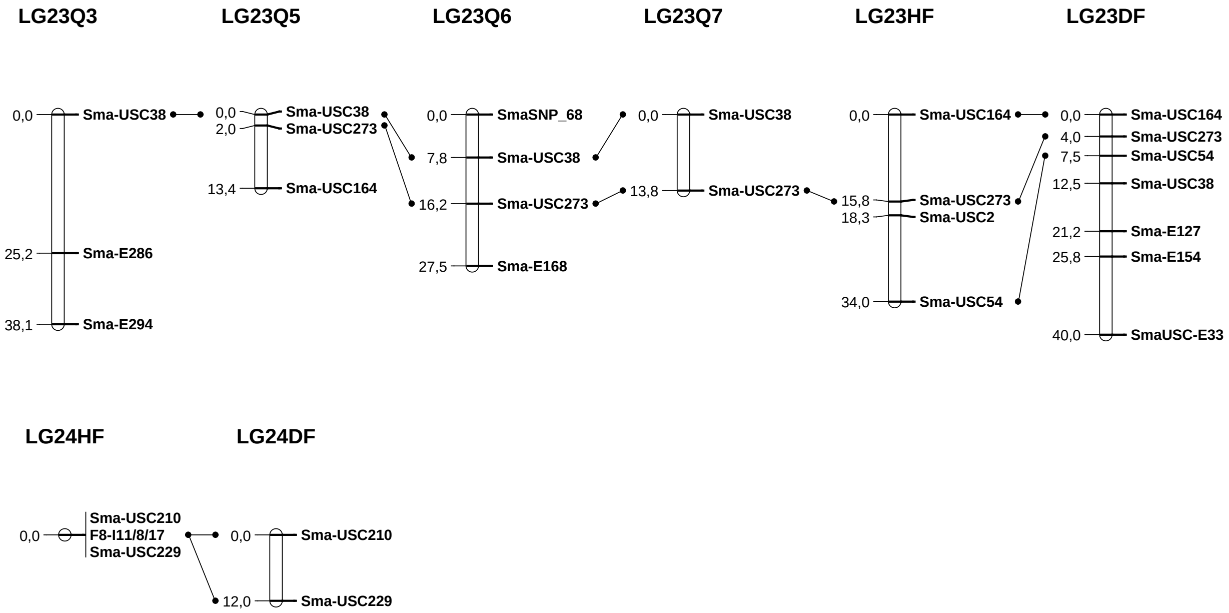

FIGURE S1.- Correspondence between the nine family maps used to construct the turbot consensus map.
