# Supplementary material for: An Expressed Sequence Tag (EST)-enriched genetic map of turbot (Scophthalmus maximus): a useful framework for comparative genomics across model and farmed teleosts
Source: BMC Genet. 2012 Jul 2;13:54. doi: 10.1186/1471-2156-13-54 (PMC3464660; doi:10.1186/1471-2156-13-54)
Supplement: Additional file 2 — Table S2. Informative markers used to construct the turbot consensus map. [file 1471-2156-13-54-S2.pdf]

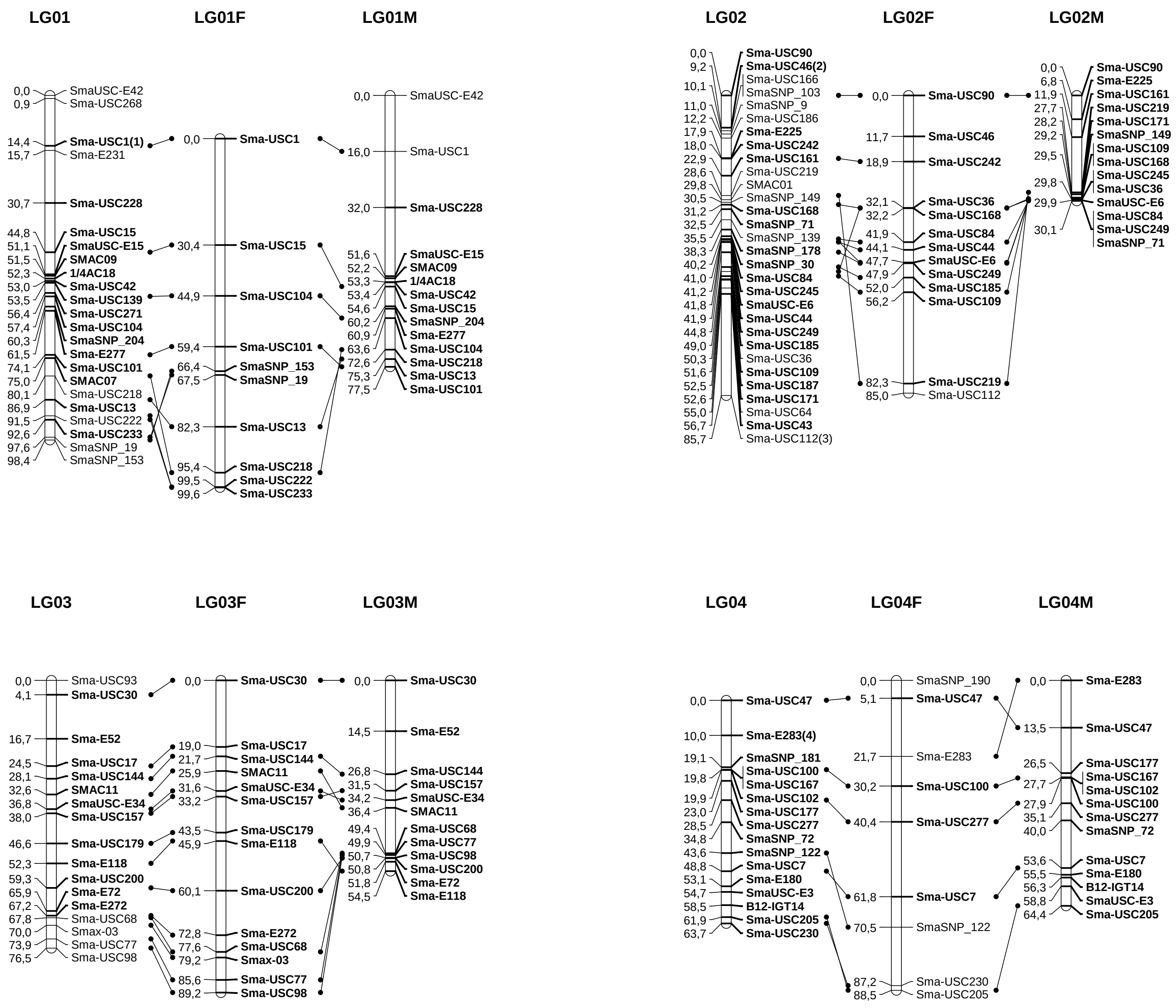
LG01
0,0 SmaUSC-E42
0,9 Sma-USC268
14,4 Sma-USC1(1)
15,7 Sma-E231
30,7 Sma-USC228
44,8 Sma-USC15
51,1 SmaUSC-E15
51,5 SMAC09
52,3 1/4AC18
53,0 Sma-USC42
53,5 Sma-USC139
56,4 Sma-USC271
57,4 Sma-USC104
60,3 SmaSNP_204
61,5 Sma-E277
74,1 Sma-USC101
75,0 SMAC07
80,1 Sma-USC218
86,9 Sma-USC13
91,5 Sma-USC222
92,6 Sma-USC233
97,6 SmaSNP_19
98,4 SmaSNP_153
LG01F
0,0 Sma-USC1
30,4 Sma-USC15
44,9 Sma-USC104
59,4 Sma-USC101
66,4 SmaSNP_153
67,5 SmaSNP_19
82,3 Sma-USC13
95,4 Sma-USC218
99,5 Sma-USC222
99,6 Sma-USC233
LG01M
0,0 SmaUSC-E42
16,0 Sma-USC1
32,0 Sma-USC228
51,6 SmaUSC-E15
52,2 SMAC09
53,3 1/4AC18
53,4 Sma-USC42
54,6 Sma-USC15
60,2 SmaSNP_204
60,9 Sma-E277
63,6 Sma-USC104
72,6 Sma-USC218
75,3 Sma-USC13
77,5 Sma-USC101
LG02
0,0
9,2
10,1
11,0
12,2
17,9
18,0
22,9
28,6
29,8
30,5
31,2
32,5
35,5
38,3
40,2
41,0
41,2
41,8
41,9
44,8
49,0
50,3
51,6
52,5
52,6
55,0
56,7
85,7
Sma-USC90
Sma-USC46(2)
Sma-USC166
SmaSNP_103
SmaSNP_9
Sma-USC186
Sma-E225
Sma-USC242
Sma-USC161
Sma-USC219
SMAC01
SmaSNP_149
Sma-USC168
SmaSNP_71
SmaSNP_139
SmaSNP_178
SmaSNP_30
Sma-USC84
Sma-USC245
SmaUSC-E6
Sma-USC44
Sma-USC249
Sma-USC185
Sma-USC36
Sma-USC109
Sma-USC187
Sma-USC171
Sma-USC64
Sma-USC43
Sma-USC112(3)
LG02F
0,0 Sma-USC90
11,7 Sma-USC46
18,9 Sma-USC242
32,1 Sma-USC36
32,2 Sma-USC168
41,9 Sma-USC84
44,1 Sma-USC44
47,7 SmaUSC-E6
47,9 Sma-USC249
52,0 Sma-USC185
56,2 Sma-USC109
82,3 Sma-USC219
85,0 Sma-USC112
LG02M
0,0
6,8
11,9
27,7
28,2
29,2
29,5
29,8
29,9
30,1
Sma-USC90
Sma-E225
Sma-USC161
Sma-USC219
Sma-USC171
SmaSNP_149
Sma-USC109
Sma-USC168
Sma-USC245
Sma-USC36
SmaUSC-E6
Sma-USC84
Sma-USC249
SmaSNP_71
LG03
0,0 Sma-USC93
4,1 Sma-USC30
16,7 Sma-E52
24,5 Sma-USC17
28,1 Sma-USC144
32,6 SMAC11
36,8 SmaUSC-E34
38,0 Sma-USC157
46,6 Sma-USC179
52,3 Sma-E118
59,3 Sma-USC200
65,9 Sma-E72
67,2 Sma-E272
67,8 Sma-USC68
70,0 Smax-03
73,9 Sma-USC77
76,5 Sma-USC98
LG03F
0,0 Sma-USC30
19,0 Sma-USC17
21,7 Sma-USC144
25,9 SMAC11
31,6 SmaUSC-E34
33,2 Sma-USC157
43,5 Sma-USC179
45,9 Sma-E118
60,1 Sma-USC200
72,8 Sma-E272
77,6 Sma-USC68
79,2 Smax-03
85,6 Sma-USC77
89,2 Sma-USC98
LG03M
0,0 Sma-USC30
14,5 Sma-E52
26,8 Sma-USC144
31,5 Sma-USC157
34,2 SmaUSC-E34
36,4 SMAC11
49,4 Sma-USC68
49,9 Sma-USC77
50,7 Sma-USC98
50,8 Sma-USC200
51,8 Sma-E72
54,5 Sma-E118
LG04
0,0
10,0
19,1
19,8
19,9
23,0
28,5
34,8
43,6
48,8
53,1
54,7
58,5
61,9
63,7
Sma-USC47
Sma-E283(4)
SmaSNP_181
Sma-USC100
Sma-USC167
Sma-USC102
Sma-USC177
Sma-USC277
SmaSNP_72
SmaSNP_122
Sma-USC7
Sma-E180
SmaUSC-E3
B12-IGT14
Sma-USC205
Sma-USC230
LG04F
0,0 SmaSNP_190
5,1 Sma-USC47
21,7 Sma-E283
30,2 Sma-USC100
40,4 Sma-USC277
61,8 Sma-USC7
70,5 SmaSNP_122
87,2 Sma-USC230
88,5 Sma-USC205
LG04M
0,0 Sma-E283
13,5 Sma-USC47
26,5
27,7
27,9
35,1
40,0
53,6
55,5
56,3
58,8
64,4
Sma-USC177
Sma-USC167
Sma-USC102
Sma-USC100
Sma-USC277
SmaSNP_72
Sma-USC7
Sma-E180
B12-IGT14
SmaUSC-E3
Sma-USC205

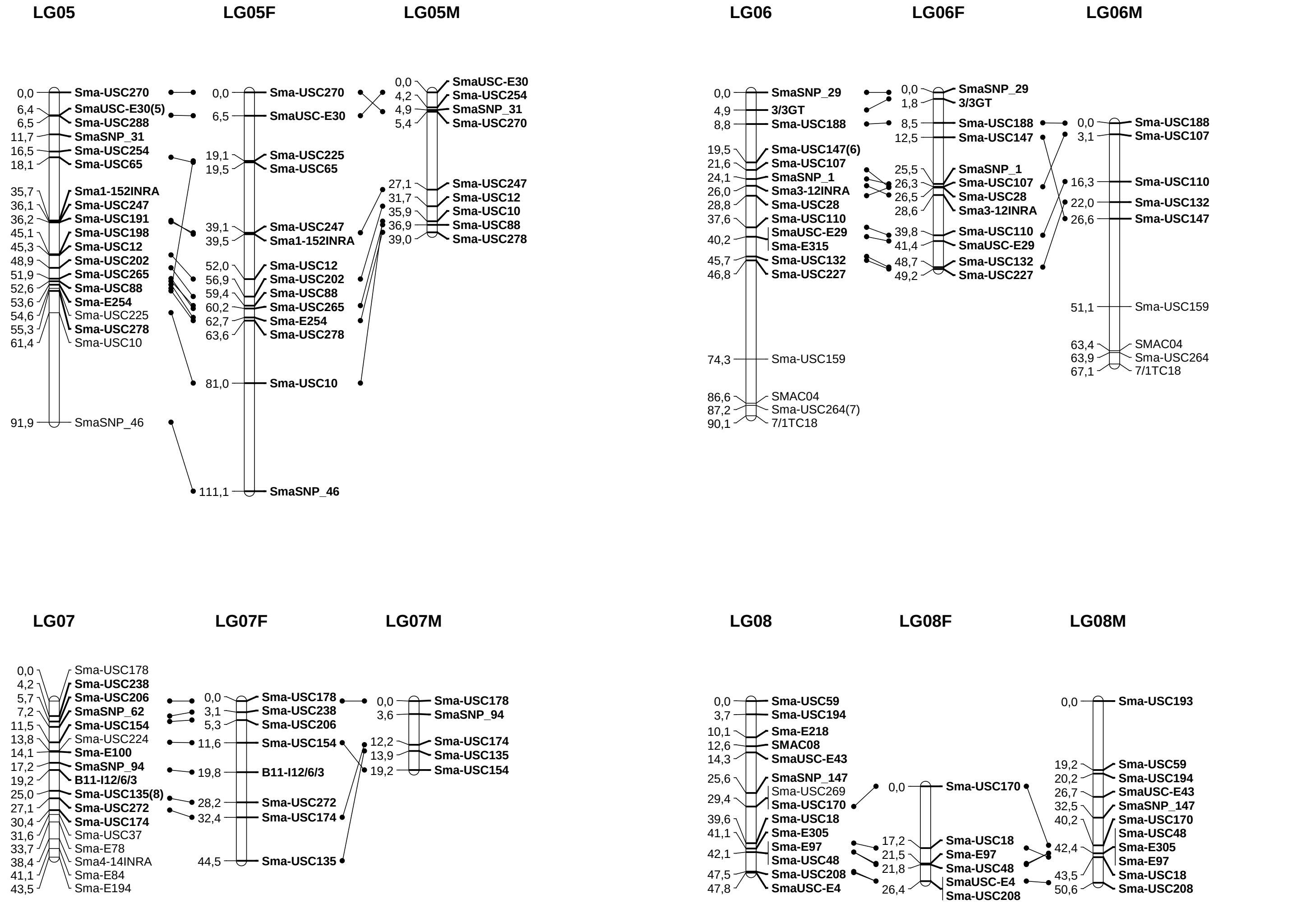
LG05
0,0 Sma-USC270
6,4 SmaUSC-E30(5)
6,5 Sma-USC288
11,7 SmaSNP_31
16,5 Sma-USC254
18,1 Sma-USC65
35,7 Sma1-152INRA
36,1 Sma-USC247
36,2 Sma-USC191
45,1 Sma-USC198
45,3 Sma-USC12
48,9 Sma-USC202
51,9 Sma-USC265
52,6 Sma-USC88
53,6 Sma-E254
54,6 Sma-USC225
55,3 Sma-USC278
61,4 Sma-USC10
91,9 SmaSNP_46
LG05F
0,0 Sma-USC270
6,5 SmaUSC-E30
19,1 Sma-USC225
19,5 Sma-USC65
39,1 Sma-USC247
39,5 Sma1-152INRA
52,0 Sma-USC12
56,9 Sma-USC202
59,4 Sma-USC88
60,2 Sma-USC265
62,7 Sma-E254
63,6 Sma-USC278
81,0 Sma-USC10
111,1 SmaSNP_46
LG05M
0,0 SmaUSC-E30
4,2 Sma-USC254
4,9 SmaSNP_31
5,4 Sma-USC270
27,1 Sma-USC247
31,7 Sma-USC12
35,9 Sma-USC10
36,9 Sma-USC88
39,0 Sma-USC278
LG06
0,0 SmaSNP_29
4,9 3/3GT
8,8 Sma-USC188
19,5 Sma-USC147(6)
21,6 Sma-USC107
24,1 SmaSNP_1
26,0 Sma3-12INRA
28,8 Sma-USC28
37,6 Sma-USC110
40,2 SmaUSC-E29
Sma-E315
45,7 Sma-USC132
46,8 Sma-USC227
74,3 Sma-USC159
86,6 SMAC04
87,2 Sma-USC264(7)
90,1 7/1TC18
LG06F
0,0 SmaSNP_29
1,8 3/3GT
8,5 Sma-USC188
12,5 Sma-USC147
25,5 SmaSNP_1
26,3 Sma-USC107
26,5 Sma-USC28
28,6 Sma3-12INRA
39,8 Sma-USC110
41,4 SmaUSC-E29
48,7 Sma-USC132
49,2 Sma-USC227
LG06M
0,0 Sma-USC188
3,1 Sma-USC107
16,3 Sma-USC110
22,0 Sma-USC132
26,6 Sma-USC147
51,1 Sma-USC159
63,4 SMAC04
63,9 Sma-USC264
67,1 7/1TC18
LG07
0,0 Sma-USC178
4,2 Sma-USC238
5,7 Sma-USC206
7,2 SmaSNP_62
11,5 Sma-USC154
13,8 Sma-USC224
14,1 Sma-E100
17,2 SmaSNP_94
19,2 B11-I12/6/3
25,0 Sma-USC135(8)
27,1 Sma-USC272
30,4 Sma-USC174
31,6 Sma-USC37
33,7 Sma-E78
38,4 Sma4-14INRA
41,1 Sma-E84
43,5 Sma-E194
LG07F
0,0 Sma-USC178
3,1 Sma-USC238
5,3 Sma-USC206
11,6 Sma-USC154
19,8 B11-I12/6/3
28,2 Sma-USC272
32,4 Sma-USC174
44,5 Sma-USC135
LG07M
0,0 Sma-USC178
3,6 SmaSNP_94
12,2 Sma-USC174
13,9 Sma-USC135
19,2 Sma-USC154
LG08
0,0 Sma-USC59
3,7 Sma-USC194
10,1 Sma-E218
12,6 SMAC08
14,3 SmaUSC-E43
25,6 SmaSNP_147
Sma-USC269
29,4 Sma-USC170
39,6 Sma-USC18
41,1 Sma-E305
42,1 Sma-E97
Sma-USC48
47,5 Sma-USC208
47,8 SmaUSC-E4
LG08F
0,0 Sma-USC170
17,2 Sma-USC18
21,5 Sma-E97
21,8 Sma-USC48
26,4 SmaUSC-E4
Sma-USC208
LG08M
0,0 Sma-USC193
19,2 Sma-USC59
20,2 Sma-USC194
26,7 SmaUSC-E43
32,5 SmaSNP_147
40,2 Sma-USC170
Sma-USC48
42,4 Sma-E305
Sma-E97
43,5 Sma-USC18
50,6 Sma-USC208

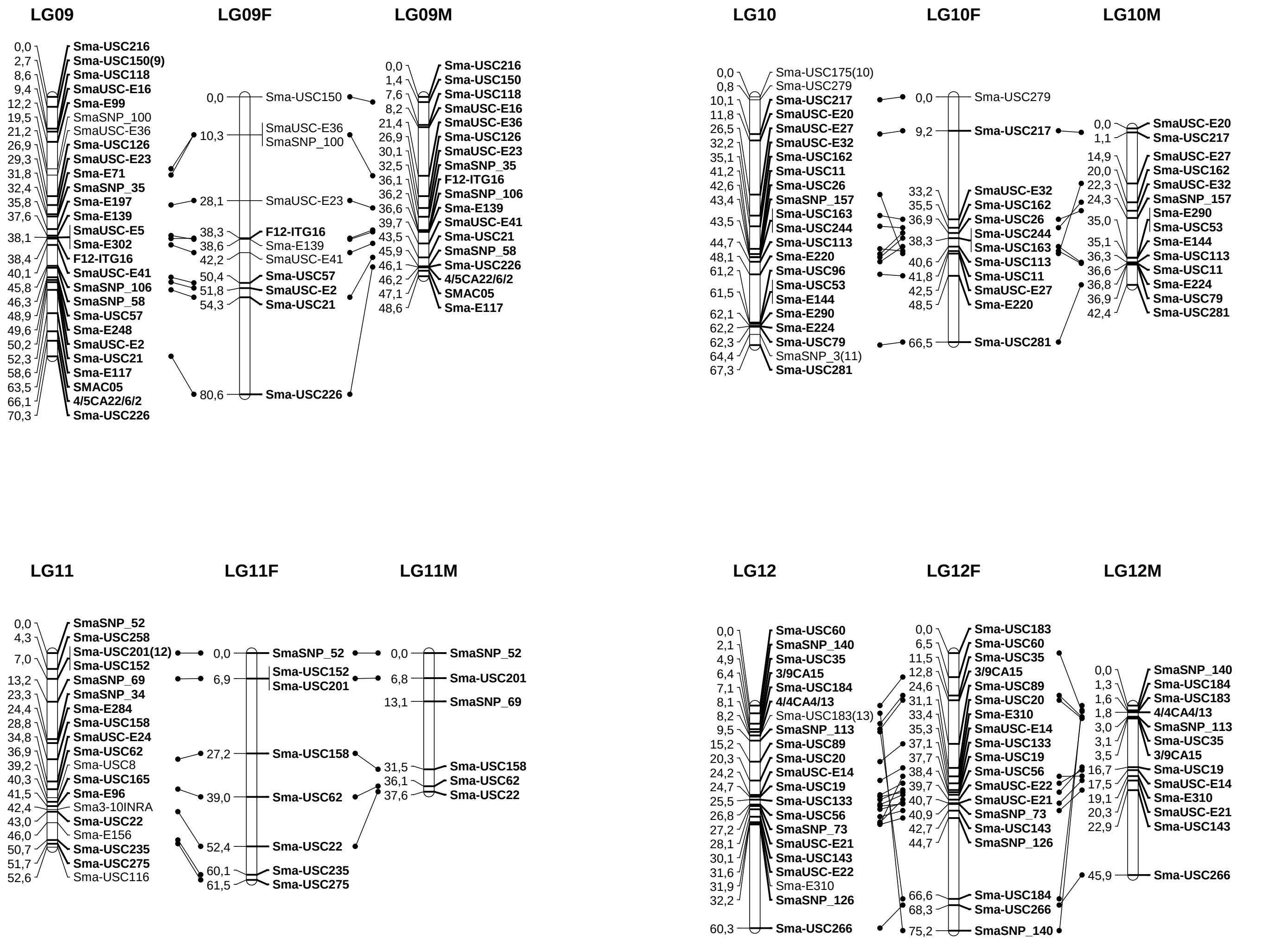

LG09
0,0 Sma-USC216
2,7 Sma-USC150(9)
8,6 Sma-USC118
9,4 SmaUSC-E16
12,2 Sma-E99
19,5 SmaSNP_100
21,2 SmaUSC-E36
26,9 Sma-USC126
29,3 SmaUSC-E23
31,8 Sma-E71
32,4 SmaSNP_35
35,8 Sma-E197
37,6 Sma-E139
38,1 SmaUSC-E5
Sma-E302
38,4 F12-ITG16
40,1 SmaUSC-E41
45,8 SmaSNP_106
46,3 SmaSNP_58
48,9 Sma-USC57
49,6 Sma-E248
50,2 SmaUSC-E2
52,3 Sma-USC21
58,6 Sma-E117
63,5 SMAC05
66,1 4/5CA22/6/2
70,3 Sma-USC226
LG09F
0,0 Sma-USC150
10,3 SmaUSC-E36
SmaSNP_100
28,1 SmaUSC-E23
38,3 F12-ITG16
38,6 Sma-E139
42,2 SmaUSC-E41
50,4 Sma-USC57
51,8 SmaUSC-E2
54,3 Sma-USC21
80,6 Sma-USC226
LG09M
0,0 Sma-USC216
1,4 Sma-USC150
7,6 Sma-USC118
8,2 SmaUSC-E16
21,4 SmaUSC-E36
26,9 Sma-USC126
30,1 SmaUSC-E23
32,5 SmaSNP_35
36,1 F12-ITG16
36,2 SmaSNP_106
36,6 Sma-E139
39,7 SmaUSC-E41
43,5 Sma-USC21
45,9 SmaSNP_58
46,1 Sma-USC226
46,2 4/5CA22/6/2
47,1 SMAC05
48,6 Sma-E117
LG10
0,0 Sma-USC175(10)
0,8 Sma-USC279
10,1 Sma-USC217
11,8 SmaUSC-E20
26,5 SmaUSC-E27
32,2 SmaUSC-E32
35,1 Sma-USC162
41,2 Sma-USC11
42,6 Sma-USC26
43,4 SmaSNP_157
43,5 Sma-USC163
44,7 Sma-USC244
48,1 Sma-USC113
61,2 Sma-E220
61,5 Sma-USC96
62,1 Sma-USC53
62,2 Sma-E144
62,3 Sma-E290
64,4 Sma-E224
67,3 Sma-USC79
SmaSNP_3(11)
Sma-USC281
LG10F
0,0 Sma-USC279
9,2 Sma-USC217
33,2 SmaUSC-E32
35,5 Sma-USC162
36,9 Sma-USC26
38,3 Sma-USC244
Sma-USC163
40,6 Sma-USC113
41,8 Sma-USC11
42,5 SmaUSC-E27
48,5 Sma-E220
66,5 Sma-USC281
LG10M
0,0 SmaUSC-E20
1,1 Sma-USC217
14,9 SmaUSC-E27
20,0 Sma-USC162
22,3 SmaUSC-E32
24,3 SmaSNP_157
35,0 Sma-E290
Sma-USC53
35,1 Sma-E144
36,3 Sma-USC113
36,6 Sma-USC11
36,8 Sma-E224
36,9 Sma-USC79
42,4 Sma-USC281
LG11
0,0 SmaSNP_52
4,3 Sma-USC258
7,0 Sma-USC201(12)
Sma-USC152
13,2 SmaSNP_69
23,3 SmaSNP_34
24,4 Sma-E284
28,8 Sma-USC158
34,8 SmaUSC-E24
36,9 Sma-USC62
39,2 Sma-USC8
40,3 Sma-USC165
41,5 Sma-E96
42,4 Sma3-10INRA
43,0 Sma-USC22
46,0 Sma-E156
50,7 Sma-USC235
51,7 Sma-USC275
52,6 Sma-USC116
LG11F
0,0 SmaSNP_52
6,9 Sma-USC152
Sma-USC201
27,2 Sma-USC158
39,0 Sma-USC62
52,4 Sma-USC22
60,1 Sma-USC235
61,5 Sma-USC275
LG11M
0,0 SmaSNP_52
6,8 Sma-USC201
13,1 SmaSNP_69
31,5 Sma-USC158
36,1 Sma-USC62
37,6 Sma-USC22
LG12
0,0 Sma-USC60
2,1 SmaSNP_140
4,9 Sma-USC35
6,4 3/9CA15
7,1 Sma-USC184
8,1 4/4CA4/13
8,2 Sma-USC183(13)
9,5 SmaSNP_113
15,2 Sma-USC89
20,3 Sma-USC20
24,2 SmaUSC-E14
24,7 Sma-USC19
25,5 Sma-USC133
26,8 Sma-USC56
27,2 SmaSNP_73
28,1 SmaUSC-E21
30,1 Sma-USC143
31,6 SmaUSC-E22
31,9 Sma-E310
32,2 SmaSNP_126
60,3 Sma-USC266
LG12F
0,0 Sma-USC183
6,5 Sma-USC60
11,5 Sma-USC35
12,8 3/9CA15
24,6 Sma-USC89
31,1 Sma-USC20
33,4 Sma-E310
35,3 SmaUSC-E14
37,1 Sma-USC133
37,7 Sma-USC19
38,4 Sma-USC56
39,7 SmaUSC-E22
40,7 SmaUSC-E21
40,9 SmaSNP_73
42,7 Sma-USC143
44,7 SmaSNP_126
66,6 Sma-USC184
68,3 Sma-USC266
75,2 SmaSNP_140
LG12M
0,0 SmaSNP_140
1,3 Sma-USC184
1,6 Sma-USC183
1,8 4/4CA4/13
3,0 SmaSNP_113
3,1 Sma-USC35
3,5 3/9CA15
16,7 Sma-USC19
17,5 SmaUSC-E14
19,1 Sma-E310
20,3 SmaUSC-E21
22,9 Sma-USC143
45,9 Sma-USC266

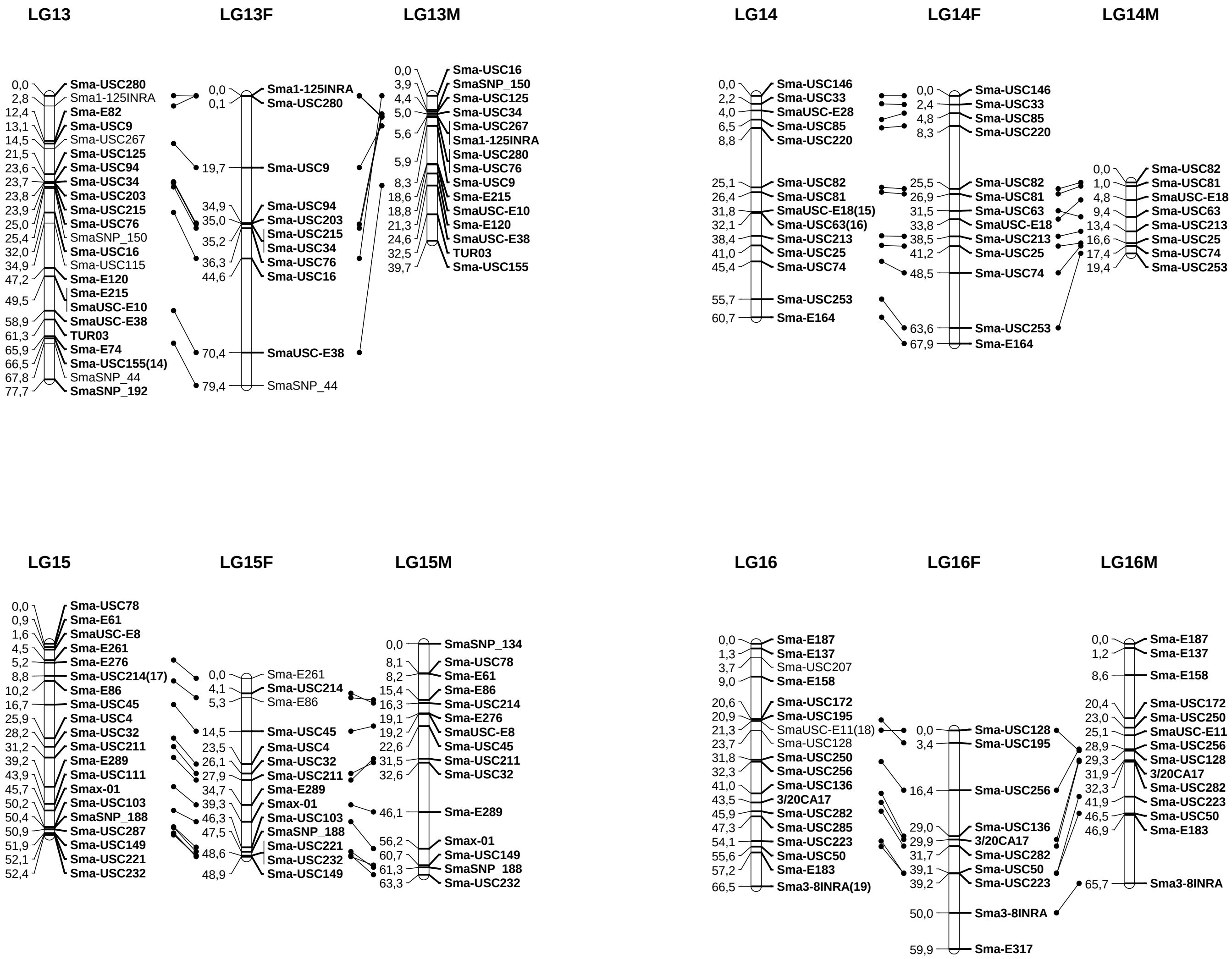
LG13
LG13F
LG13M
LG14
LG14F
LG14M
LG15
LG15F
LG15M
LG16
LG16F
LG16M
0,0 Sma-USC280
2,8 Sma1-125INRA
12,4 Sma-E82
13,1 Sma-USC9
14,5 Sma-USC267
21,5 Sma-USC125
23,6 Sma-USC94
23,7 Sma-USC34
23,8 Sma-USC203
23,9 Sma-USC215
25,0 Sma-USC76
25,4 SmaSNP_150
32,0 Sma-USC16
34,9 Sma-USC115
47,2 Sma-E120
49,5 Sma-E215
SmaUSC-E10
58,9 SmaUSC-E38
61,3 TUR03
65,9 Sma-E74
66,5 Sma-USC155(14)
67,8 SmaSNP_44
77,7 SmaSNP_192
0,0 Sma1-125INRA
0,1 Sma-USC280
19,7 Sma-USC9
34,9 Sma-USC94
35,0 Sma-USC203
35,2 Sma-USC215
36,3 Sma-USC34
44,6 Sma-USC76
Sma-USC16
70,4 SmaUSC-E38
79,4 SmaSNP_44
0,0 Sma-USC16
3,9 SmaSNP_150
4,4 Sma-USC125
5,0 Sma-USC34
5,6 Sma-USC267
Sma1-125INRA
5,9 Sma-USC280
Sma-USC76
8,3 Sma-USC9
18,6 Sma-E215
18,8 SmaUSC-E10
21,3 Sma-E120
24,6 SmaUSC-E38
32,5 TUR03
39,7 Sma-USC155
0,0 Sma-USC146
2,2 Sma-USC33
4,0 SmaUSC-E28
6,5 Sma-USC85
8,8 Sma-USC220
25,1 Sma-USC82
26,4 Sma-USC81
31,8 SmaUSC-E18(15)
32,1 Sma-USC63(16)
38,4 Sma-USC213
41,0 Sma-USC25
45,4 Sma-USC74
55,7 Sma-USC253
60,7 Sma-E164
0,0 Sma-USC146
2,4 Sma-USC33
4,8 Sma-USC85
8,3 Sma-USC220
25,5 Sma-USC82
26,9 Sma-USC81
31,5 Sma-USC63
33,8 SmaUSC-E18
38,5 Sma-USC213
41,2 Sma-USC25
48,5 Sma-USC74
63,6 Sma-USC253
67,9 Sma-E164
0,0 Sma-USC82
1,0 Sma-USC81
4,8 SmaUSC-E18
9,4 Sma-USC63
13,4 Sma-USC213
16,6 Sma-USC25
17,4 Sma-USC74
19,4 Sma-USC253
0,0 Sma-USC78
0,9 Sma-E61
1,6 SmaUSC-E8
4,5 Sma-E261
5,2 Sma-E276
8,8 Sma-USC214(17)
10,2 Sma-E86
16,7 Sma-USC45
25,9 Sma-USC4
28,2 Sma-USC32
31,2 Sma-USC211
39,2 Sma-E289
43,9 Sma-USC111
45,7 Smax-01
50,2 Sma-USC103
50,4 SmaSNP_188
50,9 Sma-USC287
51,9 Sma-USC149
52,1 Sma-USC221
52,4 Sma-USC232
0,0 Sma-E261
4,1 Sma-USC214
5,3 Sma-E86
14,5 Sma-USC45
23,5 Sma-USC4
26,1 Sma-USC32
27,9 Sma-USC211
34,7 Sma-E289
39,3 Smax-01
46,3 Sma-USC103
47,5 SmaSNP_188
48,6 Sma-USC221
Sma-USC232
48,9 Sma-USC149
0,0 SmaSNP_134
8,1 Sma-USC78
8,2 Sma-E61
15,4 Sma-E86
16,3 Sma-USC214
19,1 Sma-E276
19,2 SmaUSC-E8
22,6 Sma-USC45
31,5 Sma-USC211
32,6 Sma-USC32
46,1 Sma-E289
56,2 Smax-01
60,7 Sma-USC149
61,3 SmaSNP_188
63,3 Sma-USC232
0,0 Sma-E187
1,3 Sma-E137
3,7 Sma-USC207
9,0 Sma-E158
20,6 Sma-USC172
20,9 Sma-USC195
21,3 SmaUSC-E11(18)
23,7 Sma-USC128
31,8 Sma-USC250
32,3 Sma-USC256
41,0 Sma-USC136
43,5 3/20CA17
45,9 Sma-USC282
47,3 Sma-USC285
54,1 Sma-USC223
55,6 Sma-USC50
57,2 Sma-E183
66,5 Sma3-8INRA(19)
0,0 Sma-USC128
3,4 Sma-USC195
16,4 Sma-USC256
29,0 Sma-USC136
29,9 3/20CA17
31,7 Sma-USC282
39,1 Sma-USC50
39,2 Sma-USC223
50,0 Sma3-8INRA
59,9 Sma-E317
0,0 Sma-E187
1,2 Sma-E137
8,6 Sma-E158
20,4 Sma-USC172
23,0 Sma-USC250
25,1 SmaUSC-E11
28,9 Sma-USC256
29,3 Sma-USC128
31,9 3/20CA17
32,3 Sma-USC282
41,9 Sma-USC223
46,5 Sma-USC50
46,9 Sma-E183
65,7 Sma3-8INRA

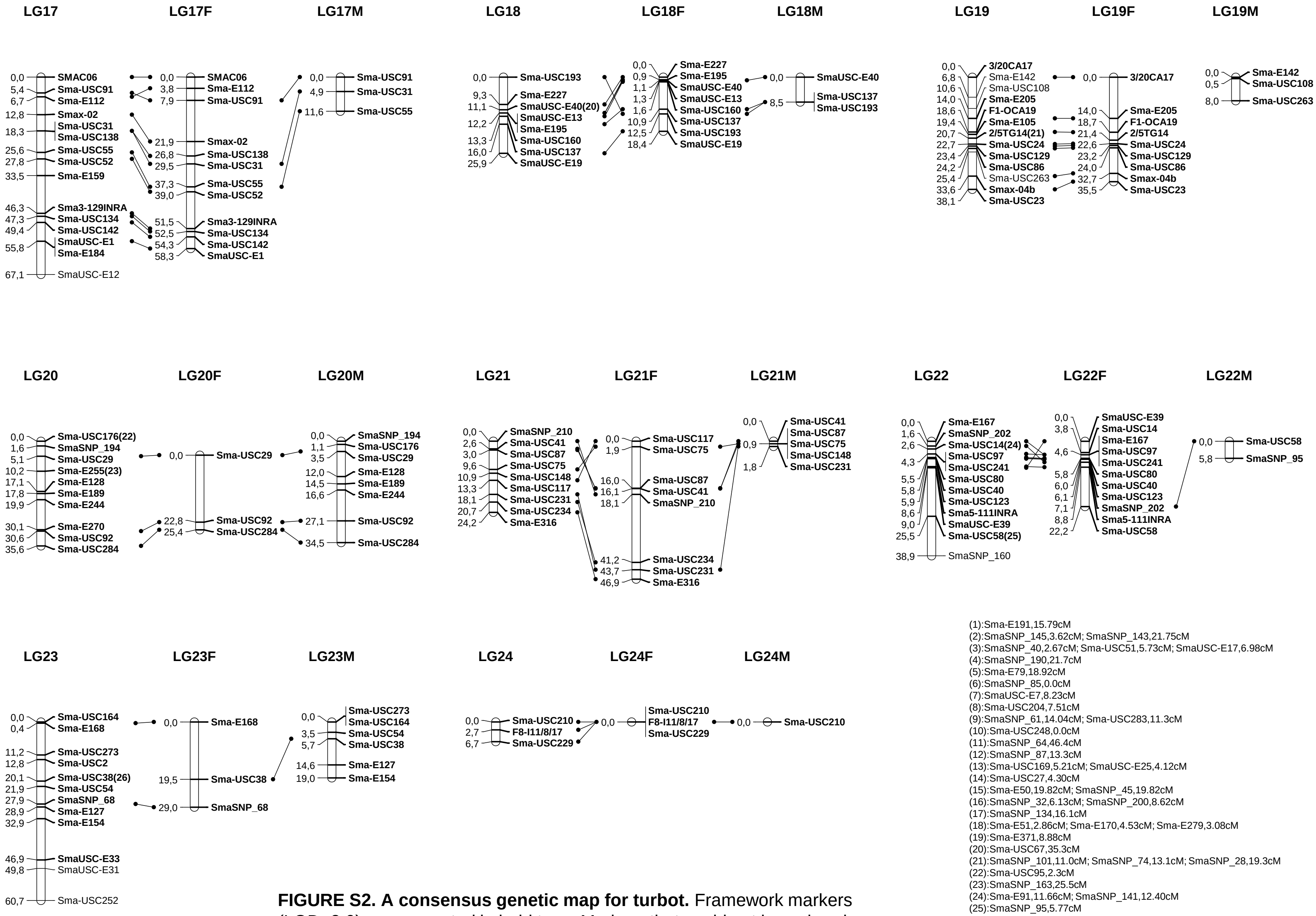


(1):Sma-E191,15.79cM
(2):SmaSNP_145,3.62cM; SmaSNP_143,21.75cM
(3):SmaSNP_40,2.67cM; Sma-USC51,5.73cM; SmaUSC-E17,6.98cM
(4):SmaSNP_190,21.7cM
(5):Sma-E79,18.92cM
(6):SmaSNP_85,0.0cM
(7):SmaUSC-E7,8.23cM
(8):Sma-USC204,7.51cM
(9):SmaSNP_61,14.04cM; Sma-USC283,11.3cM
(10):Sma-USC248,0.0cM
(11):SmaSNP_64,46.4cM
(12):SmaSNP_87,13.3cM
(13):Sma-USC169,5.21cM; SmaUSC-E25,4.12cM
(14):Sma-USC27,4.30cM
(15):Sma-E50,19.82cM; SmaSNP_45,19.82cM
(16):SmaSNP_32,6.13cM; SmaSNP_200,8.62cM
(17):SmaSNP_134,16.1cM
(18):Sma-E51,2.86cM; Sma-E170,4.53cM; Sma-E279,3.08cM
(19):Sma-E371,8.88cM
(20):Sma-USC67,35.3cM
(21):SmaSNP_101,11.0cM; SmaSNP_74,13.1cM; SmaSNP_28,19.3cM
(22):Sma-USC95,2.3cM
(23):SmaSNP_163,25.5cM
(24):Sma-E91,11.66cM; SmaSNP_141,12.40cM
(25):SmaSNP_95,5.77cM
(26):Sma-E286,25.21cM; Sma-E294,38.15cM

**FIGURE S2. A consensus genetic map for turbot.** Framework markers (LOD>3.0) are presented in bold type. Markers that could not be ordered with a log-likelihood support are represented as accessory markers at the right of the nearest linked marker
