# Supplementary material for: An Expressed Sequence Tag (EST)-enriched genetic map of turbot (Scophthalmus maximus): a useful framework for comparative genomics across model and farmed teleosts
Source: BMC Genet. 2012 Jul 2;13:54. doi: 10.1186/1471-2156-13-54 (PMC3464660; doi:10.1186/1471-2156-13-54)
Supplement: Additional file 3 — Figure S1. Correspondence between the nine family maps used to construct the turbot consensus map. [file 1471-2156-13-54-S3.pdf]

LG01	Gac	Tetr	Ola	Tru	Dre
Sma-E191	-	6	-	10	2
SmaUSC-E42	sc120	15-un	17	22un	-
Sma-USC268	2	5	-	-	25
Sma-USC1	18	14	24	16	-
Sma-E231	3	un	17	-	-
Sma-USC15	3	15	17	22un	-
SmaUSC-E15	3	-	17	-	-
SMAC09	3	un	17	22	-
1/4AC18	3	15	17	22	22
Sma-USC271	3	-	-	-	-
SmaSNP204	3	15	17	22un	2
Sma-E277	3	-	-	22	-
SMAC07	3	un	17	22	-
Sma-USC218	sc111	un	-	12un	-
Sma-USC222	10	21	11	12	-
SmaSNP19	10	un	11	12	19
SmaSNP153	10	un	11	12	19

LG02	Gac	Tetr	Ola	Tru	Dre
SmaSNP143	20	8	16	7	-
SmaSNP145	20	8	16	7	16
SmaSNP40	6	17		4	-
Sma-USC51	-	2	19	1	-
SmaUSC-E17	6	-	-	-	-
Sma-USC46	20	8	16	7	-
SmaSNP103	1	3	-	8	7
Sma-USC242	20	-	-	-	-
SMAC01	sc47	-	-	-	7
SmaSNP149	1	3	-	-	-
Sma-USC168	20	8	-	-	-
SmaSNP71	20	8	-	-	-
SmaSNP30	20	8	16	-	16
Sma-USC84	20	-	16	-	-
Sma-USC249	-	-	16	-	-
Sma-USC185	-	-	16	-	-
Sma-USC36	20	-	-	-	-
Sma-USC187	1	-	2	8	-
Sma-USC64	20	8	16	-	-
Sma-USC43	sc74	-	2	-	-

LG03	Gac	Tetr	Ola	Tru	Dre
Sma-USC93	15	un	sc604	2	-
Sma-USC17	-	10	-	2	-
SmaUSC-E34	15	10	22	2	-
Sma-USC157	15	10	22	2	-
Sma-USC179	15	10	-	-	-
Sma-E118	15	10	22	2	-
Sma-E72	15	10	22	2	-
Sma-E272	-	10	-	-	-

LG04	Gac	Tetr	Ola	Tru	Dre
SmaSNP190	2	5	3	13	18
SmaSNP181	2	5	3	13	7
Sma-USC100	2	5	3	13	-
Sma-USC167	-	-	-	17	-
Sma-USC102	2	5	3	13	sc_3485

LG05	Gac	Tetr	Ola	Tru	Dre
SmaUSC-E30	8	-	-	-	-
SmaSNP31	8	1	-	20	-
Sma-USC12	8	1	4	20	8
Sma-USC202	8	-	-	-	-
Sma-USC265	8	1	4	20	-
Sma-USC88	8	1	-	20	-
Sma-E254	-	-	4	-	-
Sma-USC225	8	un	-	20un	-

LG06	Gac	Tetr	Ola	Tru	Dre
SmaUSC-E7	19	13	uc72	9	-
SmaSNP29	19	13	-	-	25
Sma-USC188	19	-	-	-	-
Sma-USC107	19	13	-	9	-
Sma-USC110	19	13	6	9	18
SmaUSC-E29	19	13	6	9	25
Sma-E315	19	13	6	9	25
Sma-USC132	-	-	-	-	6
Sma-USC264	19	-	-	-	-

LG07	Gac	Tetr	Ola	Tru	Dre
Sma-USC206	4	-	10	14	-
SmaSNP62	4	1	-	un	9
Sma-E100	4	1	10	14	-
B11-I12/6/3	4	-	10	-	-
Sma-USC135	4	un	10	14un	-
Sma-USC272	4	1	10	14	-
Sma-USC37	-	-	-	14	-
Sma-E78	4	-	10	14un	14
Sma-E194	4	20	uc115	14	-

LG08	Gac	Tetr	Ola	Tru	Dre
Sma-USC194	7	-	-	-	-
Sma-E218	7	7	-	15	18
SMAC08	7	7	14	15	5
SmaUSC-E43	7	7	14	15un	5
SmaSNP147	7	-	-	-	-
Sma-USC269	7	7	14	15	-
Sma-USC170	14	4	12	6	21
Sma-USC18	7	-	-	8	-

LG09	Gac	Tetr	Ola	Tru	Dre
SmaSNP61	-	18	-	-	-
Sma-USC216	9	-	1	17	-
Sma-USC150	9	-	-	-	-
Sma-USC118	21	-	-	-	-
SmaUSC-E16	9	-	1	17	-
Sma-E99	-	-	-	-	1
SmaSNP100	9	18	-	17	1
SmaUSC-E36	9	18	1	17	-
Sma-USC126	9	18	1	17	-
SmaUSC-E23	9	18	1	-	-
Sma-E71	9	18	1	17	1
SmaSNP35	9	un	-	-	-
Sma-E197	9	un	-	-	3
Sma-E139	18	14	24	16	20
SmaUSC-E5	-	-	1	-	-
Sma-E302	9	18	1	17	-
F12-ITG16	-	un	-	-	-
SmaUSC-E41	9	18	1	17un	3
Sma-USC57	1	-	-	-	-
Sma-E248	-	un	1	un	19
SmaUSC-E2	9	-	-	-	-
Sma-USC21	9	18	1	17	-
Sma-E117	9	un	-	17	-
SMAC05	9	un	1	17	1
Sma-USC226	9	-	-	-	-

LG10	Gac	Tetr	Ola	Tru	Dre
SmaSNP64	12	-	7	3	23
Sma-USC175	12	-	-	-	-
Sma-USC217	sc68	9	7	3	-
SmaUSC-E20	sc68	-	7	-	-
SmaUSC-E27	12	9	-	3	-
SmaUSC-E32	sc114	un	uc90	-	-
Sma-USC162	sc180	-	7	-	23
SmaSNP157	12	un	7	-	-
Sma-USC244	sc114	un	uc90	3	-
Sma-USC113	-	9	7	3	-
Sma-USC96	12	-	-	-	23
Sma-E144	-	5	-	-	-
Sma-E290	12	un	7	un	-
Sma-E224	12	un	7	un	-
Sma-USC79	-	un	7	un	8
SmaSNP3	12	-	7	3	8

LG11	Gac	Tetr	Ola	Tru	Dre
SmaSNP87	sc27	-	sc1179	19	-
Sma-USC201	sc27	-	5	19	-
Sma-USC152	sc27	-	-	-	-
Sma-USC158	17	-	-	19	-
SmaUSC-E24	17	-	-	19	-
Sma-USC62	-	-	-	19	-
Sma-E96	17	-	-	-	-
Sma-USC22	17	-	-	-	-
Sma-E156	17	-	-	-	-
Sma-USC235	17	un	5	-	-

LG12	Gac	Tetr	Ola	Tru	Dre
Sma-USC169	13	-	-	-	-
SmaUSC-E25	13	12	-	21	5
Sma-USC60	13	-	-	-	-
SmaSNP140	13	un	9	un	-
3/9CA15	13	-	-	21	-
4/4CA4/13	-	-	9	-	-
Sma-USC89	13	-	9	21	-
Sma-USC20	-	-	-	-	5
SmaUSC-E14	13	un	-	21	-
Sma-USC19	13	-	9	-	-
Sma-USC133	-	-	9	-	-
Sma-USC56	13	-	-	-	-
SmaSNP73	-	12	-	21	21
SmaUSC-E21	13	-	-	21	-
SmaUSC-E22	13	-	-	21	-
Sma-E310	13	-	-	21	-
SmaSNP126	13	-	-	21	-
Sma-USC266	13	12	9	21	5

LG13	Gac	Tetr	Ola	Tru	Dre
Sma-USC280	-	un	-	5	-
Sma-E82	11	un	8	5	-
Sma-USC76	sc151	-	8	-	-
SmaSNP150	11	3	8	-	-
Sma-USC16	-	-	8	-	-
Sma-E120	11	3	8	5	3
Sma-E215	11	-	-	1	12
SmaUSC-E10	11	2	-	1	-
SmaUSC-E38	11	3	8	5	-
Sma-USC155	11	-	-	5	-
SmaSNP44	-	-	-	-	3
SmaSNP192	11	3	-	5	3

LG14	Gac	Tetr	Ola	Tru	Dre
Sma-E50	14	-	-	6	-
SmaSNP200	14	4	12	6un	-
SmaSNP32	-	-	12	6	5
SmaSNP45	14	4	12	-	-
SmaUSC-E28	14	-	-	-	10
Sma-USC85	14	-	12	-	-
Sma-USC82	-	-	-	6	-
Sma-E164	20	un	16	7	-

LG15	Gac	Tetr	Ola	Tru	Dre
Sma-USC78	-	16	-	11	-
Sma-E61	21	-	8	-	3
Sma-USC214	1	10	13	-	-
Sma-E86	1	-	-	-	-
Sma-USC211	1	-	-	-	-
SmaSNP188	1	un	13	11	18
Sma-USC149	-	-	13	-	-

LG16	Gac	Tetr	Ola	Tru	Dre
Sma-E279	-	un	-	8un	-
Sma-E137	7	un	18	8un	-
Sma-USC128	4	un	23	18	4
Sma-USC136	4	19	23	-	-
3/20CA17	4	19	23	18	-
Sma-USC282	4	-	23	18	-
Sma-USC285	4	19	23	18	-
Sma-USC223	4	19	23	18	-
Sma-E183	4	19	23	18	4

LG17	Gac	Tetr	Ola	Tru	Dre
Sma-USC91	21	-	-	10	-
Sma-E112	21	-	20	-	-
Smax-02	21	-	20	10	-
Sma-USC138	21	un	-	10	-
Sma-USC55	21	-	-	10un	12
Sma-E159	sc37	un	uc236	un	-
Sma-USC134	21	6	-	10	-
Sma-USC142	sc67	-	-	10	-
Sma-E184	sc126	-	20	10	-
SmaUSC-E1	sc126	un	20	10	24

LG18	Gac	Tetr	Ola	Tru	Dre
SmaUSC-E40	7	7	uc170	-	-
SmaUSC-E13	7	7	uc170	-	-
Sma-E195	7	7	uc170	-	-
Sma-USC160	-	7	-	15un	7
SmaUSC-E19	7	un	14	15	-

LG19	Gac	Tetr	Ola	Tru	Dre
3/20CA17	4	19	23	18	-
SmaSNP101	-	-	-	4	-
SmaSNP74	6	17	-	4	-
SmaSNP28	6	17	15	4	-
Sma-E142	6	un	15	4	-
Sma-USC108	6	-	-	-	-
Sma-E205	6	-	sc521	un	13
Sma-USC86	6	17	-	-	-
Sma-USC263	6	-	-	-	-
Smax-04b	6	17	-	4	-
Sma-USC23	-	-	uc72	4	-

LG20	Gac	Tetr	Ola	Tru	Dre
SmaSNP163	1	-	13	un	-
SmaSNP194	-	-	21	-	-
Sma-USC29	-	-	-	1	-
Sma-E189	-	2	-	1	1
Sma-E244	16	2	21	1	-
Sma-E270	-	2	21	1	-

LG21	Gac	Tetr	Ola	Tru	Dre
SmaSNP210	20	8	16	7	-
Sma-USC231	-	2	19	1	-
Sma-USC234	5	-	-	-	-
Sma-E316	5	-	19	1	12

LG22	Gac	Tetr	Ola	Tru	Dre
Sma-E91	1	un	sc2179	un	18
SmaSNP141	10	21un	11	12	15
Sma-E167	10	un	11	-	16
SmaUSC-E39	10	21un	-	12	-
Sma-USC58	10	-	-	-	-
SmaSNP160	10	-	-	-	-

LG23	Gac	Tetr	Ola	Tru	Dre
Sma-E168	18	14	24	16	20
Sma-USC273	18	14	24	16	-
Sma-USC38	18	14	-	16	-
Sma-USC54	-	-	24	-	-
SmaSNP68	18	-	-	-	-
Sma-E127	18	14	24	16	-
Sma-E154	-	-	24	-	-
SmaUSC-E33	-	14	-	16	-
SmaUSC-E31	18	-	-	16	20

FIGURE S3. Comparative mapping between the turbot map and the five model teleost genomes. Turbot linkage groups: LG1-LG24; stickleback (Gac), Tetraodon (Tni), Medaka (Ola), Fugu (Tru) and zebrafish (Dre). Markers yielding significant matches against at least one model fish genome are ordered on the left of each turbot linkage group (framework markers in boldface, markers at LOD<3.0 in normal type and accessory markers in italic type; see Figure 1 and Table S1). Details on similarity matches against each model genome are recorded as either the number of the specific chromosome or as the code of the unallocated genomic regions of the model species: (sc) stickleback or medaka scaffolds, (uc) medaka ultracontigs, (un) unrandom genomic or unassigned chromosomal sequences of *Tetraodon* and fugu. The significant unique hits under $E \leq 10^{-5}$ threshold are presented in boldface type; the remaining at $E < 10^{-10}$ in italic type.
