# Supplementary material for: An Expressed Sequence Tag (EST)-enriched genetic map of turbot (Scophthalmus maximus): a useful framework for comparative genomics across model and farmed teleosts
Source: BMC Genet. 2012 Jul 2;13:54. doi: 10.1186/1471-2156-13-54 (PMC3464660; doi:10.1186/1471-2156-13-54)
Supplement: Additional file 4 — Table S3. Number of markers and map length for each linkage group (LG) of turbot. [file 1471-2156-13-54-S4.pdf]

| Turbot \ Zebrafish | 2 | 16 | 19 | 7 | 18 | 25 | 9 | 5 | 1 | 23 | 8 | 3 | 6 | 4 | 12 | 10 | 11 | 15 | 20 | 13 | 14 | 17 | 21 | 22 | 24 | UN |
|---|---|---|---|---|---|---|---|---|---|---|---|---|---|---|---|---|---|---|---|---|---|---|---|---|---|---|
| 1 | 2 | | 2 | | | 1 | | | | | | | | | | | | | | | | | | 1 | | |
| 2 | | 2 | | 2 | | | | | | | | | | | | | | | | | | | | | | |
| 3 | | | | | | | | | | | | | | | | | | | | | | | | | | |
| 4 | | | | 1 | 1 | | | | | | | | | | | | | | | | | | | | | 1 |
| 5 | | | | | | | | | | | 1 | | | | | | | | | | | | | | | |
| 6 | | | | | 1 | 3 | | | | | | | 1 | | | | | | | | | | | | | |
| 7 | | | | | | | 1 | | | | | | | | | | | | | | 1 | | | | | |
| 8 | | | | | 1 | | | 2 | | | | | | | | | | | | | | | 1 | | | |
| 9 | | | 1 | | | | | | 4 | | | 2 | | | | | | | 1 | | | | | | | |
| 10 | | | | | | | | | | 3 | 2 | | | | | | | | | | | | | | | |
| 11 | | | | | | | | | | | | | | | | | | | | | | | | | | |
| 12 | | | | | | | | 3 | | | | | | | | | | | | | | | 1 | | | |
| 13 | | | | | | | | | | | | 3 | | | 1 | | | | | | | | | | | |
| 14 | | | | | | | | 1 | | | | | | | | 1 | | | | | | | | | | |
| 15 | | | | | 1 | | | | | | | 1 | | | | | | | | | | | | | | |
| 16 | | | | | | | | | | | | | | 2 | | | | | | | | | | | | |
| 17 | | | | | | | | | | | | | | | 1 | | | | | | | | | | 1 | |
| 18 | | | | 1 | | | | | | | | | | | | | | | | | | | | | | |
| 19 | | | | | | | | | | | | | | | | | | | | 1 | | | | | | |
| 20 | | | | | | | | | 1 | | | | | | | | | | | | | | | | | |
| 21 | | | | | | | | | | | | | | | 1 | | | | | | | | | | | |
| 22 | | 1 | | | 1 | | | | | | | | | | | | | 1 | | | | | | | | |
| 23 | | | | | | | | | | | | | | | | | | | 2 | | | | | | | |
| UL | | | 1 | 1 | | | 1 | | | | 1 | | | | 1 | 1 | | | | | | | | | | |

**FIGURE S4. Oxford grid showing syntenies between the turbot linkage map and the zebrafish genome**. In gray background syntenies with two or more significant hits. UL: unlinked markers in the turbot map; UN: unrandom genomic regions of the model fish species.
