# Supplementary material for: An Expressed Sequence Tag (EST)-enriched genetic map of turbot (Scophthalmus maximus): a useful framework for comparative genomics across model and farmed teleosts
Source: BMC Genet. 2012 Jul 2;13:54. doi: 10.1186/1471-2156-13-54 (PMC3464660; doi:10.1186/1471-2156-13-54)
Supplement: Additional file 6 — Figure S3. Comparative mapping between the turbot map and the five model teleost genomes. [file 1471-2156-13-54-S6.docx]

**Table S2 - Informative markers used to construct the turbot consensus map**

| Mapping population | Total  markers | New markers | Common markers with HF | Common markers with DF | Common markers with QF set*^a^* |
| --- | --- | --- | --- | --- | --- |
| HF | 181 | 0 | - | 90 | 69 |
| DF | 281 | 133 | 90 | - | 66 |
| QF1 | 106 | 12 | 69 | 59 | 94 |
| QF2 | 119 | 22 | 67 | 64 | 97 |
| QF3 | 113 | 27 | 63 | 66 | 86 |
| QF4 | 101 | 14 | 64 | 59 | 87 |
| QF5 | 87 | 5 | 59 | 56 | 82 |
| QF6 | 116 | 26 | 63 | 66 | 90 |
| QF7 | 88 | 1 | 61 | 57 | 87 |

*^a^*QF set refers to the set of 99 homogeneously distributed markers in the previous turbot map used for QTL location (Martínez et al., 2009).
