# Supplementary material for: An Expressed Sequence Tag (EST)-enriched genetic map of turbot (Scophthalmus maximus): a useful framework for comparative genomics across model and farmed teleosts
Source: BMC Genet. 2012 Jul 2;13:54. doi: 10.1186/1471-2156-13-54 (PMC3464660; doi:10.1186/1471-2156-13-54)
Supplement: Additional file 7 — Figure S4. Oxford grid showing syntenies between the turbot linkage map and the zebrafish genome. [file 1471-2156-13-54-S7.docx]

**Table S3 - Number of markers and map length for each linkage group (LG) of turbot consensus, paternal and maternal maps**

| Consensus map | | | | | | |  | Paternal map | | | | | |  | Maternal map | | | | | |  |
| --- | --- | --- | --- | --- | --- | --- | --- | --- | --- | --- | --- | --- | --- | --- | --- | --- | --- | --- | --- | --- | --- |
| LG | No. marker | No. fram. | No. LOD<3 | No. acces. | Total length | Fram. leng. |  | No. marker | No. fram. | No. LOD<3 | Total length | Fram. leng. | CML |  | No. marker | No. fram. | No. LOD<3 | Total length | Fram. leng. | CML | F:M ratio |
| LG1 | 24 | 16 | 7 | 1 | 98,5 | 78,3 |  | 14 | 12 | 2 | 77,5 | 45,5 | 56,6 |  | 10 | 10 | 0 | 99,6 | 99,6 | 95,4 | 1,69 |
| LG2 | 35 | 19 | 11 | 5 | 85,7 | 56,7 |  | 14 | 14 | 0 | 30,1 | 30,1 | 29,8 |  | 13 | 12 | 1 | 85 | 82,3 | 82,3 | 2,73 |
| LG3 | 17 | 12 | 5 | 0 | 76,5 | 63,1 |  | 12 | 12 | 0 | 54,5 | 54,5 | 54,5 |  | 14 | 14 | 0 | 89,2 | 89,2 | 89,2 | 1,64 |
| LG4 | 17 | 16 | 0 | 1 | 63,7 | 63,7 |  | 13 | 13 | 0 | 64,4 | 64,4 | 64,4 |  | 9 | 4 | 5 | 88,5 | 56,7 | 83,4 | 1,30 |
| LG5 | 20 | 16 | 3 | 1 | 91,9 | 55,3 |  | 9 | 9 | 0 | 39 | 39 | 39 |  | 14 | 14 | 0 | 111,1 | 111,1 | 81 | 2,08 |
| LG6 | 19 | 13 | 4 | 2 | 90,1 | 46,8 |  | 9 | 5 | 4 | 67,1 | 26,6 | 26,6 |  | 12 | 12 | 0 | 49,2 | 49,2 | 40,7 | 1,53 |
| LG7 | 18 | 10 | 7 | 1 | 43,5 | 26,2 |  | 5 | 5 | 0 | 19,2 | 19,2 | 19,2 |  | 8 | 8 | 0 | 44,5 | 44,5 | 44,5 | 2,32 |
| LG8 | 14 | 13 | 1 | 0 | 47,8 | 47,8 |  | 11 | 11 | 0 | 50,6 | 50,6 | 8,2 |  | 6 | 6 | 0 | 26,4 | 26,4 | 26,4 | 3,22 |
| LG9 | 29 | 25 | 2 | 2 | 70,3 | 70,3 |  | 18 | 18 | 0 | 48,6 | 48,6 | 44,7 |  | 11 | 5 | 6 | 80,6 | 42,3 | 80,6 | 1,80 |
| LG10 | 24 | 19 | 3 | 2 | 67,3 | 57,2 |  | 14 | 14 | 0 | 42,4 | 42,4 | 41,3 |  | 12 | 11 | 1 | 66,5 | 57,3 | 57,3 | 1,39 |
| LG11 | 20 | 15 | 4 | 1 | 52,6 | 51,7 |  | 6 | 6 | 0 | 37,6 | 37,6 | 37,6 |  | 8 | 8 | 0 | 61,5 | 61,5 | 52,4 | 1,39 |
| LG12 | 23 | 19 | 2 | 2 | 60,3 | 60,3 |  | 13 | 13 | 0 | 45,9 | 45,9 | 45,9 |  | 19 | 19 | 0 | 75,2 | 75,2 | 75,2 | 1,64 |
| LG13 | 24 | 18 | 5 | 1 | 77,7 | 77,7 |  | 15 | 15 | 0 | 39,7 | 39,7 | 24,6 |  | 11 | 10 | 1 | 79,4 | 70,4 | 70,4 | 2,86 |
| LG14 | 18 | 14 | 0 | 4 | 60,7 | 60,7 |  | 8 | 8 | 0 | 19,4 | 19,4 | 19,4 |  | 13 | 13 | 0 | 67,9 | 67,9 | 38,1 | 1,96 |
| LG15 | 21 | 20 | 0 | 1 | 52,4 | 52,4 |  | 15 | 15 | 0 | 63,3 | 63,3 | 47,9 |  | 14 | 12 | 2 | 48,9 | 44,8 | 44,5 | 0,93 |
| LG16 | 22 | 16 | 2 | 4 | 66,5 | 66,5 |  | 14 | 14 | 0 | 65,7 | 65,7 | 36,8 |  | 10 | 10 | 0 | 59,9 | 59,9 | 50 | 1,36 |
| LG17 | 15 | 14 | 1 | 0 | 67,1 | 55,8 |  | 3 | 3 | 0 | 11,6 | 11,6 | 11,6 |  | 12 | 12 | 0 | 58,3 | 58,3 | 29,4 | 2,53 |
| LG18 | 9 | 8 | 0 | 1 | 25,9 | 25,9 |  | 3 | 3 | 0 | 8,5 | 8,5 | 8,5 |  | 8 | 8 | 0 | 18,4 | 18,4 | 11,4 | 1,34 |
| LG19 | 16 | 10 | 3 | 3 | 38,1 | 38,1 |  | 3 | 3 | 0 | 8 | 8 | - |  | 9 | 9 | 0 | 35,5 | 35,5 | - | - |
| LG20 | 12 | 10 | 0 | 2 | 35,6 | 35,6 |  | 8 | 8 | 0 | 34,5 | 34,5 | 31 |  | 3 | 3 | 0 | 25,4 | 25,4 | 25,4 | 0,82 |
| LG21 | 9 | 9 | 0 | 0 | 24,2 | 24,2 |  | 5 | 5 | 0 | 1,8 | 1,8 | 1,8 |  | 8 | 8 | 0 | 46,9 | 46,9 | 41,8 | 23,22 |
| LG22 | 15 | 11 | 1 | 3 | 38,9 | 25,5 |  | 2 | 2 | 0 | 5,8 | 5,8 | - |  | 11 | 11 | 0 | 22,2 | 22,2 | - | - |
| LG23 | 14 | 10 | 2 | 2 | 60,7 | 46,9 |  | 6 | 6 | 0 | 19 | 19 | - |  | 3 | 3 | 0 | 29 | 29 | - | - |
| LG24 | 3 | 3 | 0 | 0 | 6,7 | 6,7 |  | 1 | 1 | 0 | 0 | 0 | - |  | 3 | 3 | 0 | 0 | 0 | - | - |
| Total | 438 | 336 | 63 | 39 | 1402,7 | 1193,4 |  | 221 | 215 | 6 | 854,2 | 781,7 | 649,4 |  | 241 | 225 | 16 | 1369,1 | 1274 | 1119,4 |  |
| Range | 3-29 | 3-25 | 0-11 | 0-5 | 6,7-98,5 | 6,7-78,3 |  | 1-17 | 1-17 | 0-4 | 0-77,5 | 0-65,7 | 1,8-64,4 |  | 3-19 | 3-19 | 0-6 | 0-111,1 | 0-111,1 | 11,4-95,4 |  |
| Mean | 18,3 | 14 | 2,6 | 1,6 | 58,4 | 49,7 |  | 9,2 | 8,9 | 0,3 | 35,6 | 32,6 | 32,5 |  | 10,0 | 9,4 | 0,7 | 57,0 | 53,1 | 56,0 | 1,60 |

LG: Linkage group; No. fram: Number of framework markers; No. LOD<3: Number of markers linked at LOD score <3; Fram. leng.: Framework length; CML: common marker length between male and female maps; F:M ratio: ratio between female and male LG length; The global F:M ratio was obtained by dividing the total length of female and male maps.
