# Supplementary material for: An Expressed Sequence Tag (EST)-enriched genetic map of turbot (Scophthalmus maximus): a useful framework for comparative genomics across model and farmed teleosts
Source: BMC Genet. 2012 Jul 2;13:54. doi: 10.1186/1471-2156-13-54 (PMC3464660; doi:10.1186/1471-2156-13-54)
Supplement: Additional file 8 — Table S4. Putative syntenic markers (210) between the turbot genetic map and the stickleback genome. [file 1471-2156-13-54-S8.docx]

| **Table S4. Putative syntenic markers (210) between the turbot and the stickleback genomes** | | | | | | | |
| --- | --- | --- | --- | --- | --- | --- | --- |
| **Loci** | **Turbot LG***^a^* | **Distance cM** | **Marker type***^b^* | **Stickleback Chromos***^c^* | **Stickleback location bp** | **Identity ID%** | **EValue** |
| SmaUSC-E42 | LG01 | 0 | LOD<3.0 | sc120 | 102967 | 85,63 | 3,00E-30 |
| Sma-USC268 | LG01 | 0,909 | LOD<3.0 | II | 15199485 | 89,74 | 3,00E-15 |
| Sma-USC1 | LG01 | 14,356 | Framework | XVIII | 2128742 | 91,11 | 1,00E-07 |
| Sma-E231 | LG01 | 15,717 | LOD<3.0 | III | 15207485 | 90,14 | 6,00E-39 |
| Sma-USC15 | LG01 | 44,802 | Framework | III | 965371 | 91,8 | 4,00E-31 |
| SmaUSC-E15 | LG01 | 51,104 | Framework | III | 8177188 | 89,23 | 1,00E+00 |
| SMAC09 | LG01 | 51,489 | Framework | III | 6654462 | 88,16 | 2,00E-32 |
| 1/4AC18 | LG01 | 52,263 | Framework | III | 6950778 | 93,75 | 9,00E-40 |
| Sma-USC271 | LG01 | 56,423 | Framework | III | 10111065 | 83,48 | 1,00E-12 |
| SmaSNP204 | LG01 | 60,345 | Framework | III | 2913770 | 88,03 | 1,00E-33 |
| Sma-E277 | LG01 | 61,515 | Framework | III | 4681066 | 88,41 | 3,00E-09 |
| SMAC07 | LG01 | 74,958 | Framework | III | 5110556 | 86,54 | 2,00E-32 |
| Sma-USC218 | LG01 | 80,053 | LOD<3.0 | sc111 | 171514 | 89,58 | 8,00E-23 |
| Sma-USC222 | LG01 | 91,456 | LOD<3.0 | X | 13910989 | 91,06 | 2,00E-34 |
| SmaSNP19 | LG01 | 97,592 | LOD<3.0 | X | 12196747 | 93,75 | 1,00E-140 |
| SmaSNP153 | LG01 | 98,446 | LOD<3.0 | X | 12196747 | 93,75 | 1,00E-140 |
| SmaSNP143 | LG02 | 0 | Accessory | XX | 10546376 | 84,24 | 1,00E-21 |
| SmaSNP145 | LG02 | 0 | Accessory | XX | 5186095 | 91,38 | 2,00E-32 |
| SmaSNP40 | LG02 | 0 | Accessory | VI | 8107715 | 93,33 | 1,00E-13 |
| SmaUSC-E17 | LG02 | 0 | Accessory | VI | 9296943 | 92,59 | 3,00E-12 |
| Sma-USC46 | LG02 | 9,183 | Framework | XX | 6052607 | 81,82 | 1,00E-19 |
| SmaSNP103 | LG02 | 10,087 | LOD<3.0 | I | 27451764 | 89,34 | 4,00E-31 |
| Sma-USC242 | LG02 | 18,018 | Framework | XX | 8192705 | 89,8 | 4,00E-07 |
| SMAC01 | LG02 | 29,772 | LOD<3.0 | sc47 | 767955 | 97,67 | 8,00E-13 |
| SmaSNP149 | LG02 | 30,533 | LOD<3.0 | I | 22244276 | 91,23 | 2,00E-11 |
| Sma-USC168 | LG02 | 31,248 | Framework | XX | 13001360 | 89,66 | 8,00E-10 |
| SmaSNP71 | LG02 | 32,48 | Framework | XX | 762961 | 90,63 | 3,00E-13 |
| SmaSNP30 | LG02 | 40,217 | Framework | XX | 821900 | 88,65 | 9,00E-50 |
| Sma-USC84 | LG02 | 40,994 | Framework | XX | 10996330 | 87,5 | 2,00E-39 |
| Sma-USC36 | LG02 | 50,3 | LOD<3.0 | XX | 12732792 | 92,68 | 8,00E-07 |
| Sma-USC187 | LG02 | 52,458 | Framework | I | 27734271 | 93,94 | 8,00E-47 |
| Sma-USC64 | LG02 | 54,994 | LOD<3.0 | XX | 8052773 | 87,91 | 1,00E-17 |
| Sma-USC43 | LG02 | 56,728 | Framework | sc74 | 21914 | 92,08 | 1,00E-30 |
| Sma-USC93 | LG03 | 0 | LOD<3.0 | XV | 308810 | 85,91 | 2,00E-28 |
| SmaUSC-E34 | LG03 | 36,826 | Framework | XV | 8879246 | 92,68 | 3,00E-19 |
| Sma-USC157 | LG03 | 37,987 | Framework | XV | 9346526 | 89,53 | 2,00E-19 |
| Sma-USC179 | LG03 | 46,632 | Framework | XV | 6320782 | 92,98 | 1,00E-11 |
| Sma-E118 | LG03 | 52,302 | Framework | XV | 5362730 | 87,73 | 2,00E-56 |
| Sma-E72 | LG03 | 65,946 | Framework | XV | 13239602 | 91,24 | 2,00E-42 |
| SmaSNP190 | LG04 | 0 | Accessory | II | 21371214 | 91,03 | 7,00E-45 |
| SmaSNP181 | LG04 | 19,13 | Framework | II | 10572988 | 89,94 | 1,00E-49 |
| Sma-USC100 | LG04 | 19,791 | Framework | II | 10507861 | 81,9 | 7,00E-23 |
| Sma-USC102 | LG04 | 19,889 | Framework | II | 10500045 | 92,86 | 2,00E-31 |
| SmaUSC-E30 | LG05 | 6,356 | Framework | VIII | 6067404 | 92,16 | 2,00E-10 |
| SmaSNP31 | LG05 | 11,676 | Framework | VIII | 6648386 | 88,18 | 4,00E-24 |
| Sma-USC12 | LG05 | 45,328 | Framework | VIII | 15372758 | 94,92 | 2,00E-17 |
| Sma-USC202 | LG05 | 48,915 | Framework | VIII | 16253281 | 93,02 | 5,00E-08 |
| Sma-USC265 | LG05 | 51,886 | Framework | VIII | 19294004 | 93,18 | 2,00E-08 |
| Sma-USC88 | LG05 | 52,592 | Framework | VIII | 16665783 | 94,68 | 2,00E-31 |
| Sma-USC225 | LG05 | 54,627 | LOD<3.0 | VIII | 8859679 | 87,5 | 6,00E-23 |
| SmaSNP29 | LG06 | 0 | Framework | XIX | 11017496 | 91,53 | 4,00E-64 |
| SmaUSC-E7 | LG06 | 0 | Accessory | XIX | 8761129 | 90,74 | 5,00E-30 |
| Sma-USC188 | LG06 | 8,771 | Framework | XIX | 8593443 | 87,64 | 1,00E-14 |
| Sma-USC107 | LG06 | 21,603 | Framework | XIX | 11798147 | 94,44 | 2,00E-14 |
| Sma-USC110 | LG06 | 37,626 | Framework | XIX | 17112240 | 92,53 | 3,00E-83 |
| SmaUSC-E29 | LG06 | 40,181 | Framework | XIX | 11926555 | 91,67 | 6,00E-35 |
| Sma-E315 | LG06 | 40,217 | Framework | XIX | 11926555 | 91,67 | 6,00E-35 |
| Sma-USC264 | LG06 | 87,238 | LOD<3.0 | XIX | 3021832 | 95,45 | 4,00E-11 |
| Sma-USC206 | LG07 | 5,698 | Framework | IV | 10281338 | 100 | 1,00E-44 |
| SmaSNP62 | LG07 | 7,156 | Framework | IV | 12142535 | 87,56 | 1,00E-49 |
| Sma-E100 | LG07 | 14,066 | Framework | IV | 15512412 | 88,31 | 8,00E-14 |
| B11-I12/6/3 | LG07 | 19,2 | Framework | IV | 14780141 | 88,76 | 8,00E-18 |
| Sma-USC135 | LG07 | 25,008 | Framework | IV | 17753147 | 90,98 | 6,00E-36 |
| Sma-USC272 | LG07 | 27,086 | Framework | IV | 15613026 | 97,01 | 2,00E-22 |
| Sma-E78 | LG07 | 33,698 | LOD<3.0 | IV | 3868520 | 96,5 | 9,00E-63 |
| Sma-E194 | LG07 | 43,478 | LOD<3.0 | IV | 2044922 | 89,1 | 4,00E-42 |
| Sma-USC194 | LG08 | 3,735 | Framework | VII | 16458503 | 92,21 | 3,00E-21 |
| Sma-E218 | LG08 | 10,08 | Framework | VII | 17319023 | 91,53 | 8,00E-13 |
| SMAC08 | LG08 | 12,617 | Framework | VII | 17908022 | 92,66 | 2,00E-35 |
| SmaUSC-E43 | LG08 | 14,268 | Framework | VII | 14937017 | 96,91 | 2,00E-40 |
| SmaSNP147 | LG08 | 25,604 | Framework | VII | 12193057 | 87,16 | 5,00E-21 |
| Sma-USC269 | LG08 | 29,395 | LOD<3.0 | VII | 18830763 | 88,24 | 5,00E-22 |
| Sma-USC170 | LG08 | 29,442 | Framework | XIV | 735843 | 91,09 | 5,00E-26 |
| Sma-USC18 | LG08 | 39,598 | Framework | VII | 7949694 | 90,32 | 4,00E-12 |
| Sma-USC216 | LG09 | 0 | Framework | IX | 4915563 | 85,48 | 2,00E-20 |
| Sma-USC150 | LG09 | 2,683 | Framework | IX | 5048847 | 84,34 | 6,00E-08 |
| Sma-USC118 | LG09 | 8,569 | Framework | XXI | 749251 | 93,88 | 9,00E-12 |
| SmaUSC-E16 | LG09 | 9,355 | Framework | IX | 4421181 | 90,2 | 1,00E-39 |
| SmaSNP100 | LG09 | 19,471 | LOD<3.0 | IX | 5485091 | 93,33 | 1,00E-61 |
| SmaUSC-E36 | LG09 | 21,202 | LOD<3.0 | IX | 5320522 | 90,4 | 2,00E-35 |
| Sma-USC126 | LG09 | 26,947 | Framework | IX | 8463766 | 89,88 | 1,00E-76 |
| SmaUSC-E23 | LG09 | 29,277 | Framework | IX | 8909337 | 88,54 | 1,00E-20 |
| Sma-E71 | LG09 | 31,768 | Framework | IX | 1724967 | 87,62 | 4,00E-50 |
| SmaSNP35 | LG09 | 32,383 | Framework | IX | 9801341 | 85,71 | 6,00E-33 |
| Sma-E197 | LG09 | 35,808 | Framework | IX | 803188 | 88,76 | 1,00E-16 |
| Sma-E139 | LG09 | 37,583 | Framework | XVIII | 2150149 | 86,83 | 5,00E-40 |
| Sma-E302 | LG09 | 38,104 | Framework | IX | 17521129 | 82,95 | 8,00E-14 |
| SmaUSC-E41 | LG09 | 40,111 | Framework | IX | 12938250 | 88,79 | 7,00E-63 |
| Sma-USC57 | LG09 | 48,932 | Framework | I | 16271116 | 90,7 | 9,00E-22 |
| SmaUSC-E2 | LG09 | 50,21 | Framework | IX | 10923704 | 94,39 | 5,00E-39 |
| Sma-USC21 | LG09 | 52,331 | Framework | IX | 15948559 | 92,39 | 1,00E-63 |
| Sma-E117 | LG09 | 58,577 | Framework | IX | 4162838 | 92 | 5,00E-30 |
| SMAC05 | LG09 | 63,506 | Framework | IX | 12244245 | 92,66 | 4,00E-64 |
| Sma-USC226 | LG09 | 70,308 | Framework | IX | 3367593 | 91,11 | 5,00E-07 |
| SmaSNP64 | LG10 | 0 | Accessory | XII | 2989163 | 87,4 | 9,00E-26 |
| Sma-USC175 | LG10 | 0 | LOD<3.0 | XII | 1724267 | 91,3 | 2,00E-07 |
| Sma-USC217 | LG10 | 10,065 | Framework | sc68 | 560174 | 92,91 | 1,00E-43 |
| SmaUSC-E20 | LG10 | 11,75 | Framework | sc68 | 742111 | 87,91 | 2,00E-10 |
| SmaUSC-E27 | LG10 | 26,545 | Framework | XII | 11326508 | 89,47 | 2,00E-40 |
| SmaUSC-E32 | LG10 | 32,175 | Framework | sc114 | 351303 | 85,23 | 1,00E-24 |
| Sma-USC162 | LG10 | 35,119 | Framework | sc180 | 66965 | 91,59 | 6,00E-32 |
| SmaSNP157 | LG10 | 43,409 | Framework | XII | 6439207 | 88,42 | 7,00E-20 |
| Sma-USC244 | LG10 | 43,512 | Framework | sc114 | 210310 | 88,16 | 5,00E-16 |
| Sma-USC96 | LG10 | 61,152 | Framework | XII | 17761067 | 87,72 | 9,00E-07 |
| Sma-E290 | LG10 | 62,138 | Framework | XII | 17315740 | 86,39 | 3,00E-29 |
| Sma-E224 | LG10 | 62,182 | Framework | XII | 17315740 | 86,39 | 3,00E-29 |
| SmaSNP3 | LG10 | 64,36 | LOD<3.0 | XII | 8667481 | 88,54 | 4,00E-18 |
| SmaSNP87 | LG11 | 0 | Accessory | sc27 | 1851837 | 89,93 | 6,00E-39 |
| Sma-USC201 | LG11 | 6,989 | Framework | sc27 | 3630667 | 92 | 6,00E-10 |
| Sma-USC152 | LG11 | 7,041 | Framework | sc27 | 3689382 | 83,16 | 6,00E-20 |
| Sma-USC158 | LG11 | 28,775 | Framework | XVII | 9292607 | 86,03 | 2,00E-32 |
| SmaUSC-E24 | LG11 | 34,751 | Framework | XVII | 14125620 | 89,12 | 3,00E-39 |
| Sma-E96 | LG11 | 41,48 | Framework | XVII | 12538788 | 83,7 | 9,00E-09 |
| Sma-USC22 | LG11 | 42,957 | Framework | XVII | 11125262 | 89,71 | 4,00E-14 |
| Sma-E156 | LG11 | 45,997 | LOD<3.0 | XVII | 1508101 | 93,33 | 8,00E-20 |
| Sma-USC235 | LG11 | 50,685 | Framework | XVII | 12520408 | 91,3 | 1,00E-16 |
| Sma-USC169 | LG12 | 0 | Accessory | XIII | 13634306 | 88,61 | 8,00E-13 |
| Sma-USC60 | LG12 | 0 | Framework | XIII | 12581336 | 98 | 5,00E-17 |
| SmaUSC-E25 | LG12 | 0 | Accessory | XIII | 16021709 | 89,86 | 2,00E-62 |
| SmaSNP140 | LG12 | 2,091 | Framework | XIII | 13154659 | 79,37 | 1,00E-40 |
| 3/9CA15 | LG12 | 6,375 | Framework | XIII | 11184756 | 95,56 | 1,00E-11 |
| Sma-USC89 | LG12 | 15,221 | Framework | XIII | 9664432 | 92,55 | 7,00E-29 |
| SmaUSC-E14 | LG12 | 24,234 | Framework | XIII | 4948852 | 95,92 | 3,00E-09 |
| Sma-USC19 | LG12 | 24,745 | Framework | XIII | 5242534 | 83,78 | 3,00E-11 |
| Sma-USC56 | LG12 | 26,761 | Framework | XIII | 6017804 | 82,61 | 9,00E-08 |
| SmaUSC-E21 | LG12 | 28,132 | Framework | XIII | 1681952 | 85 | 5,00E-11 |
| SmaUSC-E22 | LG12 | 31,575 | Framework | XIII | 17356870 | 87,3 | 1,00E-05 |
| Sma-E310 | LG12 | 31,91 | LOD<3.0 | XIII | 17356870 | 87,3 | 1,00E-05 |
| SmaSNP126 | LG12 | 32,183 | Framework | XIII | 17356697 | 93,18 | 1,00E-06 |
| Sma-USC266 | LG12 | 60,323 | Framework | XIII | 18844207 | 87,69 | 1,00E-09 |
| Sma-E82 | LG13 | 12,364 | Framework | XI | 2515950 | 91,74 | 1,00E-37 |
| Sma-USC76 | LG13 | 24,955 | Framework | sc151 | 94128 | 79,86 | 6,00E-12 |
| SmaSNP150 | LG13 | 25,35 | LOD<3.0 | XI | 10897923 | 89,13 | 2,00E-20 |
| Sma-E120 | LG13 | 47,161 | Framework | XI | 14085801 | 92,5 | 2,00E-39 |
| Sma-E215 | LG13 | 49,454 | Framework | XI | 13655553 | 84,85 | 9,00E-13 |
| SmaUSC-E10 | LG13 | 49,493 | Framework | XI | 13655553 | 93,33 | 1,00E-35 |
| SmaUSC-E38 | LG13 | 58,91 | Framework | XI | 5665417 | 83,24 | 1,00E-24 |
| Sma-USC155 | LG13 | 66,527 | Framework | XI | 6367015 | 87,5 | 2,00E-18 |
| SmaSNP192 | LG13 | 77,691 | Framework | XI | 15680352 | 93,51 | 1,00E-105 |
| Sma-E50 | LG14 | 0 | Accessory | XIV | 1628264 | 90,91 | 1,00E-14 |
| SmaSNP200 | LG14 | 0 | Accessory | XIV | 7786674 | 85,89 | 8,00E-32 |
| SmaSNP45 | LG14 | 0 | Accessory | XIV | 1643397 | 85,71 | 3,00E-07 |
| SmaUSC-E28 | LG14 | 4,039 | Framework | XIV | 10227008 | 91,8 | 3,00E-09 |
| Sma-USC85 | LG14 | 6,461 | Framework | XIV | 13235833 | 83,93 | 2,00E-08 |
| Sma-E164 | LG14 | 60,662 | Framework | XX | 14453206 | 93,33 | 1,00E-15 |
| Sma-E61 | LG15 | 0,85 | Framework | XI | 9137150 | 85,82 | 6,00E-24 |
| Sma-USC214 | LG15 | 8,793 | Framework | I | 7788177 | 89,08 | 2,00E-29 |
| Sma-E86 | LG15 | 10,183 | Framework | I | 8599243 | 83,18 | 3,00E-10 |
| Sma-USC211 | LG15 | 31,22 | Framework | I | 1825782 | 90,91 | 1,00E-06 |
| SmaSNP188 | LG15 | 50,397 | Framework | I | 17446082 | 91,53 | 5,00E-64 |
| Sma-E137 | LG16 | 1,25 | Framework | VII | 536758 | 85,71 | 4,00E-25 |
| Sma-USC128 | LG16 | 23,711 | Framework | IV | 24230503 | 93,13 | 1,00E-58 |
| Sma-USC136 | LG16 | 41,027 | Framework | IV | 18723081 | 87,18 | 4,00E-21 |
| 3/20CA17 | LG16 | 43,462 | Framework | IV | 17827523 | 88,98 | 1,00E-26 |
| Sma-USC282 | LG16 | 45,947 | Framework | IV | 28535139 | 85,85 | 3,00E-12 |
| Sma-USC285 | LG16 | 47,255 | Framework | IV | 28508290 | 87,32 | 1,00E-33 |
| Sma-USC223 | LG16 | 54,141 | Framework | IV | 30161452 | 94,17 | 2,00E-36 |
| Sma-E183 | LG16 | 57,157 | Framework | IV | 30485838 | 93,21 | 1,00E-83 |
| Sma-USC91 | LG17 | 5,424 | Framework | XXI | 7690916 | 94,52 | 1,00E-23 |
| Sma-E112 | LG17 | 6,707 | Framework | XXI | 8037685 | 86,02 | 7,00E-14 |
| Smax-02 | LG17 | 12,79 | Framework | XXI | 4951922 | 89,29 | 1,00E-18 |
| Sma-USC138 | LG17 | 18,343 | Framework | XXI | 1600188 | 84,83 | 9,00E-17 |
| Sma-USC55 | LG17 | 25,623 | Framework | XXI | 609079 | 85,29 | 2,00E-21 |
| Sma-E159 | LG17 | 33,482 | Framework | sc37 | 1418929 | 92,5 | 2,00E-06 |
| Sma-USC134 | LG17 | 47,294 | Framework | XXI | 11317340 | 88,24 | 2,00E-28 |
| Sma-USC142 | LG17 | 49,386 | Framework | sc67 | 82799 | 86,57 | 7,00E-08 |
| Sma-E184 | LG17 | 55,844 | Framework | sc126 | 131583 | 94,03 | 1,00E-17 |
| SmaUSC-E1 | LG17 | 55,844 | Framework | sc126 | 131871 | 92,45 | 1,00E-33 |
| SmaUSC-E40 | LG18 | 11,133 | Framework | VII | 26216616 | 91,38 | 3,00E-12 |
| SmaUSC-E13 | LG18 | 12,172 | Framework | VII | 26216598 | 89,47 | 9,00E-16 |
| Sma-E195 | LG18 | 12,245 | Framework | VII | 26216616 | 91,38 | 3,00E-12 |
| SmaUSC-E19 | LG18 | 25,892 | Framework | VII | 24174062 | 91,35 | 2,00E-23 |
| 3/20CA17 | LG19 | 0 | Framework | IV | 17827523 | 88,98 | 1,00E-26 |
| SmaSNP74 | LG19 | 0 | Accessory | VI | 8038851 | 80,97 | 2,00E-27 |
| SmaSNP28 | LG19 | 0 | Accessory | VI | 3639999 | 84,48 | 7,00E-29 |
| Sma-E142 | LG19 | 6,793 | LOD<3.0 | VI | 4067603 | 91,18 | 1,00E-15 |
| Sma-USC108 | LG19 | 10,596 | LOD<3.0 | VI | 2491218 | 85,54 | 2,00E-10 |
| Sma-E205 | LG19 | 14,021 | Framework | VI | 796902 | 93,91 | 5,00E-39 |
| Sma-USC86 | LG19 | 24,246 | Framework | VI | 16080080 | 93,06 | 9,00E-21 |
| Sma-USC263 | LG19 | 25,418 | LOD<3.0 | VI | 11648871 | 90,91 | 2,00E-10 |
| Smax-04b | LG19 | 33,571 | Framework | VI | 16674686 | 89,47 | 2,00E-09 |
| SmaSNP163 | LG20 | 0 | Accessory | I | 853307 | 92,86 | 2,00E-13 |
| Sma-E244 | LG20 | 19,871 | Framework | XVI | 2373553 | 90,83 | 1,00E-34 |
| SmaSNP210 | LG21 | 0 | Framework | XX | 10546376 | 84,24 | 1,00E-21 |
| Sma-USC234 | LG21 | 20,651 | Framework | V | 7147278 | 93,68 | 4,00E-66 |
| Sma-E316 | LG21 | 24,154 | Framework | V | 5595661 | 96,12 | 1,00E-54 |
| Sma-E167 | LG22 | 0 | Framework | X | 15420858 | 89,74 | 1,00E-71 |
| Sma-E91 | LG22 | 0 | Accessory | I | 424321 | 88,46 | 2,00E-39 |
| SmaSNP141 | LG22 | 0 | Accessory | X | 3210868 | 95,22 | 3,00E-90 |
| SmaUSC-E39 | LG22 | 8,999 | Framework | X | 3734034 | 87,77 | 5,00E-47 |
| Sma-USC58 | LG22 | 25,543 | Framework | X | 5983886 | 84,78 | 5,00E-07 |
| SmaSNP160 | LG22 | 38,861 | LOD<3.0 | X | 9235354 | 88,24 | 5,00E-11 |
| Sma-E168 | LG23 | 0,429 | Framework | XVIII | 8596787 | 90,63 | 1,00E-34 |
| Sma-USC273 | LG23 | 11,2 | Framework | XVIII | 2314947 | 91,03 | 5,00E-45 |
| Sma-USC38 | LG23 | 20,119 | Framework | XVIII | 10968753 | 90,67 | 1,00E-17 |
| SmaSNP68 | LG23 | 27,865 | Framework | XVIII | 15059642 | 85,14 | 2,00E-07 |
| Sma-E127 | LG23 | 28,875 | Framework | XVIII | 8941163 | 84,58 | 2,00E-38 |
| SmaUSC-E31 | LG23 | 49,841 | LOD<3.0 | XVIII | 6755580 | 86,64 | 4,00E-64 |
| F8-I11/8/17 | LG24 | 2,749 | Framework | II | 1405654 | 94,37 | 1,00E-22 |
| SmaSNP89 | UL | - | - | I | 23735780 | 95,51 | 1,00E-30 |
| SmaSNP196 | UL | - | - | III | 11467907 | 86,52 | 6,00E-14 |
| SmaSNP131 | UL | -. | - | VII | 24063617 | 87,3 | 1,00E-100 |
| SmaSNP88 | UL | - | - | VII | 699201 | 95,54 | 2,00E-66 |
| SmaSNP136 | UL | - | - | X | 15062542 | 95,05 | 7,00E-38 |
| Sma-E113 | UL | - | - | XI | 15558130 | 85,62 | 4,00E-28 |
| SmaSNP5 | UL | - | - | XI | 16247038 | 88,54 | 3,00E-40 |
| SmaSNP154 | UL | - | - | XII | 8371602 | 92,41 | 1,00E-49 |
| Sma-USC83 | UL | - | - | XVI | 10351609 | 92,36 | 2,00E-54 |
| SmaUSC-E37 | UL | - | - | XVIII | 10100864 | 95,12 | 8,00E-07 |
| SmaSNP199 | UL | - | - | XX | 11620877 | 92,11 | 2,00E-20 |
| SmaSNP198 | UL | - | - | sc213 | 35939 | 83,95 | 8,00E-07 |

*^a^*(UL): unlinked markers in the turbot map; *^b^*(FW and LOD<3) Framework markers mapped at LOD>3.0 and markers mapped at LOD<3, repectively; (Acc) most likely position of accessory markers ordered at LOD<2; *^c^*(sc): scaffolds of the stickleback or medaka genome.
