# Supplementary material for: An Expressed Sequence Tag (EST)-enriched genetic map of turbot (Scophthalmus maximus): a useful framework for comparative genomics across model and farmed teleosts
Source: BMC Genet. 2012 Jul 2;13:54. doi: 10.1186/1471-2156-13-54 (PMC3464660; doi:10.1186/1471-2156-13-54)
Supplement: Additional file 9 — Table S5. Putative syntenic markers (60) between the turbot genetic map and four model Acanthopterygii genomes. [file 1471-2156-13-54-S9.docx]

| **Table S5. Putative syntenic markers (60) between the turbot and four model Acanthopterygii genomes** | | | | | | | | | | | | | | | | |
| --- | --- | --- | --- | --- | --- | --- | --- | --- | --- | --- | --- | --- | --- | --- | --- | --- |
|  | **Turbot** | | |  | **Stickleback** | | |  | **Tetraodon** | |  | **Medaka** | |  | **Fugu** | |
| **Loci** | **LG***^a^* | **Distance cM** | **Marker type***^b^* |  | **Chromo***^c^* | **Location pb** | **Ensembl Transcript ID** |  | **Chromo***^d^* | **Location pb** |  | **Chromo***^c^* | **Location pb** |  | **Chromo***^d^* | **Location pb** |
| SmaUSC-E42 | LG01 | 0 | Lod<3.0 |  | sc120 | 102967 | - |  | 15un | 120964 |  | 17 | 10764804 |  | 22un | 2251714 |
| Sma-USC1 | LG01 | 14,356 | Framework |  | XVIII | 2128742 | - |  | 14 | 8720801 |  | 24 | 3626316 |  | 16 | 2610541 |
| Sma-USC15 | LG01 | 44,802 | Framework |  | III | 965371 | - |  | 15 | 6774962 |  | 17 | 26453109 |  | 22un | 621509 |
| 1/4AC18 | LG01 | 52,263 | Framework |  | III | 6950778 | - |  | 15 | 5105855 |  | 17 | 23666633 |  | 22 | 7569147 |
| SmaSNP204 | LG01 | 60,345 | Framework |  | III | 2913770 | ENSGACT00000018695 |  | 15 | 2315268 |  | 17 | 17870196 |  | 22un | 890367 |
| Sma-USC222 | LG01 | 91,456 | Lod<3.0 |  | X | 13910989 | - |  | 21 | 4805235 |  | 11 | 28235389 |  | 12 | 4957304 |
| SmaSNP143 | LG02 | 0 | Accessory |  | XX | 10546376 | - |  | 8 | 7447389 |  | 16 | 14422571 |  | 7 | 12027121 |
| SmaSNP145 | LG02 | 0 | Accessory |  | XX | 5186095 | ENSGACT00000007896 |  | 8 | 3331003 |  | 16 | 11395054 |  | 7 | 7660574 |
| Sma-USC46 | LG02 | 9,183 | Framework |  | XX | 6052607 | - |  | 8 | 3968345 |  | 16 | 10118557 |  | 7 | 8350158 |
| SmaUSC-E34 | LG03 | 36,826 | Framework |  | XV | 8879246 | - |  | 10 | 8462258 |  | 22 | 7287861 |  | 2 | 4124506 |
| Sma-USC157 | LG03 | 37,987 | Framework |  | XV | 9346526 | - |  | 10 | 8088794 |  | 22 | 6734007 |  | 2 | 4520118 |
| Sma-E118 | LG03 | 52,302 | Framework |  | XV | 5362730 | ENSGACT00000010658 |  | 10 | 5766197 |  | 20 | 16314259 |  | 2 | 7005042 |
| Sma-E72 | LG03 | 65,946 | Framework |  | XV | 13239602 | ENSGACT00000016826 |  | 10 | 1592936 |  | 22 | 19594671 |  | 2 | 1983927 |
| SmaSNP190 | LG04 | 0 | Accessory |  | II | 21371214 | ENSGACT00000022794 |  | 5 | 10773900 |  | 3 | 8987100 |  | 13 | 10332413 |
| SmaSNP181 | LG04 | 19,13 | Framework |  | II | 10572988 | ENSGACT00000020830 |  | 5 | 6105296 |  | 3 | 18938435 |  | 13 | 15299117 |
| Sma-USC100 | LG04 | 19,791 | Framework |  | II | 10507861 | ENSGACT00000020814 |  | 5 | 6147269 |  | 3 | 18859491 |  | 13 | 15244632 |
| Sma-USC102 | LG04 | 19,889 | Framework |  | II | 10500045 | ENSGACT00000020814 |  | 5 | 6152463 |  | 3 | 18851564 |  | 13 | 15239587 |
| Sma-USC12 | LG05 | 45,328 | Framework |  | VIII | 15372758 | ENSGACT00000016041 |  | 1 | 14453911 |  | 4 | 7245563 |  | 20 | 5626945 |
| Sma-USC265 | LG05 | 51,886 | Framework |  | VIII | 19294004 | - |  | 1 | 12613707 |  | 4 | 27708058 |  | 20 | 3651465 |
| SmaUSC-E7 | LG06 | 0 | Accessory |  | XIX | 8761129 | ENSGACT00000010096 |  | 13 | 12434732 |  | uc72 | 1284028 |  | 9 | 13866022 |
| Sma-USC110 | LG06 | 37,626 | Framework |  | XIX | 17112240 | - |  | 13 | 3347723 |  | 6 | 13721612 |  | 9 | 1147059 |
| SmaUSC-E29 | LG06 | 40,181 | Framework |  | XIX | 11926555 | - |  | 13 | 7783797 |  | 6 | 11812359 |  | 9 | 8819606 |
| Sma-E315 | LG06 | 40,217 | Framework |  | XIX | 11926555 | - |  | 13 | 7783797 |  | 6 | 11812359 |  | 9 | 8819606 |
| Sma-E100 | LG07 | 14,066 | Framework |  | IV | 15512412 | ENSGACT00000024555 |  | 1 | 4110195 |  | 10 | 18860933 |  | 14 | 8177789 |
| Sma-USC272 | LG07 | 27,086 | Framework |  | IV | 15613026 | - |  | 1 | 4184957 |  | 10 | 24049370 |  | 14 | 8260061 |
| Sma-E194 | LG07 | 43,478 | Lod<3.0 |  | IV | 2044922 | ENSGACT00000021857 |  | 20 | 1909156 |  | uc115 | 2233993 |  | 14 | 10739602 |
| SMAC08 | LG08 | 12,617 | Framework |  | VII | 17908022 | ENSGACT00000027009 |  | 7 | 5784675 |  | 14 | 16224337 |  | 15 | 9435259 |
| SmaUSC-E43 | LG08 | 14,268 | Framework |  | VII | 14937017 | - |  | 7 | 2925528 |  | 14 | 21443533 |  | 15un | 3114472 |
| Sma-USC269 | LG08 | 29,395 | Lod<3.0 |  | VII | 18830763 | - |  | 7 | 8109227 |  | 14 | 8198534 |  | 15 | 6983534 |
| Sma-USC170 | LG08 | 29,442 | Framework |  | XIV | 735843 | - |  | 4 | 4475263 |  | 12 | 2230060 |  | 6 | 5758792 |
| SmaUSC-E36 | LG09 | 21,202 | Lod<3.0 |  | IX | 5320522 | ENSGACT00000022226 |  | 18 | 9024717 |  | 1 | 24963058 |  | 17 | 6644872 |
| Sma-USC126 | LG09 | 26,947 | Framework |  | IX | 8463766 | - |  | 18 | 6585156 |  | 1 | 19501025 |  | 17 | 9233883 |
| Sma-E71 | LG09 | 31,768 | Framework |  | IX | 1724967 | ENSGACT00000021390 |  | 18 | 321515 |  | 1 | 16890718 |  | 17 | 3588511 |
| Sma-E139 | LG09 | 37,583 | Framework |  | XVIII | 2150149 | ENSGACT00000006932 |  | 14 | 8673747 |  | 24 | 3594886 |  | 16 | 2665868 |
| Sma-E302 | LG09 | 38,104 | Framework |  | IX | 17521129 | - |  | 18 | 4849146 |  | 1 | 11949965 |  | 17 | 10856154 |
| SmaUSC-E41 | LG09 | 40,111 | Framework |  | IX | 12938250 | ENSGACT00000024549 |  | 18 | 1566960 |  | 1 | 33957271 |  | 17un | 1602227 |
| Sma-USC21 | LG09 | 52,331 | Framework |  | IX | 15948559 | - |  | 18 | 5719161 |  | 1 | 32720587 |  | 17 | 10009136 |
| Sma-USC217 | LG10 | 10,065 | Framework |  | sc68 | 560174 | - |  | 9 | 7039926 |  | 7 | 18031409 |  | 3 | 10188364 |
| Sma-USC266 | LG12 | 60,323 | Framework |  | XIII | 18844207 | ENSGACT00000018946 |  | 12 | 3505704 |  | 9 | 28839366 |  | 21 | 12466737 |
| Sma-E120 | LG13 | 47,161 | Framework |  | XI | 14085801 | ENSGACT00000018562 |  | 3 | 12419481 |  | 8 | 7602415 |  | 5 | 9219748 |
| SmaUSC-E38 | LG13 | 58,91 | Framework |  | XI | 5665417 | - |  | 3 | 12989025 |  | 8 | 6474909 |  | 5 | 9821020 |
| SmaSNP200 | LG14 | 0 | Accessory |  | XIV | 7786674 | ENSGACT00000023088 |  | 4 | 1145426 |  | 12 | 15396161 |  | 6un | 1259137 |
| 3/20CA17 | LG16 | 43,462 | Framework |  | IV | 17827523 | - |  | 19 | 3911247 |  | 23 | 13496968 |  | 18 | 5782469 |
| Sma-USC285 | LG16 | 47,255 | Framework |  | IV | 28508290 | - |  | 19 | 3812511 |  | 23 | 13722102 |  | 18 | 5646844 |
| Sma-USC223 | LG16 | 54,141 | Framework |  | IV | 30161452 | - |  | 19 | 2534013 |  | 23 | 16224097 |  | 18 | 4343930 |
| Sma-E183 | LG16 | 57,157 | Framework |  | IV | 30485838 | - |  | 19 | 2318276 |  | 23 | 16761651 |  | 18 | 4112868 |
| 3/20CA17 | LG19 | 0 | Framework |  | IV | 17827523 | - |  | 19 | 3911247 |  | 23 | 13496968 |  | 18 | 5782469 |
| SmaSNP28 | LG19 | 0 | Accessory |  | VI | 3639999 | ENSGACT00000004885 |  | 17 | 1531598 |  | 15 | 28600564 |  | 4 | 2699542 |
| Sma-E244 | LG20 | 19,871 | Framework |  | XVI | 2373553 | ENSGACT00000002222 |  | 2 | 10601285 |  | 21 | 10191812 |  | 1 | 12014324 |
| SmaSNP210 | LG21 | 0 | Framework |  | XX | 10546376 | ENSGACT00000011934 |  | 8 | 7447389 |  | 16 | 14422571 |  | 7 | 12027121 |
| SmaSNP141 | LG22 | 0 | Accessory |  | X | 3210868 | ENSGACT00000003762 |  | 21un | 3163952 |  | 11 | 4617793 |  | 12 | 10652780 |
| Sma-E168 | LG23 | 0,429 | Framework |  | XVIII | 8596787 | ENSGACT00000011810 |  | 14 | 3629752 |  | 24 | 7054000 |  | 16 | 9600013 |
| Sma-USC273 | LG23 | 11,2 | Framework |  | XVIII | 2314947 | - |  | 14 | 8527683 |  | 24 | 3308825 |  | 16 | 2819317 |
| Sma-USC38 | LG23 | 20,119 | Framework |  | XVIII | 10968753 | - |  | 14 | 5925177 |  | 24 | 14014700 |  | 16 | 5606661 |
| Sma-E127 | LG23 | 28,875 | Framework |  | XVIII | 8941163 | ENSGACT00000012237 |  | 14 | 3907840 |  | 24 | 6605869 |  | 16 | 9309843 |
| SmaSNP131 | UL | - | - |  | VII | 24063617 | ENSGACT00000027494 |  | 7 | 10785502 |  | 14 | 2445723 |  | 15 | 2872044 |
| SmaSNP154 | UL | - | - |  | XII | 8371602 | ENSGACT00000008804 |  | 9 | 1631816 |  | 7 | 28771422 |  | 3 | 4611079 |
| SmaSNP89 | UL | - | - |  | I | 23735780 | - |  | 3 | 4573372 |  | 2 | 2716355 |  | 8 | 3333200 |

*^a^*(UL): unlinked markers in the turbot map; *^b^*(FW and LOD<3) Framework markers mapped at LOD>3.0 and markers mapped at LOD<3, repectively; (Acc) most likely position of accessory markers ordered at LOD<2; *^c^*(sc): scaffolds of the stickleback or medaka genome; (uc) ultracontigs of medaka genome; *^d^*(un): unrandom sequences of specific chromosomes or unassigned genomic regions of *Tetraodon* and fugu.
