# Supplementary material for: An Expressed Sequence Tag (EST)-enriched genetic map of turbot (Scophthalmus maximus): a useful framework for comparative genomics across model and farmed teleosts
Source: BMC Genet. 2012 Jul 2;13:54. doi: 10.1186/1471-2156-13-54 (PMC3464660; doi:10.1186/1471-2156-13-54)
Supplement: Additional file 10 — Table S6. Conservation of multiple similarity hits between the turbot and four model Acanthopterygii genomes. [file 1471-2156-13-54-S10.docx]

**Table S6. Conservation of multiple similarity hits between the turbot and four model Acanthopterygii genomes (19 markers)**

|  |  | **Stickleback** | | | | |  | **Medaka** | | | | |  | ***Tetraodon*** | | | | |  | **Fugu** | | | | |
| --- | --- | --- | --- | --- | --- | --- | --- | --- | --- | --- | --- | --- | --- | --- | --- | --- | --- | --- | --- | --- | --- | --- | --- | --- |
| **Marker** | **LG***^a^* | **Chrom***^b^* | **ID**  **%** | **Evalue** | **Location**  **bp** | **Length**  **bp** |  | **Chrom***^b^* | **ID**  **%** | **Eval** | **Location**  **bp** | **Length**  **bp** |  | **Chrom***^c^* | **ID**  **%** | **Evalue** | **Location**  **bp** | **Length**  **bp** |  | **Chrom***^c^* | **ID**  **%** | **Evalue** | **Location**  **bp** | **Length**  **bp** |
| Sma-E128 | LG20 | XVI | 89,73 | 2,00E-54 | 7729800 | 185 |  | 2 | 94,23 | 6,00E-37 | 28692212 | 104 |  | 2 | 89,39 | 7,00E-35 | 11687998 | 132 |  | 1 | 89,39 | 7,00E-35 | 13185957 | 132 |
|  |  | I | 95,19 | 1,00E-39 | 25191299 | 104 |  | 21 | 90,35 | 6,00E-31 | 5239996 | 114 |  | 3 | 92,31 | 2,00E-32 | 6020807 | 104 |  | 8 | 90,38 | 1,00E-27 | 1897823 | 104 |
| Sma-E74 | LG13 | XI | 88,45 | 1,00E-86 | 16242517 | 303 |  | 8 | 82,49 | 3,00E-42 | 2454330 | 297 |  | 2 | 84,55 | 1,00E-45 | 238015 | 246 |  | 1un | 81,85 | 5,00E-30 | 1221285 | 248 |
|  |  | V | 86,41 | 2,00E-67 | 2323273 | 287 |  | sc402 | 82,61 | 7,00E-31 | 3169 | 230 |  | 3 | 81,61 | 1,00E-36 | 14454254 | 299 |  | 5un | 80,95 | 3,00E-31 | 698253 | 294 |
| Sma-E99 | LG09 | IX | 95,65 | 4,00E-46 | 18508007 | 115 |  | 1 | 91,96 | 2,00E-34 | 10650455 | 112 |  | Un | 92,17 | 1,00E-36 | 54264279 | 115 |  | 17 | 90,18 | 5,00E-30 | 4512737 | 112 |
|  |  | sc277 | 91,76 | 2,00E-23 | 12406 | 85 |  | 15 | 88 | 4,00E-20 | 11001637 | 100 |  | 17 | 88 | 9,00E-13 | 10023033 | 75 |  | 4 | 90,67 | 2,00E-17 | 5842573 | 75 |
| Sma-USC20 | LG12 | XIII | 94,59 | 2,00E-56 | 7943891 | 148 |  | 9 | 91,55 | 4,00E-43 | 11160457 | 142 |  | 12 | 87,66 | 6,00E-34 | 7194494 | 154 |  | 21 | 88,89 | 5,00E-41 | 8672543 | 162 |
|  |  | XIV | 87,18 | 2,00E-12 | 3337219 | 78 |  | 4 | 87,65 | 7,00E-14 | 32592041 | 81 |  | 4 | 90,67 | 8,00E-18 | 6987749 | 75 |  | 6un | 90,67 | 9,00E-18 | 1366539 | 75 |
|  |  | - | - | - | - | - |  | 12 | 84,42 | 1,00E-06 | 7606314 | 77 |  | - | - | - | - | - |  | - | - | - | - | - |
| SmaSNP1 | LG06 | XIX | 85,25 | 1,00E-42 | 7393265 | 217 |  | 6 | 84,06 | 6,00E-34 | 10320966 | 207 |  | 13 | 87,56 | 2,00E-47 | 11619461 | 193 |  | 13 | 87,5 | 2,00E-20 | 11332877 | 104 |
|  |  | II | 89,04 | 7,00E-14 | 18041000 | 73 |  | 3 | 90,91 | 9,00E-27 | 12011158 | 99 |  | 5 | 89,13 | 1,00E-20 | 9881580 | 92 |  | 9 | 88,83 | 1,00E-48 | 12972525 | 179 |
| SmaSNP178 | LG02 | XX | 86,01 | 3,00E-53 | 654650 | 243 |  | 16 | 84,36 | 2,00E-43 | 18746726 | 243 |  | 8 | 87,13 | 9,00E-41 | 804303 | 199 |  | 13 | 87,72 | 1,00E-06 | 7157507 | 57 |
|  |  | II | 87,3 | 8,00E-08 | 23290870 | 63 |  | 3 | 89,29 | 4,00E-08 | 4151653 | 56 |  | 5 | 87,1 | 2,00E-07 | 13224308 | 62 |  | 7 | 89,89 | 5,00E-21 | 10467068 | 89 |
|  |  | - | - | - | - | - |  | - | - | - | - | - |  | - | - | - | - | - |  | 7un | 88,4 | 2,00E-69 | 1492503 | 250 |
|  |  | - | - | - | - | - |  | - | - | - | - | - |  | - | - | - | - | - |  | un | 89,89 | 5,00E-21 | 48312526 | 89 |
| SmaSNP44 | LG13 | XI | 93,6 | 8,00E-45 | 6526628 | 125 |  | 19 | 92 | 9,00E-40 | 8571112 | 125 |  | 3 | 94,55 | 1,00E-40 | 13518453 | 110 |  | 1 | 84,8 | 1,00E-18 | 4422725 | 125 |
|  |  | V | 90,4 | 3,00E-35 | 8059510 | 125 |  | 8 | 91,27 | 6,00E-38 | 5387107 | 126 |  | 2un | 87,88 | 8,00E-20 | 159686 | 99 |  | 5 | 94,5 | 4,00E-40 | 10421199 | 109 |
| Sma-E158 | LG16 | VII | 92,59 | 1,00E-34 | 797646 | 108 |  | 18 | 86,96 | 2,00E-15 | 21249566 | 92 |  | Un | 96,43 | 1,00E-46 | 36237483 | 112 |  | 8 | 92,22 | 1,00E-21 | 11193731 | 90 |
|  |  | I | 83,52 | 8,00E-08 | 27637246 | 91 |  | 2 | 83,7 | 4,00E-08 | 17001269 | 92 |  | 3 | 86,81 | 4,00E-15 | 1520629 | 91 |  | 8un | 83,33 | 7,00E-11 | 1910338 | 108 |
| Sma-USC9 | LG13 | XI | 91,09 | 2,00E-28 | 2372540 | 101 |  | 8 | 91,23 | 1,00E-33 | 23241840 | 114 |  | Un | 91,96 | 4,00E-35 | 4963367 | 112 |  | 1 | 88,89 | 4,00E-26 | 501873 | 116 |
|  |  | V | 88,89 | 2,00E-25 | 3418514 | 108 |  | 19 | 92,39 | 1,00E-27 | 15118983 | 92 |  | 2 | 88,89 | 1,00E-25 | 916191 | 108 |  | 5 | 92,86 | 2,00E-37 | 1286725 | 112 |
|  |  | X | 86,49 | 7,00E-10 | 9323460 | 74 |  | - | - | - | - | - |  | - | - | - | - | - |  | - | - | - | - | - |
| SmaSNP113 | LG12 | XIII | 91,3 | 5,00E-43 | 11660203 | 138 |  | 9 | 88,24 | 5,00E-32 | 16262387 | 136 |  | 12 | 92,86 | 3,00E-25 | 1033524 | 84 |  | 21 | 88,19 | 2,00E-29 | 14868879 | 127 |
|  |  | III | 89,83 | 3,00E-10 | 16069583 | 59 |  | 7 | 84,62 | 5,00E-07 | 8459748 | 78 |  | 9 | 87,01 | 2,00E-11 | 1635033 | 77 |  | 3 | 85,9 | 1,00E-09 | 4614183 | 78 |
|  |  | XII | 86,76 | 2,00E-08 | 8376496 | 68 |  | - | - | - | - | - |  | - | - | - | - | - |  | - | - | - | - | - |
| SmaSNP202 | LG22 | X | 91,53 | 1,00E-105 | 1559304 | 295 |  | 11 | 92,54 | 1,00E-112 | 16671299 | 295 |  | 21 | 89,04 | 3,00E-87 | 525171 | 292 |  | 12 | 88,36 | 2,00E-82 | 558649 | 292 |
|  |  | IX | 89,07 | 8,00E-51 | 9893772 | 183 |  | 15 | 86,98 | 2,00E-37 | 10072107 | 169 |  | 17 | 88,76 | 6,00E-45 | 10428545 | 169 |  | 4 | 89,94 | 1,00E-49 | 5393669 | 169 |
|  |  | XXI | 87,67 | 2,00E-11 | 9927885 | 73 |  | sc871 | 79,75 | 6,00E-07 | 53227 | 158 |  | 6 | 84,08 | 2,00E-23 | 5683112 | 157 |  | 10 | 81,42 | 2,00E-17 | 6454826 | 183 |
|  |  | - | - | - | - | - |  | - | - | - | - | - |  | 18 | 87,1 | 2,00E-07 | 1258605 | 62 |  | - | - | - | - | - |
| Sma-E174 | UL | V | 88,65 | 2,00E-35 | 8336737 | 141 |  | 19 | 92,92 | 2,00E-37 | 4342522 | 113 |  | 20 | 89,33 | 3,00E-15 | 651238 | 75 |  | 1 | 87,41 | 2,00E-29 | 10105783 | 135 |
|  |  | VII | 86,11 | 1,00E-08 | 4423181 | 72 |  | 10 | 91,11 | 2,00E-06 | 25015748 | 45 |  | 18 | 87,67 | 1,00E-11 | 8512215 | 73 |  | 17 | 84,72 | 3,00E-06 | 7193611 | 72 |
|  |  | IX | 86,11 | 1,00E-08 | 5969276 | 72 |  | - | - | - | - | - |  | - | - | - | - | - |  | 8 | 88 | 8,00E-13 | 13152041 | 75 |
| Sma-E52 | LG03 | XV | 88,89 | 1,00E-25 | 1413239 | 108 |  | 22 | 95,37 | 4,00E-42 | 25461369 | 108 |  | 10 | 92,59 | 2,00E-35 | 12250238 | 108 |  | 14 | 85,23 | 2,00E-11 | 471879 | 88 |
|  |  | XIII | 88,66 | 2,00E-21 | 12370737 | 97 |  | 9 | 87,23 | 4,00E-17 | 17278322 | 94 |  | Un | 87,63 | 3,00E-19 | 32159575 | 97 |  | 2un | 89,81 | 3,00E-28 | 856625 | 108 |
|  |  | IV | 85,71 | 3,00E-13 | 4899434 | 91 |  | 10 | 87,32 | 2,00E-10 | 22969009 | 71 |  | 1 | 85,23 | 2,00E-11 | 9886474 | 88 |  | 21 | 84,54 | 5,00E-12 | 365122 | 97 |
|  |  | sc48 | 85,23 | 2,00E-11 | 1707221 | 88 |  | 15 | 85,14 | 2,00E-07 | 17650552 | 74 |  | - | - | - | - | - |  | un | 85,14 | 3,00E-07 | 48385026 | 74 |
| Sma-USC7 | LG04 | II | 88,43 | 1,00E-165 | 4566258 | 553 |  | 3 | 86,71 | 1,00E-142 | 31758606 | 557 |  | Un | 88,68 | 1,00E-163 | 93597960 | 539 |  | 13 | 86,65 | 1,00E-143 | 2782878 | 562 |
|  |  | XIX | 82,61 | 4,00E-54 | 16915354 | 368 |  | 6 | 83,82 | 1,00E-24 | 13960192 | 173 |  | 13 | 83,45 | 5,00E-47 | 3533505 | 290 |  | 20 | 92 | 4,00E-29 | 7464548 | 100 |
|  |  | III | 95 | 1,00E-26 | 12399341 | 80 |  | sc908 | 95,71 | 9,00E-23 | 60913 | 70 |  | 1 | 90,48 | 2,00E-27 | 16153612 | 105 |  | 22 | 87,32 | 2,00E-09 | 3043686 | 71 |
|  |  | VIII | 85,71 | 4,00E-17 | 13177005 | 112 |  | 17 | 88,61 | 8,00E-14 | 31468442 | 79 |  | 15un | 90 | 1,00E-13 | 2870471 | 70 |  | 9 | 81,65 | 5,00E-47 | 1332429 | 376 |
| Sma-E261 | LG15 | I | 92,98 | 3,00E-38 | 21722517 | 114 |  | 2 | 94,44 | 7,00E-31 | 20205421 | 90 |  | 3 | 92,73 | 5,00E-36 | 1701080 | 110 |  | 12 | 87,27 | 1,00E-21 | 342272 | 110 |
|  |  | X | 89,61 | 3,00E-16 | 960373 | 77 |  | sc1128 | 92,11 | 4,00E-20 | 14841 | 76 |  | 8 | 90 | 1,00E-21 | 9557371 | 90 |  | 7 | 85,71 | 4,00E-09 | 13908107 | 77 |
|  |  | III | 86 | 1,00E-15 | 15244761 | 100 |  | 11 | 89,29 | 1,00E-17 | 16017117 | 84 |  | Un | 91,55 | 2,00E-17 | 72742461 | 71 |  | 8 | 91,82 | 1,00E-33 | 6261971 | 110 |
|  |  | XX | 87,67 | 2,00E-11 | 11274261 | 73 |  | 17 | 84,87 | 1,00E-14 | 2563971 | 119 |  | - | - | - | - | - |  | un | 83,02 | 2,00E-07 | 25345902 | 105 |
|  |  | - | - | - | - | - |  | 16 | 87,67 | 4,00E-11 | 17858046 | 73 |  | - | - | - | - | - |  | - | - | - | - | - |
| SmaSNP9 | LG02 | I | 89,72 | 4,00E-27 | 27338213 | 107 |  | 13 | 80,84 | 1,00E-25 | 18141508 | 261 |  | 16 | 82,76 | 3,00E-27 | 7181956 | 203 |  | 11 | 84,19 | 1,00E-36 | 10327576 | 215 |
|  |  | VIII | 90,43 | 4,00E-24 | 4957436 | 94 |  | uc36 | 94,52 | 3,00E-23 | 37660 | 73 |  | 1 | 90,32 | 1,00E-23 | 22001071 | 93 |  | 20 | 86,17 | 1,00E-14 | 12740837 | 94 |
|  |  | sc1251 | 90,43 | 4,00E-24 | 3739 | 94 |  | 4 | 87,78 | 1,00E-16 | 22048019 | 90 |  | Un | 82,46 | 5,00E-20 | 20590852 | 171 |  | 22 | 83,7 | 1,00E-08 | 3476080 | 92 |
|  |  | III | 81,29 | 4,00E-15 | 13180133 | 171 |  | 10 | 95,12 | 6,00E-09 | 8675780 | 41 |  | 3 | 88,17 | 8,00E-19 | 25219 | 93 |  | 8 | 89,41 | 8,00E-19 | 8499017 | 85 |
|  |  | XVIII | 87,69 | 3,00E-09 | 6670507 | 65 |  | 17 | 84,42 | 2,00E-06 | 30133378 | 77 |  | 14 | 89,8 | 7,00E-07 | 4727134 | 49 |  | - | - | - | - | - |
| Sma-E220 | LG10 | XII | 97,25 | 3,00E-47 | 9888087 | 109 |  | sc3796 | 91,09 | 7,00E-28 | 4032 | 101 |  | 20 | 94,44 | 1,00E-39 | 524009 | 108 |  | 12un | 90,36 | 7,00E-20 | 1403236 | 83 |
|  |  | sc129 | 94,06 | 2,00E-35 | 41975 | 101 |  | 11 | 90,48 | 4,00E-20 | 15072095 | 84 |  | Un | 92,68 | 4,00E-24 | 54095287 | 82 |  | 19 | 89,58 | 4,00E-06 | 5804208 | 48 |
|  |  | XX | 91,67 | 8,00E-23 | 11642101 | 84 |  | 16 | 88,64 | 9,00E-18 | 17364725 | 88 |  | 11 | 88,89 | 3,00E-19 | 6119956 | 90 |  | 3 | 94,5 | 3,00E-40 | 5890202 | 109 |
|  |  | X | 91,67 | 8,00E-23 | 14544122 | 84 |  | 7 | 92,65 | 9,00E-18 | 10559002 | 68 |  | - | - | - | - | - |  | 7 | 90,36 | 7,00E-20 | 13573183 | 83 |
|  |  | sc27 | 88,89 | 3,00E-19 | 4349284 | 90 |  | 5 | 88,51 | 4,00E-17 | 3045372 | 87 |  | - | - | - | - | - |  | 8un | 90,1 | 7,00E-26 | 3167380 | 101 |
| Sma-E255 | LG20 | XVI | 87,27 | 1,00E-37 | 11742422 | 165 |  | 21 | 92,44 | 1,00E-38 | 21953946 | 119 |  | 2 | 87,8 | 1,00E-39 | 15024935 | 164 |  | 1 | 89,93 | 2,00E-42 | 16625340 | 149 |
|  |  | III | 85,98 | 2,00E-17 | 14445698 | 107 |  | 20 | 85,45 | 2,00E-16 | 7351698 | 110 |  | 9 | 88,42 | 7,00E-20 | 3254382 | 95 |  | 10 | 83,06 | 3,00E-13 | 1182833 | 124 |
|  |  | XXI | 85,23 | 8,00E-11 | 1641756 | 88 |  | 17 | 84,16 | 4,00E-11 | 7483503 | 101 |  | 15 | 87,37 | 2,00E-17 | 999692 | 95 |  | 19 | 84,04 | 1,00E-09 | 4260942 | 94 |
|  |  | sc27 | 84,04 | 1,00E-09 | 1784066 | 94 |  | - | - | - | - | - |  | 11 | 86,52 | 7,00E-14 | 7488128 | 89 |  | 22 | 87,37 | 2,00E-17 | 11518680 | 95 |
|  |  | XII | 83,16 | 8,00E-08 | 10485249 | 95 |  | - | - | - | - | - |  | Un | 86,52 | 7,00E-14 | 111731077 | 89 |  | 3 | 84,73 | 8,00E-20 | 6372718 | 131 |
| SmaSNP137 | UL | XV | 91,76 | 3,00E-62 | 885998 | 182 |  | 22 | 92,15 | 2,00E-67 | 20960486 | 191 |  | 10 | 93,67 | 5,00E-60 | 12901708 | 158 |  | 11 | 81,22 | 2,00E-16 | 9657944 | 181 |
|  |  | I | 86,19 | 8,00E-38 | 15917470 | 181 |  | 13 | 85,47 | 6,00E-34 | 3295240 | 179 |  | 16 | 88 | 7,00E-44 | 6589195 | 175 |  | 15un | 83,92 | 5,00E-20 | 3287131 | 143 |
|  |  | VII | 85,08 | 5,00E-33 | 12661367 | 181 |  | sc1003 | 84,28 | 2,00E-24 | 20767 | 159 |  | Un | 88,24 | 6,00E-38 | 37063955 | 153 |  | 16 | 87,76 | 2,00E-19 | 821748 | 98 |
|  |  | XVIII | 83,67 | 6,00E-20 | 16122610 | 147 |  | 14 | 81,46 | 1,00E-16 | 815664 | 178 |  | 14 | 89,06 | 2,00E-32 | 10182530 | 128 |  | 2 | 89,94 | 2,00E-53 | 10997691 | 179 |
|  |  | sc168 | 91,94 | 2,00E-14 | 141285 | 62 |  | 12 | 90,32 | 7,00E-12 | 783612 | 62 |  | 13 | 95,83 | 2,00E-13 | 12909328 | 48 |  | 6 | 86,44 | 1,00E-05 | 6223847 | 59 |
|  |  | XIII | 88,71 | 9,00E-10 | 4444731 | 62 |  | 6 | 87,1 | 4,00E-07 | 2989299 | 62 |  | 12 | 90 | 1,00E-08 | 3341030 | 59 |  | 9 | 91,67 | 1,00E-08 | 5046235 | 48 |
|  |  | XIX | 93,18 | 1,00E-08 | 8179814 | 44 |  | - | - | - | - | - |  | - | - | - | - | - |  | - | - | - | - | - |

*^a^* (UL): unlinked markers in the turbot map; *^b^*(sc): scaffolds of the stickleback or medaka genome; (uc) ultracontigs of medaka genome; *^c^* (un): unrandom sequences of specific chromosomes or unassigned genomic regions of *Tetraodon* and fugu.
